# From 2-Triethylammonium Ethyl Ether of 4-Stilbenol (MG624) to Selective Small-Molecule Antagonists of Human α9α10 Nicotinic Receptor by Modifications at the Ammonium Ethyl Residue

**DOI:** 10.1021/acs.jmedchem.2c00746

**Published:** 2022-07-14

**Authors:** Francesco Bavo, Marco Pallavicini, Susanna Pucci, Rebecca Appiani, Alessandro Giraudo, Brek Eaton, Linda Lucero, Cecilia Gotti, Milena Moretti, Paul Whiteaker, Cristiano Bolchi

**Affiliations:** † Dipartimento di Scienze Farmaceutiche, Università degli Studi di Milano, via Mangiagalli 25, I-20133 Milano, Italy; ‡ Department of Drug Design and Pharmacology, University of Copenhagen, DK-2100 Copenhagen, Denmark; § Institute of Neuroscience, CNR, via Vanvitelli 32, I-20129 Milano, Italy; ∥ NeuroMi Milan Center for Neuroscience, University of Milano Bicocca, piazza Ateneo Nuovo 1, I-20126 Milano, Italy; ⊥ Division of Neurobiology, Barrow Neurological Institute, Phoenix, Arizona 85013, United States; # Department of Medical Biotechnology and Translational Medicine, Università degli Studi di Milano, via Vanvitelli 32, I-20129 Milano, Italy; ∇ Department of Pharmacology and Toxicology, Medical College of Virginia Campus, Virginia Commonwealth University, Richmond, Virginia 23298, United States

## Abstract

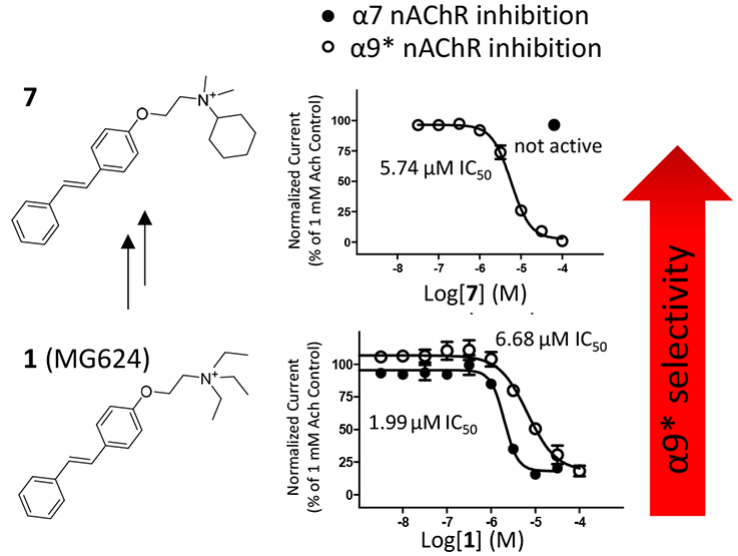

Nicotinic acetylcholine receptors containing α9 subunits (α9*-nAChRs) are potential druggable targets arousing great interest for pain treatment alternative to opioids. Nonpeptidic small molecules selectively acting as α9*-nAChRs antagonists still remain an unattained goal. Here, through modifications of the cationic head and the ethylene linker, we have converted the 2-triethylammonium ethyl ether of 4-stilbenol (MG624), a well-known α7- and α9*-nAChRs antagonist, into some selective antagonists of human α9*-nAChR. Among these, the compound with cyclohexyldimethylammonium head (**7**) stands out for having no α7-nAChR agonist or antagonist effect along with very low affinity at both α7- and α3β4-nAChRs. At supra-micromolar concentrations, **7** and the other selective α9* antagonists behaved as partial agonists at α9*-nAChRs with a very brief response, followed by rebound current once the application is stopped and the channel is disengaged. The small or null postapplication activity of ACh seems to be related to the slow recovery of the rebound current.

## Introduction

Mammalian nonmuscle nicotinic acetylcholine receptors (nAChRs) form as pentameric assemblies of protein subunits (named α2-α7, α9, α10, and β2−β4), with particular subunit combinations termed “subtypes”.^1^ The majority of nonmuscle nAChR subtypes contain at least one α and one β subunit (i.e., are heteromeric), and agonists bind sites formed at interfaces between the (+)-faces of α-subunits and (−)-faces of β-subunits.^1^ However, α7 and α9 subunits appear to be uniquely capable of forming functional homomeric nAChR subtypes, in which a single subunit is capable of providing both the (+)- and (−)-faces needed to bind agonists.^1^ A further similarity is that compounds such as MLA and α-bungarotoxin (α-Bgtx), originally thought to selectively antagonize α7-nAChR, also are similarly potent antagonists of α9-nAChR homopentamers.^2^ Heteropentameric α9α10-nAChRs are often formed and show very similar antagonist pharmacological profiles to α7- and α9-nAChR homopentamers.^3^

Nevertheless, some important features distinguish α7- from α9*-nAChRs (where * denotes the possible presence of additional nAChR subunits, in this case α10).^4^ First, while both are expressed in both neuronal and non-neuronal cells, the expression of α9*-nAChRs is restricted to the periphery, whereas α7-nAChRs expression is widely distributed across CNS and peripheral locations.^5^ Second, nicotine is an α7-nAChR agonist, whereas it is an antagonist of α9*-nAChRs.^3^ Third, α9*-nAChRs exhibit a Ca^2+^ permeability 2-fold higher than α7-nAChRs, with an outstanding fractional Ca^2+^ current of about 22%.^6^ Forth, the physiological effects of α7- vs α9*-nAChR antagonists can be opposing. For example, α9*-nAChR antagonists have been explored as novel, nonopioid, analgesics to treat neuropathic and inflammatory pain.^7^ In contrast, the agonism of peripheral α7-nAChR produces analgesic and anti-inflammatory effects.^7^ This property recently has been extended to α7-nAChR “silent agonists” (ligands that produce very little or no ionotropic agonist activity (if not co-applied with a positive allosteric modulator; PAM), but that instead activate metabotropic signaling pathways through α7-nAChR).^7,8^

**Chart 1:**
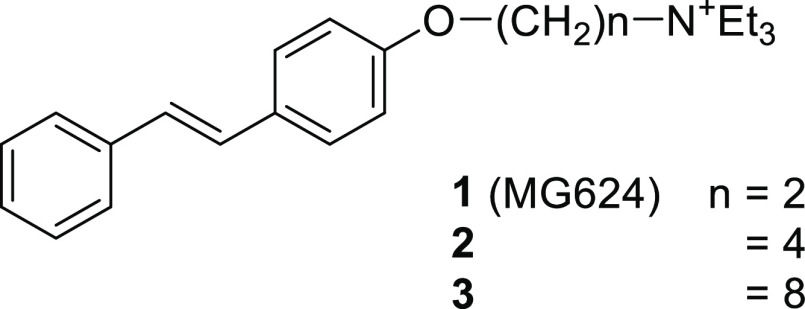
**1** (MG624) and Its Analogues with Elongated N–O Linker

However, opposing effects are not the rule. For example, α7- and α9*-nAChR are overexpressed in multiple tumor types, where activation of either promotes tumor cell growth, and inhibition has antiproliferative effects.^9−11^ For instance, analogues of the 2-triethylammonium ethyl ether of 4-stilbenol (**1**, MG624) with a lengthened alkylene linker between charged nitrogen and ethereal oxygen (**2** and **3**) (Chart 1) display potent antiadenocarcinoma and antiglioblastoma activity, paralleled by increased α7- and α9α10-nAChR antagonism.^12^ More-recent results have suggested dimerization of α-conotoxins capable of inhibiting α9α10-nAChRs and, with lower potency, α7-nAChRs as a strategy to obtain more potent α7 and α9α10 dual inhibitors, which could be useful probes and/or drug leads to investigate the role of these receptors in tumorigenesis.^13^ Indeed, α7- and α9*-nAChR involvement in tumor cell proliferation is consistently supported across a range of published studies,^9,10^ although it is important to note that additional non-nicotinic mechanisms could also contribute to the antitumor activity of some nicotinic antagonists, as demonstrated by a very recent pharmacological investigation on the above stilbenol derivatives **2** and **3**.^10^

Within this context, antagonists selectively targeting α9α10-nAChRs, indispensable for dissecting function of α9α10- vs α7-nAChRs, are very few and those eligible for the development of druglike leads are still lacking. Indeed, when excluding some potent and selective bis-, tris-, and tetrakis-azaaromatic quaternary ammonium analogues^14^ for poor drug-likeness and, for similar reasons, α-conotoxins and related peptides, the literature offers no examples of small molecules that can be considered promising hits for the development of selective α9α10 antagonists.

Our recent investigations proved that pretreatment of α7- and α9α10-nAChRs with **1**, first reported as a selective α7 antagonist in a pioneering study dating from 1998,^15^ potently inhibits the activation of both receptor subtypes by subsequently applied ACh and that elongation of the alkylene chain between the ammonium head and the oxygen by addition of further methylene units (compounds **2** and **3**) results in more potent antagonism.^9,12^

**Chart 2:**
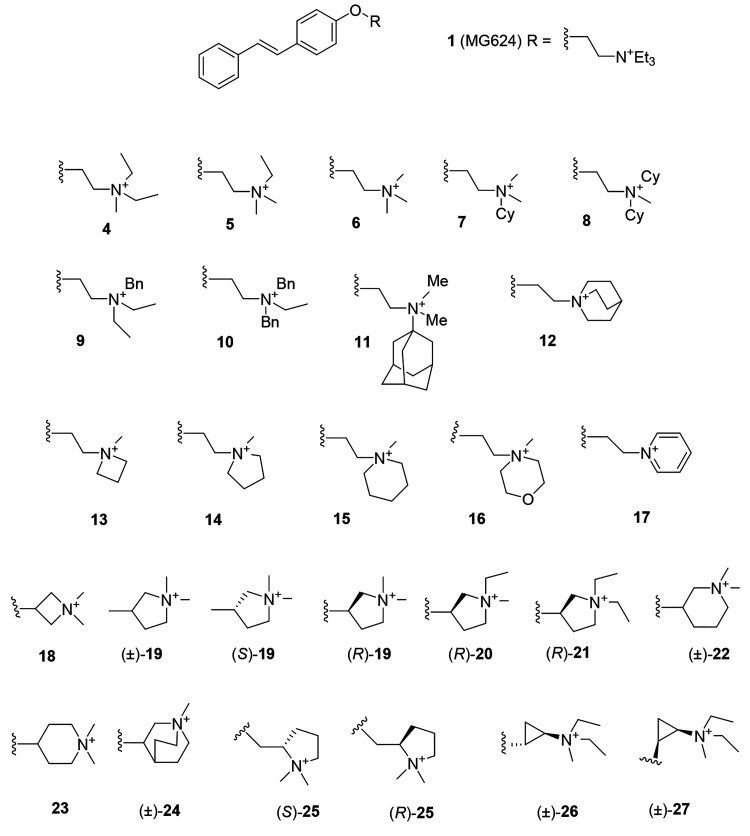
Analogues of **1** Modified at the Ammonium Head (**4**–**17**) and at the O–N Linker (**18**–**27**)

These observations indicate that **1** is a good hit, susceptible to useful changes of pharmacological profile by structural modification. Accordingly, we have extended our structure–activity relationship (SAR) studies in the hope of differentiating its activities at α7- and α9α10-nAChR, from each other. We considered a wide number of modifications of the ammonium head and of the linker on one side, and of alternative scaffolds in replacement of the stilbene substructure on the other. Here, we report the synthesis and binding affinities at the α4β2, α3β4, and α7 subtypes of the analogues of **1** modified at the ammonium head (compounds **4**–**17**) and at the linker (compounds **18**–**27**) (Chart 2). The ammonium head was modified by the increase of its steric bulk through gradual replacement of methyls with ethyls (compounds **4**–**6**), inclusion of two of the three alkyls in increasingly larger cycles (compounds **13**–**16**), replacement of one or two alkyls with cyclic substituents (compounds **7**–**11**), change in shape and positive charge distribution (compounds **12** and **17**). The linker was modified maintaining the interposition of two carbons between oxygen and nitrogen, but enclosing, partially or totally, this fragment into a four or five-membered nitrogen heterocycle (compounds **18**–**22**, **24**, and **25**) or rigidifying it into cyclopropane (compounds **26** and **27**). In compound **23**, three carbons were interposed, but conformationally constrained in the piperidine cycle. A large selection of these analogues, including compounds proved to have higher or remarkably lower α7 affinity than **1**, was then screened for α7- and α9α10-nAChR antagonist activity. Lastly, the four compounds showing the best profiles in terms of potency and subtype selectivity were further characterized to better understand the mechanism by which they exert antagonism.

## Results

### Chemistry

**Scheme 1:**
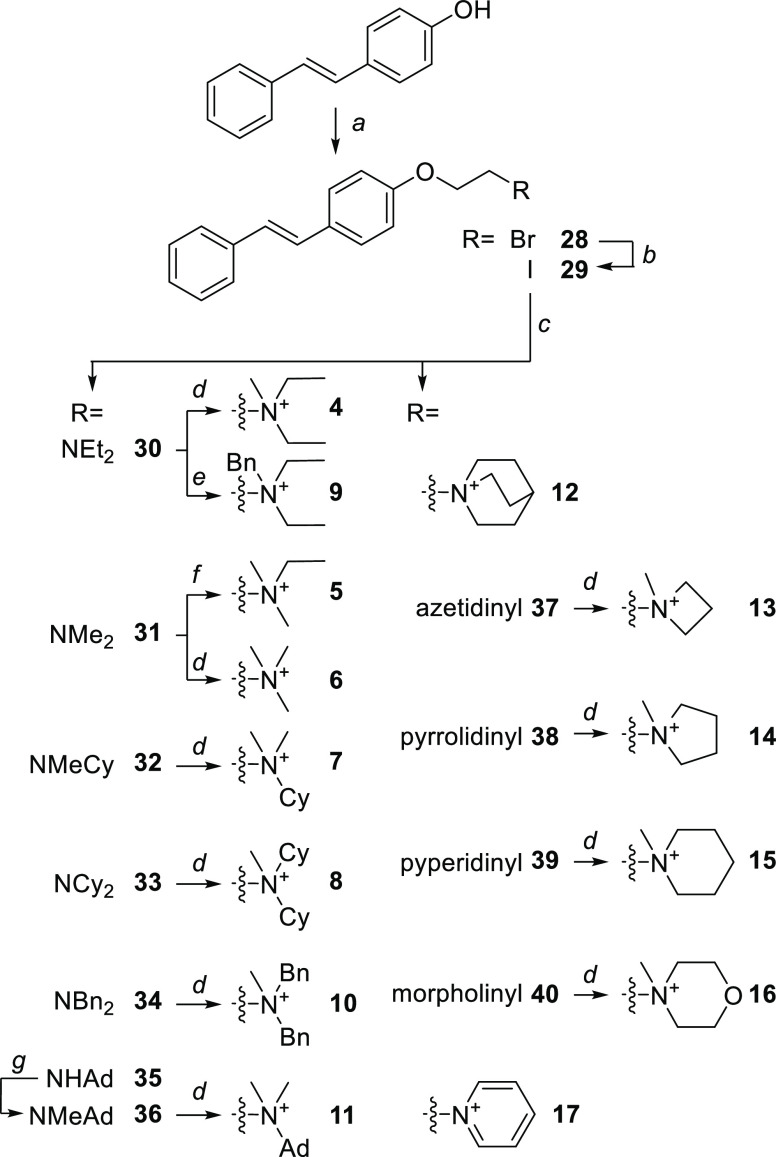
Reagents and Conditions (a) 1,2-Dibromoethane, K_2_CO_3_, KI cat, methylethylketone, 48 h, reflux, 61% (**28**); (b) NaI, acetone, reflux, overnight, 100% (**29**); (c) diethylamine, tetrahydrofuran (THF), reflux, overnight, 94% (**30**), dimethylamine 2 M in THF, THF, 40 °C, overnight, 92% (**31**), *N*-methylcyclohexylamine, toluene, 60 °C, 5 h, 71% (**32**), dicyclohexylamine, toluene, reflux, overnight, 74% (**33**), dibenzylamine, toluene, reflux, overnight, 75% (**34**), 1-adamantylamine, toluene, reflux, 6 h, 72% (**35**), quinuclidine, toluene, reflux, 1 h, 95% (**12**), azetidine, *N*,*N*-dimethylformamide (DMF), rt, 3 h, 95% (**37**), pyrrolidine, THF, reflux, overnight, 95% (**38**), pyperidine, toluene, reflux, overnight, 91% (**39**), morpholine, toluene, reflux, overnight, 84% (**40**); pyridine, 50 °C, 3 h, 92% (**17**); (d) MeI, dichloromethane (DCM), 35 °C, overnight, 94% (**4**), THF, rt, overnight, 96% (**6**), DCM, reflux, overnight, 74% (**7**), 62% (**8**), THF, reflux, overnight, 65% (**10**), DCM, reflux, overnight, 73% (**11**), DCM, reflux, 3 h, 65% (**13**), DCM, reflux, overnight, 95% (**14**), 94% (**15**), 92% (**16**); (e) BnBr, THF, reflux, overnight, 72% (**9**); (f) EtI, THF, reflux, overnight, 86% (**5**); (g) pic-BH_3_, CH_2_O_aq._ 37%, MeOH/DCM, AcOH, rt, overnight, 80% (**36**).

Compounds **4**–**17** were synthesized according to Scheme 1. The commercially available building block (*E*)-4-hydroxystilbene was alkylated using dibromoethane under basic conditions affording the intermediate **28**. Upon conversion by Finkelstein reaction to the iodo-derivative **29**, treatment with a selected variety of secondary amines provided the tertiary amines **30**–**34** and **37**–**40**, which were quaternarized by treatment with either methyl or ethyl iodide or benzyl bromide to the corresponding quaternary ammonium iodide or bromide salts (**4**–**10**, **13**–**16**). Likewise, the reaction between the primary 1-adamantylamine and the intermediate **29** afforded the secondary amine **35**, which was methylated to the tertiary amine **36** by reductive amination, further methylated to **11** by treatment with methyl iodide. Similarly, quinuclidine and pyridine were coupled with **29** to obtain the quaternary ammonium iodide salts **12** and **17**.

**Scheme 2:**
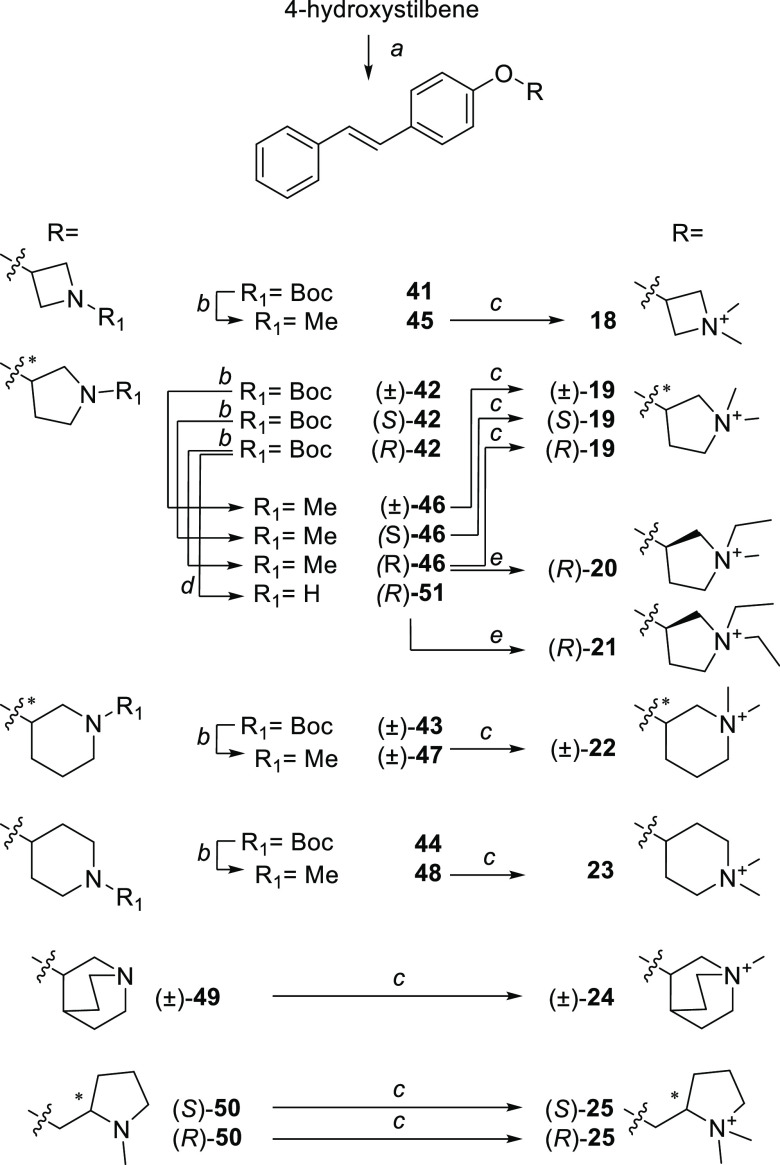
Reagents and Conditions (a) PPh_3_, diethyl azodicarboxylate (DEAD) or diisopropyl azodicarboxylate (DIAD), THF, reflux, overnight, *N*-boc-3-hydroxyazetidine, 50% (**41**), (±)-*N*-boc-3-hydroxypyrrolidine, 55% ((±)-**42**), (*R*)-*N*-boc-3-hydroxypyrrolidine, 49% ((*S*)-**42**), (*S*)-*N*-boc-3-hydroxypyrrolidine, 53% ((*R*)-**42**), (±)-*N*-boc-3-hydroxypyperidine, 39% ((±)-**43**), *N*-boc-4-hydroxypyperidine, 35% (**44**), (±)-3-quinuclidin-ol, 39% ((±)-**49**), (*S*)-*N*-methyl-2-pyrrolidinemethanol, room temperature, 60% ((*S*)-**50**) and (*R*)-*N*-methyl-2-pyrrolidinemethanol, room temperature, 43% ((*R*)-**50**); (b) LiAlH_4_, THF, room temperature, 24 h, 92% (**45**), 88% ((±)-**46**), 99% ((*S*)-**46**), 94% ((*R*)-**46**), 45% ((±)-**47**), and 39% (**48**); (c) MeI, DCM, reflux, 3 h, 27% (**18**); overnight, 72% ((±)-**19**), 98% ((*S*)-**19**), 99% ((*R*)-**19**), 83% ((±)-**22**), 89% (**23**), 56% ((±)-**24**), room temperature, 65% ((*S*)-**25**), and 59% ((*R*)-**25**); (d) HCl 1.25 M in MeOH/Et_2_O, rt, overnight, 96% ((*R*)-**51**); (e) EtI, DCE, rt, overnight, 99% ((*R*)-**20**), 53% ((*R*)-**21**).

Compounds **18**–**25** were synthesized according to Scheme 2. (*E*)-4-Hydroxystilbene was coupled by Mitsunobu reaction with the appropriate commercially available *N*-Boc-protected hydroxylated secondary cyclic amine, providing the intermediates **41**, (±)-**42**, (*S*)-**42**, (*R*)-**42**, (±)-**43**, and **44**, which were reduced by treatment with LiAlH_4_ to the corresponding *N*-methyl analogues **45**, (±)-**46**, (*S*)-**46**, (*R*)-**46**, (±)-**47**, and **48**. The same Mitsunobu conditions were applied to couple (*E*)-4-hydroxystilbene with the appropriate unprotected hydroxylated tertiary cyclic amines, providing intermediates (±)-**49**, (*S*)-**50**, and (*R*)-**50**, that together with **45**, (±)-**46**, (*S*)-**46**, (*R*)-**46**, (±)-**47**, and **48** were further methylated to the desired quaternary ammonium iodide salts by treatment with methyl iodide (compounds **18**, (±)-**19**, (*S*)-**19**, (*R*)-**19**, (±)-**22**, **23**, (±)-**24**, (*S*)-**25**, (*R*)-**25**) or with ethyl iodide ((*R*)-**20**). Additionally, (*R*)-**42** was also boc-deprotected to afford the secondary amine (*R*)-**51**, that was further ethylated to (*R*)-**21**.

**Scheme 3:**
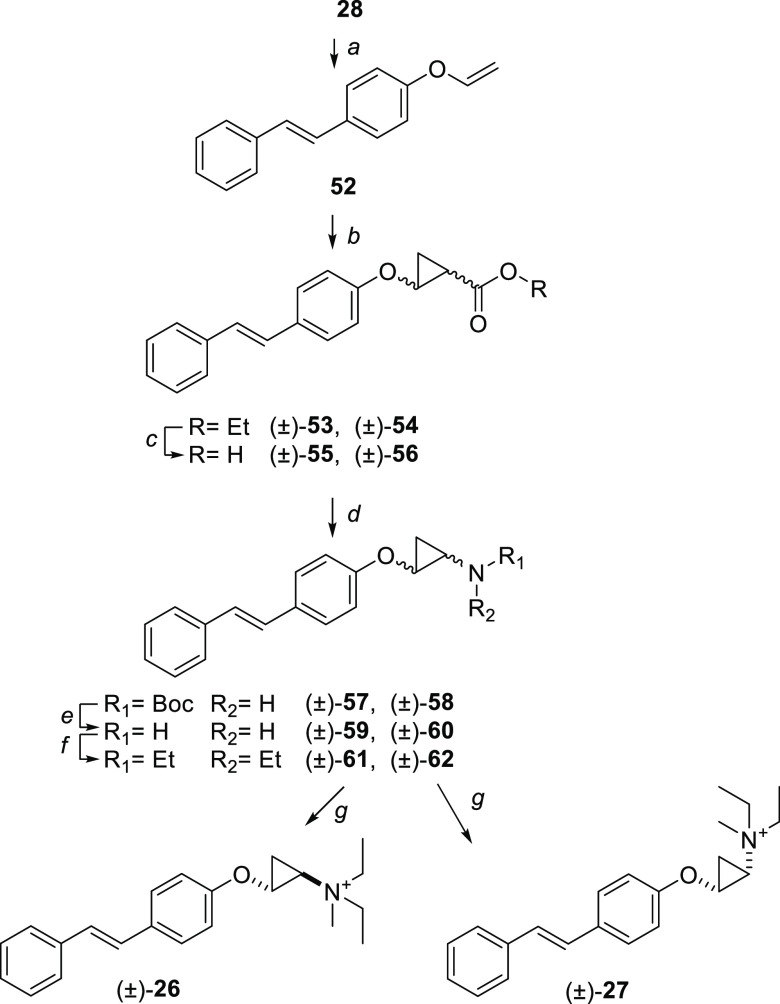
Reagents and Conditions (a) *t*-BuOK in THF, reflux, 4 h, 89% (**52**); (b) ethyl diazoacetate, Rh_2_(OAc)_4_, DCM, 0 °C, 2 h, 36% ((±)-**53**), 33% ((±)-**54**); (c) NaOH, EtOH, reflux, 3 h, 95% ((±)-**55**), 78% ((±)-**56**); (d) (1) *iso-*butyl chloroformate, triethylamine (TEA), Et_2_O, −10 °C, 2h, (2) NaN_3_, (*n*-Bu)_4_NBr, THF, 40 °C, 4 h, and (3) *t*-BuOH, reflux, 48 h, 91% ((±)-**57**), 65% ((±)-**58**); (e) HCl in MeOH, reflux, 3 h, 58% ((±)-**59**), 75% ((±)-**60**); (f) K_2_CO_3_, EtI, 40 °C, 20 h, 53% ((±)-**61**), 32% ((±)-**62**); (g) MeI, reflux, 4 h, 50% ((±)-**26**), overnight, 41% ((±)-**27**).

As shown in Scheme 3, the racemic cyclopropane-based analogues (±)-**26** and (±)-**27** were synthesized from the intermediate **28**, which was dehydrobrominated by treatment with *t*-BuOK at reflux to the olefin **52** and then cyclopropanated by treatment with ethyl diazoacetate and Rh_2_(OAc)_4_, providing the two racemates (±)-**53** and (±)-**54**. These were separated by flash column chromatography and further used individually. Upon ester hydrolysis in basic conditions to (±)-**55** and (±)-**56**, the resulting carboxylic acid functionalities were activated to mixed anhydrides by treatment with *iso-*butyl chloroformate, and then converted to the corresponding acyl azides with NaN_3_. Thermal decomposition in the presence of *tert*-butanol provided the *N*-Boc-protected intermediates (±)-**57** and (±)-**58**, by one-pot Curtius rearrangement and urethane formation. *N*-Boc deprotection in acidic conditions afforded the intermediates (±)-**59** and (±)-**60**, which were alkylated first to the corresponding tertiary amines (±)-**61** and (±)-**62** with ethyl iodide, and further methylated with methyl iodide to the desired racemic mixtures (±)-**26** and (±)-**27**.

### Biology

#### Binding Studies

We tested the affinity of all of the synthesized compounds for human α7-nAChR using competition binding assays. A wide selection of the synthesized compounds (primarily those with high affinity for α7-nAChR) were also assessed for competitive binding affinity at human α4β2- and α3β4-nAChR (Table 1). SH-EP1 cells stably transfected with α3β4-nAChR were those described in an earlier publication.^16^ HEK 293 cells stably transfected with α4β2-nAChR were a generous gift from Lindstrom,^17^ whereas the α7 subtype was transiently expressed in SH-SY5Y cells as previously described.^12^ Radiolabeling of α7 nAChRs was performed using [^125^I]-α-Bgtx at the saturating concentration of 2–3 nM, while for labeling of the two heteromeric subtypes, we used 0.1 nM [^3^H]-epibatidine for the α4β2 nAChR subtype and 0.25 nM [^3^H]-epibatidine for the α3β4 nAChR.

**Table 1:** Affinity (*K*_i_ in nM or μM) of Compounds for the Human α7, α3β4, and α4β2 nAChR Subtypes^a^

	α7 nAChR [^125^I]-αBgtx *K*_i_ (nM)	α3β4 nAChR [^3^H]-Epi *K*_i_ (nM)	α4β2 nAChR [^3^H]-Epi *K*_i_ (μM)		α7 nAChR [^125^I]-αBgtx *K*_i_ (nM)	α3β4 nAChR [^3^H]-Epi *K*_i_ (nM)	α4β2 nAChR [^3^H]-Epi *K*_i_ (μM)
**1**	104 (55–202)	433 (227–823)	5.7 (3–10.6)	**17**	5900 (500–9700)	nd	nd
**4**	34 (12–97)	158 (69–362)	nd	**18**	79 (68–107)	1580 (311–8000)	6.3 (1.3–2.8)
**5**	284 (88–916)	259 (141–476)	nd	(±)-**19**	59 (33–167)	576 (131–2500)	10.7 (3.3–34)
**6**	306 (112–838)	1527 (803–2900)	nd	(*S*)*-***19**	73 (33–165)	2800 (1900–4000)	2.3 (0.9–6)
**7**	2730 (605–12300)	3490 (1840–6620)	nd	(*R*)-**19**	23 (9–55)	2700 (1800–4200)	9.3 (2.9–30)
**8**	50000	nd	nd	(*R*)-**20**	222 (95–523)	875 (274–2790)	nd
**9**	3300 (900–12000)	794 (363–1739)	nd	(*R*)-**21**	635 (299–1349)	176 (81–384)	nd
**10**	10000	nd	nd	(±)-**22**	1123 (298–4232)	783 (556–1103)	nd
**11**	1600 (675–3780)	nd	nd	**23**	613 (125–2991)	368 (239–567)	3 (2.1–4.3)
**12**	37 (17–79)	33	1.9	(±)-**24**	248 (115–534)	95 (25–356)	1.9 (0.5–2.7)
**13**	112 (48–262)	662 (141–3100)	14.8 (6.2–35)	(*S*)-**25**	117 (66–208)	147 (66–325)	18.6 (5.9–58)
**14**	94 (38–229)	77 (14–414)	6.6 (2.8–15)	(*R*)-**25**	110 (51–243)	159 (96–264)	1.3 (0.8–2.4)
**15**	163 (108–247)	44 (20–264)	7.4 (3.2–17)	(±)-**26**	3900 (2070–7060)	546 (275–1800)	12 (2.1–73)
**16**	259 (123–545)	285 (55–1400)	11.3 (6.2–20)	(±)-**27**	567 (278–1156)	1270 (382–3800)	1.3 (0.8–1.8)

a Heterologously expressed α4β2 and α3β4 human receptors were expressed in HEK293 or SH-EP1 cells, respectively, whereas the human α 7 nAChR was expressed in SH-SY5Y human neuroblastoma cells. Binding was assessed with [^3^H]epibatidine for the α4β2 and α3β4 nAChR subtypes and [^125^I]α-bungarotoxin for the α7 subtype. The *K*_i_ values were derived from three [^3^H]-epibatidine and [^125^I]-α-bungarotoxin competition binding experiments for each compound on each subtype. Each compound was tested in three separate competition binding curves for each subtype, and the inhibition constant (*K*_i_) was estimated by reference to the *K*_d_ of the radioligand, according to the Cheng–Prusoff equation. The numbers in brackets represent the confidence interval of the determined value. The affinity of the radioactive ligands [^3^H]epibatidine and [^125^I]α-bungarotoxin were determined in parallel on the same cell membranes expressing the indicated subtype by saturation curves with the radioactive ligands in the absence or presence of an excess of cold ligand. Data from saturation and competition binding curves were evaluated by one-site competitive binding curve-fitting procedures using GraphPad Prism version 6 (GraphPad Software, Inc, CA).

Among the compounds **4**–**17**, namely those modified at the ammonium head, high or moderate α7-nAChR affinity, ranging within half an order of magnitude of the parent compound **1** (*K*_i_ = 104 nM), was observed for the analogues **4**–**6** and **12**–**16**. Among the compounds **18**–**27**, modified at the O–N linker, analogously high or moderate α7-nAChR affinities were determined for **18**, the two enantiomeric pairs (*S*)-**19**-(*R*)-**19** and (*S*)-**25**-(*R*)-**25**, and also for (*R*)-**20** and (±)-**24**.

Affinity at α3β4-nAChR was determined for all of the compounds with high or moderate α7-nAChR affinity (*K*_i_ < 1 μM), and also for three further compounds with low α7-nAChR affinity (**7**, (±)-**22**, and (±)*-***26**). Affinities at the α3β4- and α7-nAChR subtype were very similar or only moderately different (<10 ratio), except for **18** and the enantiomers (*S*)-**19** and (*R*)*-***19**, which showed high α7-nAChR affinity and also good α7- vs α3β4-nAChR selectivity.

On the other hand, affinity determination at α4β2-nAChR was limited to most of the compounds with high α7 affinity and, for completeness of SAR analysis, to the *trans* cyclopropane (±)-**26**. All of the tested compounds showed low α4β2 affinity (*K*_i_ > 1 μM).

#### *In Vitro* Functional Activity on α7 and α9α10-nAChR Subtypes

We have previously characterized **1** as a selective α7-nAChR antagonist with an IC_50_ of 109 nM at oocyte-expressed chick α7-nAChR. This value was determined by preapplication of increasing concentrations of **1** for 30 s, before the application of ACh (100 μM).^15^ Oocytes expressing α7-nAChRs did not show a detectable response to **1** applied alone at 10 nM–1 μM concentrations. Recently, we have reported that **1** reduces the ACh activation of both human α7- and α9α10-nAChRs expressed in oocytes with IC_50_ values of 41 and 10 nM, respectively.^12^ The experiments were performed applying 1-s pulses of 10 μM ACh (α9α10-nAChR) or 200 μM ACh (α7-nAChR) at regular time intervals to oocytes perfused or bath-applied with **1** at varying concentrations.^18^

Of the compounds reported in this manuscript, 12 (**6**, **7**, **12**–**15**, **18**, (*S*)-**19**, (*R*)-**19**, (±)-**22**, **23**, and (±)-**24**) were selected to test their ability to inhibit currents induced by co-applied 1 mM ACh in *Xenopus laevis* oocytes heterologously expressing human α7- and α9α10-nAChRs subtypes. The selection was done so as to include compounds representative of structural modifications both at the ammonium head (compounds **6**, **7**, **12**–**15**) and at the linker (compounds **18**, (*S*)-**19**, (*R*)-**19**, (±)-**22**, **23**, and (±)-**24**) and of both high (compounds **12**–**15**, **18**, (*S*)-**19**, (*R*)-**19**) and moderate or modest (compounds **6**, **7**, (±)-**22**, **23**, and (±)-**24**) α7 affinity. The equipment and technique used were essentially identical to those previously described for α7-nAChR expressed from unlinked subunits,^19^ except that α9α10-nAChRs were also expressed (also from unlinked subunit constructs, using a 9:1 ratio of α9 to α10 cRNAs). The magnitude of the expression of α9α10-nAChR function was found to be highly dependent on the ratio of subunit cRNAs injected into the oocytes, and the use of a 9:1 ratio of α9 to α10 cRNAs was chosen throughout this study because it produced the most function. A previous study indicates that these conditions likely produce α9α10-nAChR with a mix of both (α9)_2_(α10)_3_ and (α9)_3_(α10)_2_ stoichiometries.^20^

**Figure 1:**
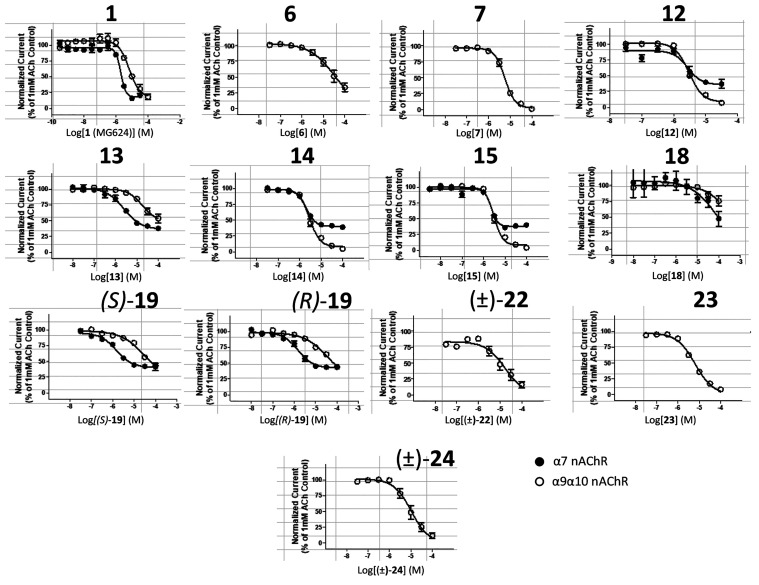
Antagonist concentration–response profiles for α7- or α9α10-nAChRs. Oocytes were injected with mRNA encoding human α7-nAChR subunits (together with NACHO to increase functional expression; ●), or human α9- and α10-nAChR subunits (9:1 ratio; ○). At 1 week after injection, the function of the corresponding nAChR populations was assessed using two-electrode voltage clamp electrophysiology. Before antagonists were applied to each oocyte, a train of five ACh stimulations was applied to ensure that stable function could be observed and to provide a positive control (1 s duration with 60 s wash periods between applications). Test compounds were co-applied with 1 mM ACh (1 s duration, 60 s wash periods between applications), starting at the lowest concentrations shown and increasing to 100 μM test compound in half-log increments. Magnitudes of responses in the presence of test compounds were normalized to the mean magnitude of the preceding positive control responses. Data points represent the mean ± standard error of the mean (S.E.M.) of 5–6 responses, recorded from individual oocytes—note that error bars are in some cases smaller than the associated points. For compounds **6**, **7**, (±)-**22**, **23**, and (±)-**24**, application at α7-nAChR had no effect on ACh-induced function; corresponding data are not shown for the sake of clarity.

The resulting IC_50_ values are reported in Table 2, together with those of the lead **1** for comparison. The corresponding concentration–response curves are shown in Figure 1. Eight of the tested compounds, namely, **1**, **12**, **13**, **14**, **15**, **18**, (*S*)-**19**, and (*R*)-**19**, inhibited ACh-induced currents at both the subtypes (although with higher potency at the α7-nAChR subtype), whereas the other five compounds **6**, **7**, (±)-**22**, **23**, and (±)-**24** had inhibitory effects on ACh-induced function at the α9α10-nAChR, but had no effect at the α7-subtype. Biphasic inhibition of α9α10-nAChR function was not shown by any of the compounds. This strongly suggests that none of these compounds distinguish between alternate α9α10-nAChR stoichiometries. As can be seen, the observed inhibition of ACh-induced function was not always complete due either to reaching a plateau of inhibition or to the use of test concentrations that were too low to obtain maximal inhibition. At the α9α10-nAChR, 100% inhibition was observed for compounds **7**, **12**, **14**, **15**, and **23** and it was almost complete for (±)-**22** and (±)-**24**, whereas it was near 80% for **1** and, notably, largely incomplete for **6**, **13**, **18**, (*S*)-**19**, and (*R*)-**19**, which are characterized by a smaller ammonium head or a more rigidified linker. On the other hand, 100% inhibition at the α7-nAChR was never observed.

**Table 2:** IC_50_ Values of α7 and α9α10 Inhibition Resulting from the Concentration–Response Curves Shown in Figure 1^a^

	oocyte-expressed α7 IC_50_ μM	oocyte-expressed α9α10 IC_50_ μM		oocyte-expressed α7 IC_50_ μM	oocyte-expressed α9α10 IC_50_ μM
**1**	1.99 (1.78–2.24)	6.68 (5.62–7.76)	**18**	78.2 (46.0–131.8)	346 (204–589)
**6**	NA	34.3 (28.2–40.74)	(*S*)-**19**	1.48 (1.23–1.78)	33.2 (16.6–66.1)
**7**	NA	5.74 (5.37–6.17)	(*R*)-**19**	1.49 (1.23–1.78)	36.5 (17.0–77.6)
**12**	2.75 (2.14–3.55)	3.01 (2.88–3.16)	(±)-**22**	NA	17.3 (13.8–21.9)
**13**	2.45 (2.02–3.02)	17.91 (12.88–24.55)	**23**	NA	6.31 (5.75–6.92)
**14**	2.04 (1.91–2.19)	2.85 (2.57–3.09)	(±)-**24**	NA	10.4 (8.91–12.3)
**15**	2.29 (2.04–2.57)	3.08 (2.88–3.31)			

a Summary of IC_50_ values of test compounds obtained from antagonist concentration–response profiles shown in Figure 1. Experimental details are provided in the caption of Figure 1 and in the Experimental Section. Values in parentheses represent the 95% confidence interval of the mean value. “NA” = not applicable, and denotes instances where no inhibition of agonist-induced function was observed, even in the presence of 100 μM test compound.

**Figure 2:**
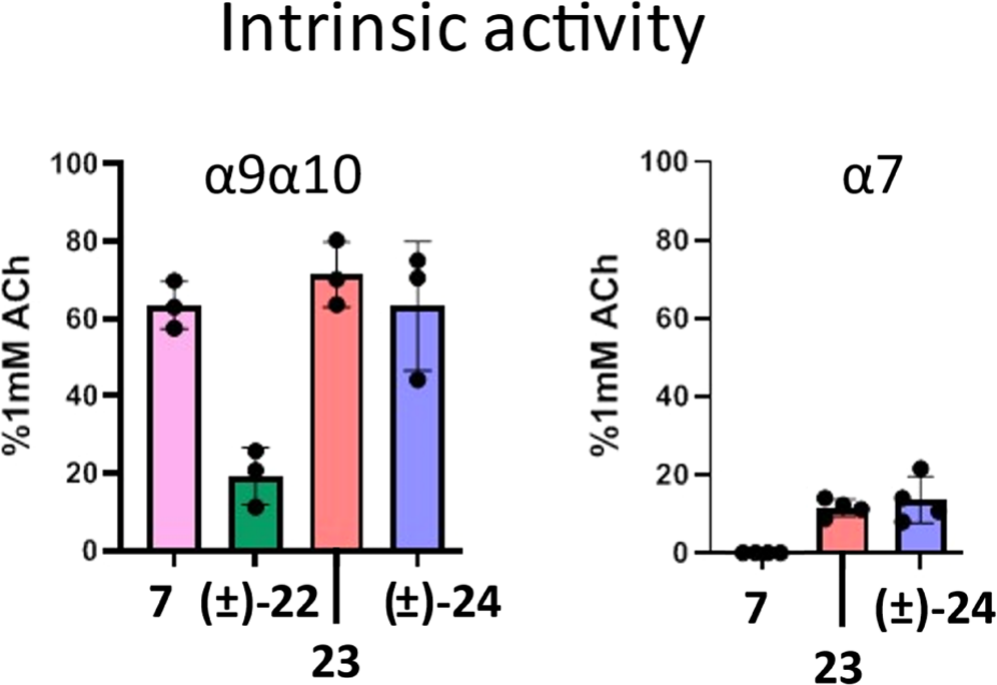
Intrinsic agonist activity of selected compounds at human α7- and α9α10-nAChRs. The ability of compounds of most interest (see text for details) to activate α9α10-nAChRs was assessed using two-electrode voltage clamp electrophysiology. As for antagonist concentration–response profiles, oocytes were first assessed using a set of five ACh-evoked applications (positive control; 1 s application, 60 s wash periods between applications). This was followed 60 s later by a 1 s application of the test compound at 100 μM (corresponding to the highest concentration used in the earlier antagonist concentration–response experiments). For each individual oocyte, the magnitude of the response evoked by the test compound was normalized to the mean of the magnitudes of the positive control responses. Data were collected from three individual oocytes, for each test compound, at each nAChR subtype and are presented as mean ± S.E.M. (histograms), with each individual response additionally shown as an individual point.

Compounds **7**, (±)-**22**, **23**, and (±)**24**, which showed no inhibition effect at the α7-nAChR and complete or nearly complete inhibition of ACh-induced responses at α9α10-nAChR, were tested for intrinsic agonist activity at the two nAChR subtypes. First, repeated control stimulations with a maximally effective dose of ACh (1 mM) were applied to establish the magnitude and stability of ACh control responses. Next, the test compounds were applied alone at 100 μM to oocytes expressing α9α10-nAChR (corresponding to the maximum concentration co-applied with ACh in Figure 1), or 10 μM to oocytes expressing α7-nAChR. When applied alone at a single, high, concentration the four compounds (**7**, (±)-**22**, **23**, and (±)**24**) behaved as partial agonists at α9α10-nAChRs (20–60% efficacy compared to ACh control) (Figure 2). In contrast, intrinsic agonist activity of compounds **7**, **23**, and (±)-**24** at α7-nAChRs was much lower (**23** and (±)-**24**) compared to that at the α9α10-nAChR or zero (**7**) (Figure 2). It is to be noted that **1** has been previously reported as a partial agonist at human α7-nAChR (40% of the response of 200 μM ACh) at high concentrations (100 μM).^10^

**Figure 3:**
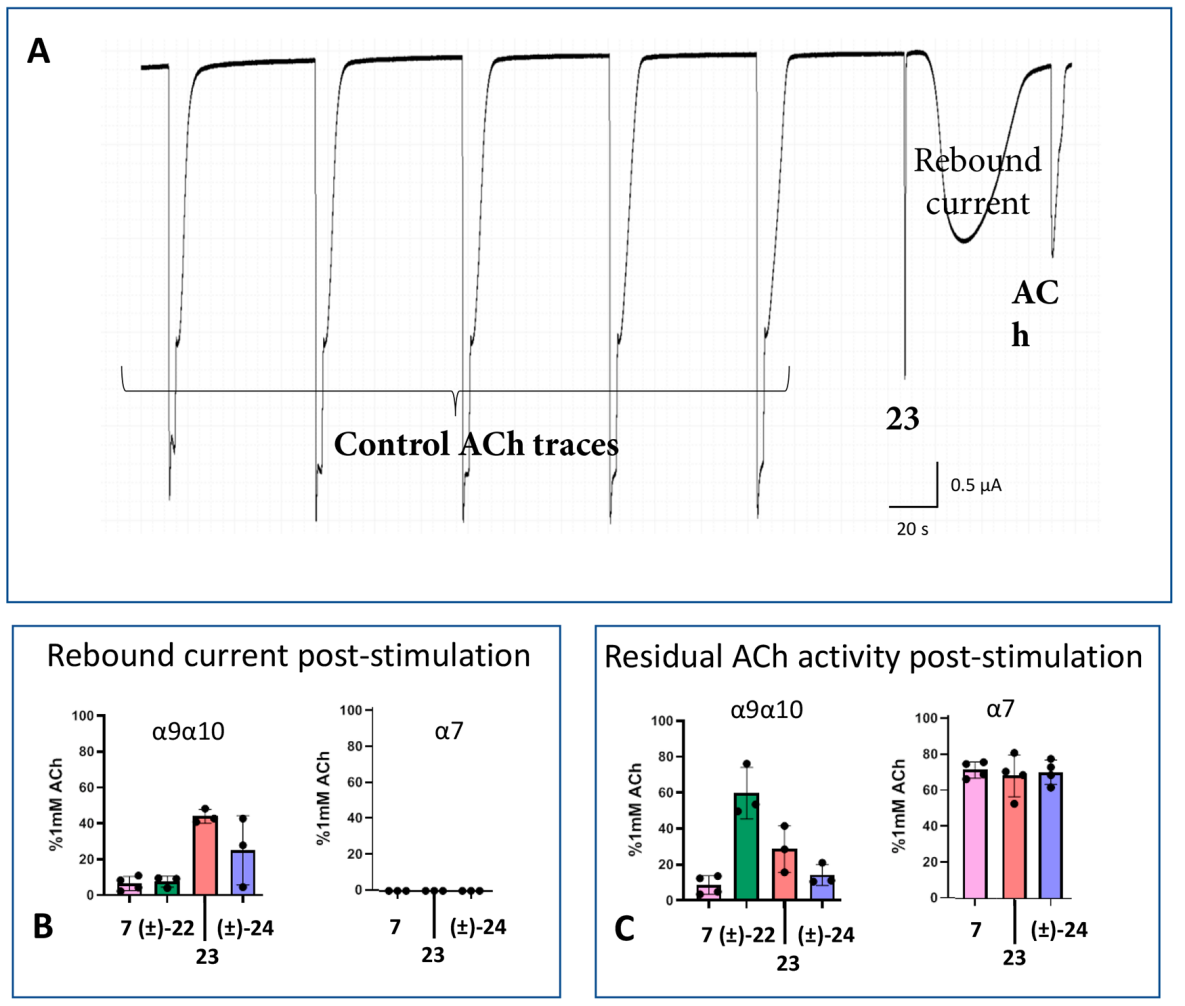
Assessment of rebound currents, residual ACh activity poststimulation with test compounds. (A) Example trace showing the characteristic truncated responses induced by all test compounds at α9α10-nAChR (summarized in Figure 2), followed by a “rebound” current that appears after test compound application is stopped, and diminished ACh response following application of the test compound. Compound **23** was chosen for this illustration since it produces the largest-magnitude rebound currents. (B) Magnitudes of rebound currents recorded from α9α10- and α7-nAChRs, normalized to the mean magnitude of the preceding control ACh traces (1 mM, 1 s application times). (C) Magnitudes of ACh control stimulation (1 mM, 1 s) applied 60 s following test compound applications. For (B) and (C), data were collected from three individual oocytes, for each test compound, at each nAChR subtype and are presented as mean ± S.E.M. (histograms), with individual responses in each case also shown as individual points.

Interestingly, responses induced by the test compounds at α9α10-nAChR were always shorter in duration than those induced by preceding ACh control applications. Further, responses to the test compounds were followed by rebound currents that were longer in duration than either the initial responses to the test compound, or the ACh control responses. In addition, responses to a further control application of ACh (following the application of the test compounds to each oocyte) produced a response much smaller in amplitude than the initial ACh control applications. An example trace depicting such behavior at α9α10-nAChR is shown in Figure 3A for compound **23**. In Figure 3B, the magnitude of the poststimulation rebound currents of **7**, (±)-**22**, (±)-**24**, and **23** at α9α10-nAChRs is represented, relative to that of the preceding ACh-induced control responses (note that no rebound currents were observed at α7-nAChR following application of **7**, (±)-**24**, and **23**). Figure 3C shows the residual activities induced by the final ACh (1 mM) control stimulation, following the test application of **7**, (±)-**22**, (±)-**24**, or **23** at α9α10-nAChRs, and of **7**, (±)-**24**, and **23** at α7-nAChRs. As can be seen, consistent with a profile of no efficacy at α7-nAChR, **7**, (±)-**24**, and **23** produced little block of subsequent ACh-induced function at this subtype, whereas the same compounds significantly blocked subsequent ACh-induced function at α9α10-nAChRs. Such a behavior is most evident for **7**, which induced a profound inhibition of the subsequent α9α10-nAChR response to ACh (1 mM) while showing essentially no effect on responses of α7-nAChRs (no intrinsic activity, no poststimulation rebound current, and very little decrease of ACh control response following application of **7**).

## Discussion

As in our previous investigation on onium-alkyloxy-stilbene-based compounds, the pharmacological characterization of this new series of stilbenol ammonium alkyl ethers, formally derived from the lead **1** by modification of the cationic head (**4**–**17**) or rigidification of the O–N linker (**18**–**27**), started from the evaluation of the α7-nAChR binding affinity by α-Bgtx displacement. This is indicative of competition for the same ACh orthosteric binding sites of this receptor and, in the development of our investigation, a useful guiding criterion in selecting candidates for functional study at both α7- and α9α10-nAChR.

As for cationic head modifications (compounds **4**–**17**), some SARs can be established by comparison with **1** (104 nM *K*_i_). The increase of steric bulk of the ammonium head by the gradual replacement of methyls with ethyls (in order, compounds **6**, **5**, **4**, and **1**) or by inclusion of two of the three alkyls in increasingly larger cycles (in order, compounds **13**, **14**, **15**, and **16**) enhances and then decreases the α7 affinity, reaching a maximum in the two series for **4** (34 nM *K*_i_) and **14** (94 nM *K*_i_). Compound **12**, with a quinuclidinium head, formally comparable to a diethylpropylammonium head, but downsized by conformational restrainment, has a high affinity (37 nM *K*_i_), similar to the diethylmethyl ammonium analogue **4** (34 nM *K*_i_). Conversely, cyclic substituent in place of one alkyl (compounds **7**, **9**, and **11**) is detrimental (1.6–3.3 μM *K*_i_) and replacement of two alkyls with cycles (compounds **8** and **10**) is not tolerated (>10 μM *K*_i_), as well as the radical change in shape and positive charge distribution resulting from replacement with a pyridinium head (compound **17**; 5.9 μM *K*_i_). As for the selectivity over the ganglionic α3β4-nAChR subtype, which is a critical issue in the development of ligands with high α7 affinity, the present cationic head modifications do not increase the α7- over α3β4-nAChR selectivity of the lead **1**. They maintain it or, in some cases, reduce or even invert it.

Modifications of the linker (compounds **18**–**27**) have also resulted in some compounds having similar or higher α7-nAChR affinity compared with **1**. This is the case of the compounds that maintain the two-carbon distance between oxygen and nitrogen enclosing, partially or totally, this fragment into a four or five-membered nitrogen heterocycle with two methyls quaternarizing the nitrogen atom (compounds **18**, **19**, and **25**). Notably, compared with **1**, compounds **18** (79 nM *K*_i_) and especially (*R*)-**19** (23 nM *K*_i_) display not only higher α7-nAChR affinity, but also a remarkably increased selectivity over the α3β4-nAChR subtype. This is in sharp contrast to what we observed for the two most ameliorative modifications, in terms of α7 affinity, of the cationic head which either did not increase (compound **4**) or nullified (compound **12**) the selectivity over the α3β4-nAChR subtype. On the other hand, as exemplified by the just mentioned **12**, **18**, **19**, and **25**, selectivity over the α4β2-nAChR subtype seems much less critical for these new stilbenol ammonium alkyl ethers as for those previously reported.^12^

As previously explained, *in vitro* functional activity at the α7 and α9α10-nAChRs was determined for **1** and 12 analogues, selected, among the initially tested twenty-seven, for structural representativeness and not *a priori* excluding compounds with moderate or modest α7 affinity. Given these selection criteria and the restricted number of compounds, a SAR analysis is arduous. Nevertheless, one cannot but notice two things. First, all 13 compounds antagonize ACh activity at α9α10-nAChR, but only some of them also at the α7-nAChR. Indeed, too small or too bulky cationic heads (**6** and **7**) and inclusion of the two-carbon linker into larger heterocycles than pyrrolidine (**22**, **23**, and **24**) lead to complete loss of α7-nAChR antagonism. Second, 100% inhibition of ACh-induced function at the α7-nAChR was never observed, whereas complete or nearly complete inhibition of the α9α10-nAChR to ACh was produced by most of the 13 tested compounds. Incomplete inhibition of the α7-ACh response could be due to partial agonism at high supra-micromolar concentrations of these compounds, as recently shown for **1**. This will be a matter for future investigation and discussion on analogues of **1** modified at the stilbene fragment, some of which resulted in selective and complete inhibition of ACh-induced function at the α7-nAChR. Here, we focused on compounds exhibiting only α9α10-nAChR antagonism and with complete or nearly complete inhibition of ACh response, namely, **7**, (±)-**22**, **23**, and (±)-**24**. The IC_50_ values of these four compounds range between 5 and 17 μM. We were interested in studying their behavior at both α7- and α9α10-nAChRs at high concentrations, those producing maximal inhibition of ACh responses (10 and 100 μM, respectively), and in the absence of ACh to better understand their mechanisms of action.

These further insights revealed common features of the above compounds, but with diversified profiles. Applied at high concentrations, all four of them have significant intrinsic activity at α9α10-nAChRs (20–60% efficacy compared to 1 mM ACh) and very low α7-nAChR activity, which becomes null in the case of **7** (which is also the compound with the lowest α7-nAChR binding affinity; 2730 nM *K*_i_). The very short duration of the α9α10-nAChR response, visible in the trace of **23** shown in Figure 3A, reflects an abrupt truncation of the induced passage of current through the channel most likely due to a rapid occupation and block of the open channel by the test compound after ligand-induced channel opening. Open-channel block would also explain the following occurrence of the rebound currents, which have a relatively long and variable duration due to gradual increase and subsequent slow baseline recovery. Once the compound engages the open channel, this would be held in an open position with no current passage, a mechanism demonstrated in a classic single-channel publication.^21^ Once compound application is stopped, the compound will start to wash out allowing current to flow again through the channel and then to slowly end when the channel closes by itself. Consistent with such an explanation, rebound currents were not observed at the α7-nAChR, which are not activated by these compounds.

A third consideration, consistent with those previously discussed concerning intrinsic activity and rebound current, can be advanced about residual ACh function, which was smaller in amplitude than the ACh control traces and after application of the test compounds alone (Figure 3C). Inspection of the traces of the compounds tested at α9α10-nAChR shows that the slower the recovery of the rebound current at the α9α10-nAChR, that is to say, the slower the disengagement of the test compounds from the channel, the smaller the residual ACh activity is. Indeed, plotting the amplitude of residual ACh-induced current, as a percent of ACh control, against the percentage recovery of the preapplication baseline just before the final ACh stimulation resulted in a good linear correlation. This would confirm that slow and incomplete recovery of the rebound current before the final ACh stimulation is applied results in extensive suppression of the final ACh peak. Again, compound **7** stands out for the lowest rebound current and the most extensive block of subsequent ACh-induced function at α9α10-nAChR and, on the other hand, for the minimum decrease of poststimulation residual ACh activity at α7-nAChR.

As for the selectivity against the α3β4 nAChR subtype, this is, in our experience, a crucial issue for the nicotinoids we have so far developed, namely, for the stilbenol ammonium ethyl ethers as α7-α9α10 ligands^12,15^ and for pyrrolidinyl-benzodioxanes as α4β2 ligands.^22,23^ Otherwise, the selectivity between these two latter subtypes is less challenging. The present results indicate that dissecting the α9α10-nAChR antagonist activity from the α7-nAChR antagonism can coincide with very low α7- and α3β4-nAChR affinities. This is the case, unique in the present series of stilbenol ammonium alkyl ethers, for compound **7**.

## Conclusions

A series of structural modifications restricting the flexibility of the ethylene linker or varying the bulk of the ammonium head of the 2-triethylammonium ethyl ether of 4-stilbenol (**1**), an α7 and α9α10-nAChR antagonist exhibiting partial agonism at high supra-micromolar concentrations, resulted in significant variations of its α7-nAChR affinity (104 nM *K*_i_). The modifications maintaining or increasing the α7-nAChR affinity of the lead did not substantially improve its profile in terms of potency as antagonist and/or of α7-/α9α10-nAChR selectivity (compounds **12**, **13**, **14**, **15**, **18**, and **19**). Otherwise, some modifications, detrimental for the α7-nAChR affinity, such as oversized increase or decrease of the ammonium head volume or inclusion of the linker in six-membered nitrogen heterocycles, resulted in α9α10-nAChR antagonists devoid of any antagonist activity at the α7-nAChR (compounds **6**, **7**, (±)-**22**, **23**, and (±)-**24**). Further characterization of these selective α9α10-nAChR antagonists for intrinsic activity at high concentrations at both the subtype receptors allowed us to get insight into the mechanism of their selective α9α10-nAChR antagonism, most likely consisting of opening and rapidly engaging the channel, then blocking it in an open and nonconducting state. Among these analogues of **1**, compound **7** stands out for its highest potency as an α9α10-nAChR antagonist and by exhibiting nearly complete block of poststimulation ACh activity at the α9α10-nAChR. The additional lack of an α7-nAChR response combined with very low α3β4-nAChR affinity makes compound **7** an invaluable and, to our knowledge, unique tool to define the highly debated potential of α9α10-nAChR antagonists as new therapeutics for the treatment of inflammatory and neuropathic pain.

## Experimental Section

### Chemistry

All chemicals and solvents were used as received from commercial sources or prepared as described in the literature. Flash chromatography purifications were performed using KP-Sil 32–63 μm 60 Å cartridges. Thin-layer chromatography (TLC) analyses were carried out on alumina sheets precoated with silica gel 60 F254 and visualized with UV light. Content of saturated aqueous solution of ammonia in eluent mixtures is given as v/v percentage. *R*_*f*_ values are given for guidance. ^1^H and ^13^C NMR spectra were recorded at 300 and 75 MHz using an FT-NMR spectrometer. Chemical shifts are reported in ppm relative to residual solvent (CHCl_3_, MeOH, or DMSO) as internal standard. Melting points were determined by Buchi Melting Point B-540 apparatus. Optical rotations were determined using a Jasco P-1010 polarimeter. Chiral high-performance liquid chromatography (HPLC) analyses were performed using Hewlett Packard 1050 instrument.

Liquid chromatography–mass spectrometry (LC-MS) analysis was performed using an Agilent 1200 series solvent delivery system equipped with an autoinjector coupled to a PDA and an Agilent 6400 series triple quadrupole electrospray ionization detector. Gradients of 5% aqueous MeCN + 0.1% HCO_2_H (solvent A), and 95% aqueous MeCN + 0.05% HCO_2_H (solvent B) were employed. Purity was measured by analytical HPLC on an UltiMate HPLC system (Thermo Scientific) consisting of an LPG- 3400A pump (1 mL/min), a WPS-3000SL autosampler, and a DAD-3000D diode array detector using a Gemini- NX C18 column (4.6 mm × 250 mm, 3 μm, 110 Å); gradient elution 0 to 100% B (MeCN/H_2_O/TFA, 90:10:0.1) in solvent A (H_2_O/TFA, 100:0.1) over 20 min. Data were analyzed using Chromeleon Software v. 6.80. Purity is ≥95% and retention times (*R*_t_) are reported.

#### METHOD A: General Procedure for the Preparation of Compounds **30**–**35**, **37**–**40**, **12**, and **17**

Unless specified otherwise, (*E*)-1-(2-iodoethoxy)-stilbene **29** (1 equiv, 0.57 mmol) was dissolved in a solution of the appropriate amine (10 equiv) in toluene (3 mL) and vigorously stirred and heated under nitrogen atmosphere for 1–24 h. The crude mixture was purified as specified providing the desired compounds as white solids in 71–95% yields.

#### METHOD B: General Procedure for the Preparation of Compounds **4**–**11**, **13**–**16**, and **18**–**20**, **22**–**27**

Intermediates **30**–**34**, **36**–**40**, **45**, (±)-**46**, (*S*)-**46**, (*R*)-**46**, (±)-**47**, **48**, (±)-**49**, (*S*)-**50**, (*R*)-**50**, (±)-**61**, and (±)-**62** (1 equiv, 0.5 mmol) were dissolved in the specified solvent (2.5 mL), and the appropriate alkyl iodide or bromide (15–50 equiv) was added dropwise. The reaction mixture was stirred for 3–12 h at the specified temperature. Unless stated otherwise, the mixture was cooled to room temperature, diethyl ether was added and the suspension was filtered. The solid was washed with diethyl ether and dried, providing the desired compounds **4–11**, **13**–**16** and **18**, (±)-**19**, (*S*)-**19**, (*R*)-**19**, (*R*)-**20**, (±)-**22**, **23**, (±)-**24**, (*S*)-**25**, (*R*)-**25**, (±)-**26**, and (±)**-27** in yields ranging from 27 to 96%.

#### METHOD C: General Procedure for the Preparation of Compounds **41**–**44**, **49**, and **50**

To an oven-dried three-neck round-bottom flask, under inert atmosphere, (*E*)-4-hydroxystilbene (0.51 mmol, 1 equiv) and the appropriate *N*-Boc-protected hydroxylated secondary cyclic amine or hydroxylated tertiary cyclic amine (1 equiv) were dissolved in dry THF (1 mL), and a solution of triphenylphosphine (1.2–1.5 equiv) in dry THF (1 mL) was added dropwise. The mixture was cooled to −10 °C and a solution of either DIAD or DEAD (1.2–1.4 equiv) in dry THF (2 mL) was added dropwise. Unless otherwise specified, the reaction mixture was vigorously stirred at reflux temperature overnight. Upon cooling to room temperature, the reaction mixture was diluted with diethyl ether and washed with water and brine. The organic layer was dried over anhydrous Na_2_SO_4_, filtered, and concentrated under reduced pressure. The crude was purified as specified, providing the desired compounds **41**, (±)-**42**, (*S*)-**42**, (*R*)-**42**, (±)-**43**, **44**, (±)-**49**, (*S*)-**50**, and (*R*)-**50** in variable yields (31–61%).

#### METHOD D: General Procedure for the Preparation of Compounds **45**–**48**

In an oven-dried three-neck round-bottom flask under inert atmosphere at −15 °C, LiAlH_4_ (4 equiv) was suspended in dry THF. Intermediates **41**, (±)-**42**, (*S*)-**42**, (*R*)-**42**, (±)-**43**, and **44** (1 equiv) were dissolved in THF and added dropwise, keeping the temperature at −15 °C. The reaction mixture was warmed to room temperature and stirred overnight at room temperature. Upon cooling at 0 °C, the excess of LiAlH_4_ was quenched HCl 1 M and washed with diethyl ether. Upon basification of the water phase to pH 12 by addition of NaOH 1 M, the water layer was extracted three times with EtOAc. The organic layer was dried over anhydrous Na_2_SO_4_, filtered, and the solvent was evaporated under reduced pressure providing the desired compounds **45**, (±)-**46**, (*S*)-**46**, (*R*)-**46**, (±)-**47**, and **48** in 39–99% yield with no further purification unless specified.

#### METHOD E: General Procedure for the Preparation of (±)-**55** and (±)-**56**

Intermediates (±)-**53** or (±)-**54** (1.53 mmol, 1 equiv) were dissolved in absolute ethanol and a solution of NaOH 1 M (5 equiv) was added. The reaction mixture was stirred at 60 °C for 3 h. Upon cooling to room temperature, the solvent was evaporated under reduced pressure, the resulting crude was diluted with water and acidified to pH 3 by dropwise addition of a solution of HCl 1 M solution, and the product was extracted with EtOAc. The organic layer was dried over anhydrous Na_2_SO_4_, filtrated, and evaporated under reduced pressure to afford the desired compound in very good yields (78–95%).

#### METHOD F: General Procedure for the Preparation of (±)-**57** and (±)-**58**

The reaction was carried out in three successive steps. (1) In an oven-dried round-bottom flask under N_2_ atmosphere, (±)-**55** or (±)-**56** (0.7 mmol, 1 equiv) was suspended in anhydrous Et_2_O (4 mL). Upon addition of triethylamine (1.1 equiv), the reaction mixture was cooled to −10 °C, and isobutyl chloroformate (1.1 equiv) was added dropwise. The suspension was stirred at −10 °C for 2 h. Upon completion, monitored by TLC, the solvent was evaporated under reduced pressure and the crude containing the correspondent isobutyl anhydride was utilized in the next step without any further purification. (2) Under inert atmosphere, the crude was dissolved in anhydrous THF (10 mL). After the addition of sodium azide (12 equiv) and of a catalytic amount of tetrabutyl ammonium bromide (0.1 equiv), the mixture was stirred at 40 °C for 4 h. Upon cooling to room temperature, the solvent was concentrated under reduced pressure and the resulting residue was (3) dissolved in *tert*-butylalcohol (10 mL) and refluxed for 48 h, after which the solvent was removed under reduced pressure providing a residue that was purified by silica gel chromatography providing the desired compounds (±)-**57** or (±)-**58** as white solids in 65–91% yields.

#### METHOD G: General Procedure for the Preparation of Compounds (±)-**59** and (±)-**60**

Intermediates (±)-**57** or (±)-**58** (0.1 mmol, 1 equiv) were dissolved in MeOH (2 mL), and the solution was cooled to 0 °C. Under vigorous stirring, a methanolic solution of HCl 1.25 M (6.25 equiv) was added dropwise. The reaction mixture was heated at reflux temperature for 3 h. Upon cooling at room temperature, the organic solvent was concentrated under reduced pressure providing a crude solid, that was suspended in EtOAc, stirred for 1 h, and filtered, providing the desired compounds (±)-**59** and (±)-**60** as white solids in 58–75% yields.

#### METHOD H: General Procedure for the Preparation of (±)-**61** and (±)-**62**

Intermediates (±)-**59** or (±)-**60** (0.38 mmol, 1 equiv) were dissolved in DCM and washed with a saturated solution of NaHCO_3_ to generate the corresponding free base. The organic layer was dried over anhydrous Na_2_SO_4_, filtered, and the solvent was removed under vacuum. The residue was dissolved in ethyl iodide (4 mL) and K_2_CO_3_ (1.5 equiv) was added. The reaction mixture was stirred at 40 °C for 20 h and then concentrated under reduced pressure. The residue was purified by silica gel flash column chromatography (gradient from cyclohexane to cyclohexane/EtOAc 8:2 + 0.5% *N*,*N*-diisopropylethylamine (DIPEA)) providing the desired compounds (±)-**61** and (±)-**62** as white solids in 32–53% yields.

##### (*E*)-4-(2-Bromoethoxy)-stilbene (**28**)

A suspension of anhydrous K_2_CO_3_ (5.28 g, 45.9 mmol, 2.5 equiv), (*E*)-4-hydroxystilbene (3.00 g, 15.3 mmol, 1.0 equiv), and KI (0.19 g, 1.15 mmol, 0.075 equiv) in 30 mL of 2-methylethylketone was stirred for 30 min and then 1,2-dibromoethane was added (5.6 mL, 12.15 g, 64.5 mmol, 4.2 equiv). The reaction mixture was refluxed under nitrogen atmosphere for 48 h. Upon cooling at room temperature, the inorganic salts were removed by filtration and the solvent was evaporated under reduced pressure. The residue was diluted with DCM and washed with an aqueous solution of NaOH 1 M. The organic layer was dried over anhydrous Na_2_SO_4_, filtered, and the solvent was evaporated under reduced pressure. The crude was recrystallized from EtOAc to yield the desired product as a white powder in 61% yield. *R*_*f*_ (cyclohexane/EtOAc 9:1) = 0.53. Mp = 130 °C. (coherent with the literature^24^) ^1^H NMR (300 MHz, CDCl_3_) δ 7.55–7.40 (m, 4H), 7.35 (t, *J* = 7.5 Hz, 2H), 7.25 (m, 1H), 7.07 (d, *J* = 16.3 Hz, 1H), 6.98 (d, *J* = 16.3 Hz, 1H), 6.91 (d, *J* = 8.8 Hz, 2H), 4.32 (t, *J* = 6.3 Hz, 2H), 3.65 (t, *J* = 6.3 Hz, 2H).

##### (*E*)-4-(2-Iodoethoxy)-stilbene (**29**)

(*E*)-4-(2-bromoethoxy)-stilbene **28** (1.50 g, 4.95 mmol) was dissolved in 30 mL of a saturated solution of NaI in acetone and the reaction mixture was refluxed overnight. Afterward, the solvent was evaporated under vacuum, and the residue was diluted with diethyl ether, washed with a 10% solution of Na_2_S_2_O_5_, and then washed with brine. The organic phase was dried over anhydrous Na_2_SO_4_, filtered, and evaporated under vacuum, affording the desired product as a white powder in quantitative yield. *R*_*f*_ (cyclohexane/EtOAc 9:1) = 0.61. Mp = 136 °C. ^1^H NMR (300 MHz, CDCl_3_) δ 7.54–7.41 (m, 4H), 7.35 (t, *J* = 7.4 Hz, 2H), 7.26–7.20 (m, 1H), 7.07 (d, *J* = 16.3 Hz, 1H), 6.98 (d, *J* = 16.3 Hz, 1H), 6.90 (d, *J* = 8.8 Hz, 2H), 4.28 (t, *J* = 6.9 Hz, 2H), 3.43 (t, *J* = 6.9 Hz, 2H).

##### (*E*)-4-(2-(*N*,*N*-Diethylamino)ethyloxy)stilbene (**30**)

Obtained from 186 mg (0.53 mmol, 1 equiv) of (*E*)-4-(2-iodoethoxy)-stilbene **29** and diethylamine (10 equiv) in THF (3 mL), at reflux temperature, overnight according to METHOD A. The crude was concentrated under reduced pressure, the residue was dissolved in AcOEt and extracted three times with HCl. The water layer was basified to pH 10 with NaOH and extracted three times with AcOEt. The organic layer was dried over anhydrous Na_2_SO_4_, filtered, and evaporated under vacuum, providing the desired compound **30** as a white solid in 94% yield. Mp = 73–75 °C (coherent with the literature^25^). ^1^H NMR (300 MHz, CDCl_3_) δ 7.53–7.42 (m, 4H), 7.35 (t, *J* = 7.6 Hz, 2H), 7.28–7.19 (m, 1H), 7.07 (d, *J* = 16.3 Hz, 1H), 6.97 (d, *J* = 16.3 Hz, 1H), 6.90 (d, *J* = 8.9 Hz, 2H), 4.09 (t, *J* = 6.3 Hz, 2H), 2.90 (t, *J* = 6.3 Hz, 2H), 2.67 (q, *J* = 7.1 Hz, 4H), 1.09 (t, *J* = 7.1 Hz, 6H).

##### (*E*)-4-(2-(*N*,*N*-Dimethylamino)ethyloxy)stilbene (**31**)

Obtained from 280 mg (0.80 mmol, 1 equiv) of (*E*)-4-(2-iodoethoxy)-stilbene **29** and a 2 M solution of dimethylamine in THF (10 equiv) in THF, at 40 °C, overnight, according to METHOD A. The crude was concentrated under reduced pressure, the residue was dissolved in AcOEt and extracted three times with HCl. The water layer was basified to pH 10 with NaOH and extracted three times with AcOEt. The organic layer was dried over anhydrous Na_2_SO_4_, filtered, and evaporated under vacuum, providing the desired compound **31** as a white solid in 92% yield. Mp = 103–105 °C (coherent with the literature^26^). *R*_*f*_ (cyclohexane/EtOAc 1:1 + 3% TEA) = 0.2. ^1^H NMR (300 MHz, CDCl_3_) δ 7.54–7.42 (m, 4H), 7.35 (t, *J* = 7.5 Hz, 2H), 7.29–7.20 (m, 1H), 7.07 (d, *J* = 16.4 Hz, 1H), 7.02–6.89 (m, 3H), 4.10 (t, *J* = 5.8 Hz, 2H), 2.75 (t, *J* = 5.8 Hz, 2H), 2.36 (s, 6H).

##### (*E*)-4-(2-(*N*,*N*-Cyclohexylmethyl)aminoethyloxy)stilbene (**32**)

Obtained from 300 mg (0.86 mmol, 1 equiv) of (*E*)-4-(2-iodoethoxy)-stilbene **29** and *N*-methylcyclohexylamine (freshly distilled under vacuum, 20 equiv) in toluene (3 mL), at 60 °C, in 5 h, according to METHOD A. The crude was concentrated under reduced pressure and purified by flash column chromatography (gradient from DCM to DCM/MeOH 95:5 + 1.5% NH_3 (aq. 30%)_). The desired compound **32** was obtained as a white solid in 71% yield. *R*_*f*_ (DCM/MeOH 99:1 + 0.5% NH_3 (aq. 30%)_) = 0.5. ^1^H NMR (300 MHz, CDCl_3_) δ 7.50–7.40 (m, 4H), 7.34 (t, *J* = 7.6 Hz, 2H), 7.25–7.19 (m, 1H), 7.06 (d, *J* = 16.3 Hz, 1H), 6.96 (d, *J* = 16.3 Hz, 1H), 6.90 (d, *J* = 8.7 Hz, 2H), 4.07 (t, *J* = 6.3 Hz, 2H), 2.88 (t, *J* = 6.3 Hz, 2H), 2.48–2.42 (m, 1H), 2.39 (s, 3H), 1.92–1.76 (m, 4H), 1.61 (m, 2H), 1.31–1.18 (m, 4H).

##### (*E*)-4-(2-(*N*,*N*-Dicyclohexyl)aminoethyloxy)stilbene Hydrochloride (**33**)

Obtained from 300 mg (0.86 mmol, 1 equiv) of (*E*)-4-(2-iodoethoxy)-stilbene **29** and dicyclohexylamine (10 equiv) in toluene (5 mL) at reflux temperature, overnight, according to METHOD A. The crude mixture was concentrated under reduced pressure, and the excess of dicyclohexylamine was distilled under vacuum. The solid was dissolved in DCM and 0.5 mL of HCl in diethyl ether (2 M) were added dropwise. The resulting suspension was filtered, and the solid was washed with DCM providing the hydrochloric salt of the desired compound **33** in 74% yield. ^1^H NMR (300 MHz, CDCl_3_) δ 11.57 (bs, 1H), 7.53–7.40 (m, 4H), 7.33 (t, *J* = 7.6 Hz, 2H), 7.22 (m, 1H), 7.05 (d, *J* = 16.4 Hz, 1H), 6.96 (d, *J* = 16.4 Hz, 1H), 6.90 (d, *J* = 8.7 Hz, 2H), 4.66 (t, *J* = 5.9 Hz, 2H), 3.52–3.27 (m, 4H), 2.51–2.38 (m, 2H), 2.25–2.13 (m, 2H), 2.04–1.85 (m, 4H), 1.77–1.50 (m, 6H), 1.46–1.07 (m, 6H).

##### (*E*)-4-(2-(*N*,*N*-Dibenzylamino)ethyloxy)stilbene (**34**)

Obtained from 375 mg (1.07 mmol, 1 equiv) of (*E*)-4-(2-iodoethoxy)-stilbene **29** and dibenzylamine (5 equiv) in toluene (5 mL), at reflux temperature, overnight, according to METHOD A. Upon cooling, the white precipitate was removed by filtration and discarded. The filtrate was diluted with MeOH, the resulting suspension was filtered, and the solid was washed with MeOH. The desired compound **34** was obtained as a white solid in 75% yield. *R*_*f*_ (cyclohexane/EtOAc 95:5) = 0.36. Mp = 100–101 °C. ^1^H NMR (300 MHz, DMSO-*d*_6_) δ 7.60–7.45 (m, 4H), 7.43–7.29 (m, 10H), 7.27–7.22 (m, 3H), 7.19 (d, *J* = 16.4 Hz, 1H), 7.07 (d, *J* = 16.4 Hz, 1H), 6.89 (d, *J* = 8.7 Hz, 2H), 4.09 (t, *J* = 6.0 Hz, 2H), 3.68 (s, 4H), 2.79 (t, *J* = 6.0 Hz, 2H).

##### (*E*)-4-(2-(*N*-Adamantanyl)aminoethyloxy)stilbene (**35**)

Obtained from 305 mg (0.87 mmol, 1 equiv) of (*E*)-4-(2-iodoethoxy)-stilbene **29** and adamantylamine (5 equiv) in toluene (5 mL), at reflux temperature, overnight, according to METHOD A. The crude mixture was concentrated under reduced pressure and the residue was purified by flash column chromatography (gradient from DCM to DCM/MeOH 9:1 + 0.5% NH_3 (aq. 30%)_). The desired compound **35** was obtained as a white solid in 72% yield. *R*_*f*_ (DCM/MeOH 95:5 + 0.5% NH_3 (aq. 30%)_) = 0.31. ^1^H NMR (300 MHz, CDCl_3_) δ 7.54–7.41 (m, 4H), 7.34 (t, *J* = 7.4 Hz, 2H), 7.26–7.18 (m, 1H), 7.06 (d, *J* = 16.4 Hz, 1H), 7.02–6.86 (m, 3H), 4.09 (t, *J* = 5.5 Hz, 2H), 3.00 (t, *J* = 5.5 Hz, 2H), 2.15–2.05 (m, 3H), 1.77–1.62 (m, 12H).

##### (*E*)-4-(2-(*N*-Methyl-*N*-adamantanyl)aminoethyloxy)stilbene (**36**)

(*E*)-4-(2-(*N*-adamantanyl)aminoethyloxy)stilbene **35** (93 mg, 0.25 mmol, 1 equiv) was suspended in 3 mL of MeOH and 1 mL of DCM and cooled to 0 °C. An aqueous solution of formaldehyde (37% w/w, 0.074 mL, 1 mmol, 4 equiv) was added dropwise, followed by the addition of 40 μL of glacial acetic acid. After stirring for 10 min at 0 °C, 2-methylpyridine borane complex was added (17 mg, 0.25 mmol, 1 equiv) and the mixture was stirred at room temperature overnight. The solvents were evaporated under reduced pressure, the residue was diluted with 1 mL of HCl 1 M, and the mixture was stirred for 1 h. The water phase was washed with Et_2_O twice, basified to pH 8 with a saturated solution of Na_2_CO_3_, and extracted with EtOAc three times. The organic phase was dried over anhydrous Na_2_SO_4_, filtered, and evaporated to dryness, providing the desired compound **36** as a white solid in 80% yield. ^1^H NMR (300 MHz, CDCl_3_) δ 7.52–7.43 (m, 4H), 7.35 (t, *J* = 7.4 Hz, 3H), 7.25–7.21 (m, 1H), 7.06 (d, *J* = 16.4 Hz, 1H), 7.02–6.89 (m, 3H), 4.95 (m, 1H), 4.49 (m, 1H), 3.85 (m, 1H), 3.35–3.09 (m, 1H), 2.85 (d, *J* = 4.7 Hz, 3H), 2.32 (m, 3H), 2.18 (m, 6H), 1.69 (m, 6H).

##### (*E*)-4-(2-Azetidinethyloxy)stilbene (**37**)

Obtained from 255 mg (0.72 mmol, 1 equiv) of (*E*)-4-(2-iodoethoxy)-stilbene **29** and azetidine (5 equiv) in DMF (3 mL), at rt for 3 h, according to METHOD A. The reaction mixture was diluted with Et_2_O and washed with water three times. The organic layer was dried over anhydrous Na_2_SO_4_, filtered, and evaporated under reduced pressure providing the desired compound **37** as a white solid in 95% yield. *R*_*f*_ (DCM/MeOH 95:5 + 0.5% NH_3 (aq. 30%)_) = 0.19. ^1^H NMR (300 MHz, CDCl_3_) δ 7.53–7.41 (m, 4H), 7.34 (t, *J* = 7.6 Hz, 2H), 7.25–7.19 (m, 1H), 7.06 (d, *J* = 16.3 Hz, 1H), 6.96 (d, *J* = 16.3 Hz, 1H), 6.89 (d, *J* = 8.7 Hz, 2H), 4.05 (t, *J* = 5.5 Hz, 2H), 3.54–3.42 (m, 2H), 2.98–2.89 (m, 2H), 2.26–2.13 (m, 2H), 0.94–0.82 (m, 2H).

##### (*E*)-4-(2-Pyrrolidinethyloxy)stilbene (**38**)

Obtained from 90 mg (0.25 mmol, 1 equiv) of (*E*)-4-(2-iodoethoxy)-stilbene **29** and pyrrolidine (15 equiv) in THF (1 mL) at reflux temperature overnight, according to METHOD A. Upon cooling, the reaction mixture was diluted with Et_2_O and washed with water three times. The organic layer was dried over anhydrous Na_2_SO_4_, filtered, and evaporated under reduced pressure providing the desired compound **38** as an off-white solid in 95% yield. *R*_*f*_ (DCM/MeOH 9:1 + 1.5% NH_3 (aq. 30%)_) = 0.38. Mp = 105–106 °C. ^1^H NMR (300 MHz, CDCl_3_) δ 7.53–7.41 (m, 4H), 7.34 (t, *J* = 7.5 Hz, 2H), 7.25–7.20 (m, 1H), 7.06 (d, *J* = 16.4 Hz, 1H), 6.97 (d, *J* = 16.4 Hz, 1H), 6.91 (d, *J* = 8.8 Hz, 2H), 4.22 (t, *J* = 5.7 Hz, 2H), 3.03 (t, *J* = 5.7 Hz, 2H), 2.90–2.68 (m, 4H), 1.99–1.77 (m, 4H).

##### (*E*)-4-(2-Piperidinethyloxy)stilbene (**39**)

Obtained from 78 mg (0.22 mmol, 1 equiv) of (*E*)-4-(2-iodoethoxy)-stilbene **29** and piperidine (15 equiv) in toluene at reflux temperature overnight, according to METHOD A. Upon cooling, the reaction mixture was diluted with diethyl ether and washed with water three times. The organic layer was dried over anhydrous Na_2_SO_4_, filtered, and evaporated under reduced pressure providing the desired compound **39** as a white solid in 91% yield. Mp = 91 °C (coherent with the literature^27^); *R*_*f*_ (DCM/MeOH 9:1 + 1.5% NH_3 (aq. 30%)_) = 0.44. ^1^H NMR (300 MHz, CDCl_3_) δ 7.54–7.41 (m, 4H), 7.35 (t, *J* = 7.6 Hz, 2H), 7.25–7.20 (m, 1H), 7.06 (d, *J* = 16.4 Hz, 1H), 6.98 (d, *J* = 16.4 Hz, 1H), 6.89 (d, *J* = 8.8 Hz, 2H), 4.60–4.46 (m, 2H), 3.69–3.50 (m, 2H), 3.43–3.23 (m, 2H), 2.91–2.70 (m, 2H), 2.38–2.07 (m, 2H), 2.02–1.73 (m, 4H).

##### (*E*)-4-(2-Morpholinethyloxy)stilbene Hydrochloride (**40**)

Obtained from 169 mg (0.48 mmol, 1 equiv) of (*E*)-4-(2-iodoethoxy)-stilbene **29** and morpholine (15 equiv) in toluene at reflux temperature overnight, according to METHOD A. Upon cooling, the reaction mixture was diluted with 5 mL of Et_2_O and washed with water three times. A methanolic solution of HCl 1.25 M (1 mL) was added dropwise to the organic layer and the resulting suspension was stirred for 15 min at room temperature and then filtered, affording the hydrochloride salt of the desired compound **40** (80 mg, 0.23 mmol) as a white solid in 81% yield. Mp = 212–214 °C; (coherent with the literature^27^); *R*_*f*_ (DCM/MeOH 8:2 + 0.5% NH_3 (aq. 30%)_) = 0.46. ^1^H NMR (300 MHz, DMSO-*d*_6_) δ 10.95 (bs, 1H), 7.62–7.52 (m, 4H), 7.36 (t, *J* = 7.5 Hz, 2H), 7.28–7.17 (m, 2H), 7.12 (d, *J* = 16.4 Hz, 1H), 7.03 (d, *J* = 8.4 Hz, 2H), 4.44 (t, *J* = 5.0 Hz, 2H), 4.04–3.91 (m, 2H), 3.80 (m, 2H), 3.62–3.44 (m, 4H), 3.32–3.09 (m, 2H).

##### (*E*)-4-(2-(*N*,*N*-Diethyl-*N*-methylammonium)ethyloxy)stilbene Iodide (**4**)

Obtained from (*E*)-4-(2-(*N*,*N*-diethylamino)ethyloxy)stilbene **30** (95 mg, 0.32 mmol, 1 equiv) and methyl iodide (30 equiv) in DCM overnight at 35 °C, according to METHOD B. The desired compound **4** was obtained as a white solid in 94% yield. Mp = 219–220 °C (coherent with the literature^28^); *R*_t_ (LC-MS) = 3.654 min; LC-MS (ESI): *m*/*z* calcd for C_21_H_28_NO [M]^+^ = 310.22, found 310.2; *R*_t_ (HPLC) = 13.39 min; ^1^H NMR (300 MHz, CDCl_3_) δ 7.53–7.41 (m, 4H), 7.34 (t, *J* = 7.4 Hz, 2H), 7.26–7.20 (m, 1H), 7.04 (d, *J* = 16.4 Hz, 1H), 7.00–6.89 (m, 3H), 4.57–4.47 (m, 2H), 4.18–4.07 (m, 2H), 3.69 (d, *J* = 7.3 Hz, 4H), 3.36 (s, 3H), 1.46 (d, *J* = 7.3 Hz, 6H). ^13^C NMR (75 MHz, CDCl_3_) δ 156.6, 137.4, 131.8, 128.8, 128.1, 127.7, 127.7, 127.6, 126.5, 114.9, 77.4, 62.3, 60.2, 58.2, 48.8, 8.7.

##### (*E*)-4-(2-(*N*,*N*-Dimehtyl-*N*-ethylammonium)ethyloxy)stilbene Iodide (**5**)

Obtained from (*E*)-4-(2-(*N*,*N*-dimethylamino)ethyloxy)stilbene **31** (70 mg, 0.26 mmol, 1 equiv) and ethyl iodide (12 equiv) in THF overnight at reflux temperature, according to METHOD B. The desired product **5** was obtained as a white solid in 86% yield. Mp = 247–249 °C (coherent with the literature^29^); *R*_t_ (LC-MS) = 3.646 min; LC-MS (ESI): *m*/*z* calcd for C_20_H_26_NO [M]^+^ = 282.20, found 282.2; *R*_t_ (HPLC)= 13.08 min; ^1^H NMR (300 MHz, DMSO-*d*_6_) δ 7.65–7.52 (m, 4H), 7.36 (t, *J* = 7.5 Hz, 2H), 7.28–7.18 (m, 2H), 7.12 (d, *J* = 16.5 Hz, 1H), 7.02 (d, *J* = 8.7 Hz, 2H), 4.47 (t, *J* = 4.7 Hz, 2H), 3.81–3.72 (m, 2H), 3.47 (q, *J* = 7.2 Hz, 2H), 3.12 (s, 6H), 1.29 (t, *J* = 7.2 Hz, 3H). ^13^C NMR (75 MHz, DMSO-*d*_6_) δ 157.0, 137.2, 130.5, 128.7, 127.8, 127.3, 126.6, 126.2, 115.0, 61.4, 59.7, 50.2, 8.0.

##### (*E*)-4-(2-(Trimehtylammonium)ethyloxy)stilbene Iodide (**6**)

Obtained from (*E*)-4-(2-(*N*,*N*-dimethylamino)ethyloxy)stilbene **31** (70 mg, 0.26 mmol, 1 equiv) and methyl iodide (12 equiv) in THF overnight at rt, according to METHOD B. The desired product **6** was obtained as a white solid in 96% yield. Mp = 284–286 °C (coherent with the literature^29^); *R*_t_ (LC-MS) = 3.572 min; LC-MS (ESI): *m*/*z* calcd for C_19_H_24_NO [M]^+^ = 282.19, found 282.2; *R*_t_ (HPLC) = 12.87 min; ^1^H NMR (300 MHz, DMSO-*d*_6_) δ 7.67–7.52 (m, 4H), 7.36 (t, *J* = 7.5 Hz, 2H), 7.29–7.17 (m, 2H), 7.13 (d, *J* = 16.5 Hz, 1H), 7.03 (d, *J* = 8.8 Hz, 2H), 4.49 (t, *J* = 4.7 Hz, 2H), 3.85–3.74 (m, 2H), 3.19 (s, 9H). ^13^C NMR (75 MHz, DMSO-*d*_6_) δ 157.0, 137.2, 130.5, 128.7, 127.8, 127.3, 126.6, 126.2, 115.0, 64.1, 61.7, 53.2.

##### (*E*)-4-(2-(*N*-Cyclohexyl-*N*,*N*-dimethyl)ammoniumethyloxy)stilbene Iodide (**7**)

Obtained from (*E*)-4-(2-(*N*,*N*-cyclohexylmethylamino)ethyloxy)stilbene **32** (100 mg, 0.25 mmol, 1 equiv) and methyl iodide (50 equiv) in DCM overnight at reflux temperature, according to METHOD B. The desired product **7** was obtained as a white solid in 74% yield. *R*_t_ (LC-MS) = 3.980 min; LC-MS (ESI): *m*/*z* calcd for C_24_H_32_NO [M]^+^ = 350.25, found 350.3; *R*_t_ (HPLC) = 14.32 min; ^1^H NMR (300 MHz, CDCl_3_) δ 7.51–7.42 (m, 4H), 7.37–7.30 (m, 2H), 7.26–7.18 (m, 1H), 7.03 (d, *J* = 16.3 Hz, 1H), 7.02–6.95 (m, 1H), 6.93 (d, *J* = 8.8 Hz, 2H), 4.59–4.49 (m, 2H), 4.26–4.15 (m, 2H), 3.79–3.64 (m, 1H), 3.36 (s, 6H), 2.40–2.26 (m, 2H), 2.01 (d, *J* = 12.4 Hz, 2H), 1.77–1.72 (m, 1H), 1.63–1.33 (m, 4H), 1.29–1.14 (m, 1H). ^13^C NMR (75 MHz, CDCl_3_) δ 156.6, 137.4, 131.7, 128.8, 128.1, 127.7, 127.6, 126.5, 114.9, 74.6, 62.6, 61.4, 49.7, 31.0, 26.8, 25.4, 24.8.

To assess drug-likeness, physicochemical properties parameters were calculated for **7** using SwissADME.^30^ No violations of Lipinski’s rules or its extensions (Verber, Ghose, and Egan rules) were detected (477.42 g/mol MW, 1 HBA, 0 HBD, 3.09 calculated consensus Log *P*_o/w_, 125.85 molar refractivity, 9.23 Å^2^ TPSA and 7 rotatable bonds). **7** is predicted to be poorly soluble in water (log *S* = −7.01 according to the ESOL model).

##### (*E*)-4-(2-(*N*,*N*-Dicyclohexyl-*N*-methyl)ammoniumethyloxy)stilbene Iodide (**8**)

A suspension of (*E*)-4-(2-(*N*,*N*-dicyclohexylamino)ethyloxy)stilbene hydrochloride **33** (150 mg, 0.34 mmol, 1 equiv) in DCM was washed with a 1 M solution of NaOH two times, and the organic layer was dried over anhydrous Na_2_SO_4_, filtered, and evaporated under reduced pressure to provide the corresponding free base. The residue was redissolved in DCM (3 mL) and reacted with methyl iodide (50 equiv) overnight at reflux temperature, according to METHOD B. The desired product **8** was obtained as a white solid in 62% yield. *R*_t_ (LC-MS) = 4.178 min; LC-MS (ESI): *m*/*z* calcd for C_29_H_40_NO [M]^+^ = 418.31, found 418.3; *R*_t_ (HPLC) = 15.73 min; ^1^H NMR (300 MHz, CDCl_3_) δ 7.54–7.39 (m, 4H), 7.34 (t, *J* = 7.7 Hz, 2H), 7.25 (d, *J* = 8.6 Hz, 1H), 7.10–6.96 (m, 2H), 6.93 (d, *J* = 8.6 Hz, 2H), 4.64–4.40 (m, 2H), 4.18–4.01 (m, 2H), 3.83–3.62 (m, 2H), 3.19 (s, 3H), 2.41–2.17 (m, 4H), 2.12–1.87 (m, 4H), 1.82–1.61 (m, 6H), 1.58–1.36 (m, 4H), 1.36–1.14 (m, 2H). ^13^C NMR (75 MHz, CDCl_3_) δ 156.6, 137.4, 131.7, 128.8, 128.1, 127.8, 127.7, 127.6, 126.5, 114.9, 73.0, 63.2, 57.3, 44.7, 27.8, 27.8, 26.1, 26.0, 25.0.

##### (*E*)-4-(2-(*N*,*N*-diethyl-*N*-benzylammonium)ethyloxy)stilbene Bromide (**9**)

Obtained from (*E*)-4-(2-(*N*,*N*-diethylamino)ethyloxy)stilbene **30** (100 mg, 0.33 mmol, 1 equiv) and benzyl bromide (5 equiv) in THF overnight at reflux temperature, according to METHOD B. The desired product **9** was obtained as a white solid in 72% yield. Mp = 195–196 °C (coherent with the literature^28^); *R*_t_ (LC-MS) = 4.027 min; LC-MS (ESI): *m*/*z* calcd for C_27_H_32_NO [M]^+^ = 386.25, found 386.2; *R*_t_ (HPLC) = 14.82 min; ^1^H NMR (300 MHz, CD_3_OD) δ 7.67–7.60 (m, 2H), 7.60–7.48 (m, 7H), 7.33 (t, *J* = 7.5 Hz, 2H), 7.22 (tt, *J* = 7.2, 1.3 Hz, 1H), 7.14 (d, *J* = 16.4 Hz, 1H), 7.10–7.01 (m, 3H), 4.69 (s, 2H), 4.56 (t, *J* = 4.7 Hz, 2H), 3.76–3.66 (m, 2H), 3.43 (q, *J* = 7.2 Hz, 4H), 1.51 (t, *J* = 7.2 Hz, 6H). ^13^C NMR (75 MHz, CD_3_OD) δ 158.5, 139.0, 134.0, 132.9, 132.0, 130.6, 129.7, 129.0, 128.9, 128.7, 128.4, 128.39, 127.37, 116.0, 63.2, 62.8, 57.5, 55.3, 8.5.

##### (*E*)-4-(2-(*N*,*N*-Dibenzyl,-*N*-Methylammonium)ethyloxy)stilbene Iodide (**10**)

Obtained from (*E*)-4-(2-(*N*,*N*-dibenzylamino)ethyloxy)stilbene **34** (213 mg, 0.38 mmol, 1 equiv) and methyl iodide (42 equiv) in THF overnight at reflux temperature, according to METHOD B. Upon cooling to room temperature, diethyl ether was added and the suspension was filtered. The solid was triturated in isopropanol and diisopropylether, the suspension was filtered, and the solid was washed with diethyl ether, providing the desired compound **10** as a white solid in 65% yield. Mp = 122–124 °C. *R*_t_ (LC-MS) = 4.288 min; LC-MS (ESI): *m*/*z* calcd for C_31_H_32_NO [M]^+^ = 434.25, found 434.2; *R*_t_ (HPLC) = 15.61 min; ^1^H NMR (300 MHz, DMSO-*d*_6_) δ 7.70–7.63 (m, 4H), 7.63–7.49 (m, 10H), 7.37 (t, *J* = 7.6 Hz, 2H), 7.29–7.18 (m, 2H), 7.13 (d, *J* = 16.5 Hz, 1H), 7.06 (d, *J* = 8.6 Hz, 2H), 4.83 (d, *J* = 12.7 Hz, 2H), 4.71–4.55 (m, 4H), 3.71–3.59 (m, 2H), 3.01 (s, 3H). ^13^C NMR (75 MHz, DMSO-*d*_6_) δ 157.1, 137.2, 133.4, 130.6, 130.4, 129.0, 128.7, 127.8, 127.8, 127.7, 127.3, 126.6, 126.2, 115.0, 65.6, 61.3, 59.4, 46.4.

##### (*E*)-4-(2-(*N*,*N*-Dimethyl-*N*-adamantanyl)amminiumethyloxy)stilbene Iodide (**11**)

Obtained from (*E*)-4-(2-(*N*,*N*-adamantylmethylamino)ethyloxy)stilbene **36** (100 mg, 0.26 mmol, 1 equiv) and methyl iodide (30 equiv) in DCM at reflux temperature overnight, according to METHOD B. Upon cooling to room temperature, diethyl ether was added and the suspension was filtered. The solid was triturated in diisopropylether/isopropanol, the suspension was filtered, and the solid was washed with diethyl ether, providing the desired compound **11** as a white solid in 73% yield. *R*_t_ (LC-MS) = 4.120 min; LC-MS (ESI): *m*/*z* calcd for C_28_H_36_NO [M]^+^ = 402.28, found 402.3; *R*_t_ (HPLC) = 14.97 min; ^1^H NMR (300 MHz, DMSO-*d*_6_) δ 7.64–7.50 (m, 4H), 7.35 (t, *J* = 7.5 Hz, 2H), 7.28–7.17 (m, 2H), 7.12 (d, *J* = 16.5 Hz, 1H), 7.03 (d, *J* = 8.7 Hz, 2H), 4.50 (t, *J* = 5.1 Hz, 2H), 3.67 (d, *J* = 5.1 Hz, 2H), 2.98 (s, 6H), 2.30–2.17 (m, 3H), 2.06 (d, *J* = 3.1 Hz, 6H), 1.68–1.58 (m, 6H). ^13^C NMR (75 MHz, DMSO-*d*_6_) δ 157.1, 137.3, 130.6, 128.7, 127.8, 127.8, 127.3, 126.6, 126.2, 115.0, 74.9, 62.1, 56.3, 44.3, 34.5, 33.6, 29.8.

##### (*E*)-4-(2-Quinuclidiniumethyloxy)stilbene Iodide (**12**)

Obtained from 200 mg (0.57 mmol, 1 equiv) of (*E*)-4-(2-iodoethoxy)-stilbene **29** and quinuclidine (1 equiv) in toluene (5 mL) at reflux temperature for 1 h, according to METHOD A. Upon cooling to room temperature, the suspension was filtered, and the desired compound **12** was obtained as a white solid in 96% yield. Mp = 272–274 °C; *R*_t_ (LC-MS) = 3.747 min; LC-MS (ESI): *m*/*z* calcd for C_23_H_28_NO [M]^+^ = 334.22 found 334.3; *R*_t_ (HPLC) = 13.70 min; ^1^H NMR (300 MHz, DMSO-*d*_6_) δ 7.65–7.51 (m, 4H), 7.37 (t, *J* = 7.5 Hz, 2H), 7.30–7.18 (m, 2H), 7.13 (d, *J* = 16.5 Hz, 1H), 7.02 (d, *J* = 8.7 Hz, 2H), 4.46 (t, *J* = 4.8 Hz, 2H), 3.62 (t, *J* = 4.8 Hz, 2H), 3.59–3.51 (m, 6H), 2.08 (p, *J* = 3.2 Hz, 1H), 1.88 (ddt, *J* = 8.5, 5.3, 3.0 Hz, 6H). ^13^C NMR (75 MHz, DMSO-*d*_6_) δ 157.0, 137.2, 130.5, 128.7, 127.8, 127.8, 127.3, 126.6, 126.2, 115.0, 62.3, 61.0, 54.5, 23.4, 19.0.

##### (*E*)-4-(2-(*N*-Methyl)azetidiniumethyloxy)stilbene Iodide (**13**)

Obtained from (*E*)-4-(2-azetidinethyloxy)stilbene **37** (185 mg, 0.66 mmol, 1 equiv) and methyl iodide (30 equiv) in DCM for 3 h at reflux temperature, according to METHOD B. The desired compound **13** was obtained as a white solid in 65% yield. *R*_t_ (LC-MS) = 3.658 min; LC-MS (ESI): *m*/*z* calcd for C_20_H_24_NO [M]^+^ = 294.19, found 294.2; *R*_t_ (HPLC) = 12.96 min; ^1^H NMR (300 MHz, DMSO-*d*_6_) δ 7.67–7.52 (m, 4H), 7.36 (t, *J* = 7.5 Hz, 2H), 7.29–7.18 (m, 2H), 7.13 (d, *J* = 16.5 Hz, 1H), 7.07–6.98 (m, 2H), 4.53 (q, *J* = 9.2 Hz, 2H), 4.40 (t, *J* = 4.9 Hz, 2H), 4.26–4.11 (m, 2H), 3.87 (t, *J* = 4.9 Hz, 2H), 3.23 (s, 3H), 2.76–2.57 (m, 1H), 2.48–2.31 (m, 1H). ^13^C NMR (75 MHz, DMSO-*d*_6_) δ 157.0, 137.2, 130.5, 128.6, 127.8, 127.2, 126.6, 126.2, 114.9, 65.2, 62.0, 61.1, 48.6, 13.9.

##### (*E*)-4-(2-(*N*-Methyl)pyrrolidiniumethyloxy)stilbene Iodide (**14**)

Obtained from (*E*)-4-(2-pyrrolidinethyloxy)stilbene **38** (50 mg, 0.17 mmol, 1 equiv) and methyl iodide (30 equiv) in DCM at reflux temperature overnight, according to METHOD B. The desired compound **14** was obtained as a white solid in 95% yield. Mp = 134–136 °C. *R*_t_ (LC-MS) = 3.711 min; LC-MS (ESI): *m*/*z* calcd for C_21_H_26_NO [M]^+^ = 308.20 found 308.2; *R*_t_ (HPLC) = 13.21 min; ^1^H NMR (300 MHz, DMSO-*d*_6_) δ 7.63–7.54 (m, 4H), 7.37 (t, *J* = 7.5 Hz, 2H), 7.29–7.18 (m, 2H), 7.13 (d, *J* = 16.5 Hz, 1H), 7.03 (d, *J* = 8.7 Hz, 2H), 4.49 (t, *J* = 4.9 Hz, 2H), 3.83 (t, *J* = 4.9 Hz, 2H), 3.67–3.51 (m, 4H), 3.11 (s, 3H), 2.19–2.09 (m, 4H). ^13^C NMR (75 MHz, DMSO-*d*_6_) δ 157.0, 137.2, 130.6, 128.7, 127.8, 127.3, 126.6, 126.2, 115.0, 64.3, 62.2, 61.7, 48.1, 20.9.

##### (*E*)-4-(2-(*N*-Methyl)piperidiniumethyloxy)stilbene Iodide (**15**)

Obtained from (*E*)-4-(2-pyperidinethyloxy)stilbene **39** (45 mg, 0.15 mmol, 1 equiv) and methyl iodide (30 equiv) in DCM at reflux temperature overnight, according to METHOD B. The desired compound **15** was obtained as a white solid in 94% yield. Mp = 231–233 °C (coherent with the literature^29^); *R*_t_ (LC-MS) = 3.717 min; LC-MS (ESI): *m*/*z* calcd for C_22_H_28_NO [M]^+^ = 322.22, found 322.2; *R*_t_ (HPLC) = 13.59 min; ^1^H NMR (300 MHz, DMSO-*d*_6_) δ 7.67–7.49 (m, 4H), 7.37 (t, *J* = 7.5 Hz, 2H), 7.30–7.17 (m, 2H), 7.12 (d, *J* = 16.5 Hz, 1H), 7.02 (d, *J* = 8.6 Hz, 2H), 4.50 (t, *J* = 4.8 Hz, 2H), 3.83 (t, *J* = 4.8 Hz, 2H), 3.51–3.40 (m, 4H), 3.15 (s, 3H), 1.92–1.75 (m, 4H), 1.65–1.47 (m, 2H). ^13^C NMR (75 MHz, DMSO-*d*_6_) δ 157.0, 137.2, 130.5, 128.7, 127.8, 127.3, 126.6, 126.2, 115.0, 61.4, 61.1, 60.9, 47.9, 20.5, 19.3.

##### (*E*)-4-(2-(*N*-Methyl)morpholiniumethyloxy)stilbene Iodide (**16**)

A suspension of (*E*)-4-(2-morpholinethyloxy)stilbene hydrochloride **40** (100 mg, 0.29 mmol, 1 equiv) in 2 mL of DCM was washed with a 1 M solution of NaOH two times, and the organic layer was dried over anhydrous Na_2_SO_4_, filtered, and evaporated under reduced pressure to provide the corresponding free base. The residue was redissolved in DCM (3 mL) and treated with methyl iodide (30 equiv) at reflux temperature overnight, according to METHOD B. The desired compound **16** was obtained as a white solid in 92% yield. Mp = 227–228 °C (coherent with the literature^29^); *R*_t_ (LC-MS) = 3.495 min; LC-MS (ESI): *m*/*z* calcd for C_21_H_26_NO_2_ [M]^+^ = 324.20, found 324.2; *R*_t_ (HPLC)= 12.82 min; ^1^H NMR (300 MHz, DMSO-*d*_6_) δ 7.66–7.50 (m, 4H), 7.37 (t, *J* = 7.5 Hz, 2H), 7.31–7.18 (m, 2H), 7.13 (d, *J* = 16.5 Hz, 1H), 7.03 (d, *J* = 8.4 Hz, 2H), 4.53 (t, *J* = 4.7 Hz, 2H), 3.98 (t, *J* = 4.7 Hz, 6H), 3.67–3.48 (m, 4H), 3.29 (s, 3H). ^13^C NMR (75 MHz, DMSO-*d*_6_) δ 157.0, 137.2, 130.6, 128.7, 127.8, 127.8, 127.3, 126.6, 126.2, 115.0, 62.4, 61.0, 59.8, 59.8, 47.3.

##### (*E*)-4-(2-Pyridiniumethyloxy)stilbene Iodide (**17**)

Obtained from 127 mg (0.36 mmol, 1 equiv) of (*E*)-4-(2-iodoethoxy)-stilbene **29** and neat pyridine (3 mL) at 50 °C for 3 h, according to METHOD A. Upon cooling to room temperature, the crude was diluted with diethyl ether and the resulting suspension was filtered, providing the desired product **17** as a pale pink solid in 92% yield. Mp = 233–234 °C; *R*_t_ (LC-MS) = 3.605 min; LC-MS (ESI): *m*/*z* calcd for C_21_H_20_NO [M]^+^ = 302.15 found 302.2; *R*_t_ (HPLC) = 13.09 min; ^1^H NMR (300 MHz, DMSO-*d*_6_) δ 9.16 (d, *J* = 6.7 Hz, 2H), 8.66 (tt, *J* = 7.8, 1.4 Hz, 1H), 8.21 (t, *J* = 7.8, 6.7 Hz, 2H), 7.59–7.50 (m, 4H), 7.36 (t, *J* = 7.5 Hz, 2H), 7.28–7.15 (m, 2H), 7.10 (d, *J* = 16.5 Hz, 1H), 6.94 (d, *J* = 8.8 Hz, 2H), 5.06 (t, *J* = 4.9 Hz, 2H), 4.55 (t, *J* = 4.9 Hz, 2H). ^13^C NMR (75 MHz, DMSO-*d*_6_) δ 157.1, 146.1, 145.4, 137.2, 130.6, 128.7, 127.9, 127.8, 127.7, 127.3, 126.6, 126.2, 114.9, 66.2, 60.0.

##### *tert*-Butyl (*E*)-3-(4-Stilbenoxy)azetidine-1-carboxylate (**41**)

Obtained from (*E*)-4-hydroxystilbene (200 mg, 1.02 mmol, 1 equiv), *N*-boc-3-hydroxyazetidine (1 equiv), PPh_3_ (1.2 equiv), and DIAD (1.2 equiv), according to METHOD C. The desired product **41** was obtained as a white solid in 50% yield after purification by silica gel flash column chromatography (gradient from cyclohexane to cyclohexane/EtOAc 7:3). *R*_*f*_ (cyclohexane/EtOAc 8:2) = 0.45. ^1^H NMR (300 MHz, CDCl_3_) δ 7.52–7.43 (m, 4H), 7.35 (t, *J* = 7.4 Hz, 2H), 7.25–7.18 (m, 1H), 7.06 (d, *J* = 16.3 Hz, 1H), 6.98 (d, *J* = 16.3 Hz, 1H), 6.74 (d, *J* = 8.8 Hz, 2H), 4.97–4.86 (m, 1H), 4.31 (dd, *J* = 9.5, 6.4 Hz, 2H), 4.02 (dd, *J* = 9.5, 4.0 Hz, 2H), 1.48–1.42 (m, 9H).

##### (±)-*tert*-Butyl (*E*)-3-(4-Stilbenoxy)pyrrolidine-1-carboxylate ((±)-**42**)

Obtained from (*E*)-4-hydroxystilbene (100 mg, 0.51 mmol, 1 equiv), (±)-*N*-boc-3-hydroxypyrrolidine (1 equiv), PPh_3_ (1.2 equiv), and DEAD (1.2 equiv), according to METHOD C. The desired product (±)-**42** was obtained as a white solid in 55% yield after purification by silica gel flash column chromatography (gradient from cyclohexane to (*i*Pr)_2_O). *R*_*f*_ (cyclohexane/(*i*Pr)_2_O 1:1) = 0.31. ^1^H NMR (300 MHz, CDCl_3_) δ 7.54–7.42 (m, 4H), 7.35 (t, *J* = 7.5 Hz, 2H), 7.26–7.19 (m, 1H), 7.07 (d, *J* = 16.3 Hz, 1H), 6.98 (d, *J* = 16.3 Hz, 1H), 6.86 (d, *J* = 8.5 Hz, 2H), 4.98–4.86 (m, 1H), 3.69–3.45 (m, 4H), 2.28–2.05 (m, 2H), 1.47 (s, 9H).

##### (*S*)-*tert*-Butyl (*E*)-3-(4-Stilbenoxy)pyrrolidine-1-carboxylate ((*S*)-**42**)

Obtained from (*E*)-4-hydroxystilbene (500 mg, 2.55 mmol, 1 equiv), (*R*)-*N*-boc-3-hydroxypyrrolidine (1 equiv), PPh_3_ (1.2 equiv), and DEAD (1.2 equiv), according to METHOD C. The desired product (*S*)-**42** was obtained as a white solid in 49% yield after purification by silica gel flash column chromatography (gradient from cyclohexane to (*i*Pr)_2_O). Mp = 150–151 °C. TLC and ^1^H NMR data as for (±)-**i15**. [α]_D_^25^ = +6.13 (*c* 0.5, CHCl_3_); 99.9% e.e. (Lux 3 μ; amylose-2; hexane/*i*PrOH 8:2; *F* = 1 mL/min; λ = 253 nM; *R*_tS_ = 4.45 min).

##### (*R*)-*tert*-Butyl (*E*)-3-(4-Stilbenoxy)pyrrolidine-1-carboxylate ((*R*)-**42**)

Obtained from (*E*)-4-hydroxystilbene (500 mg, 2.55 mmol, 1 equiv), (*S*)-*N*-boc-3-hydroxypyrrolidine (1 equiv), PPh_3_ (1.2 equiv), and DEAD (1.2 equiv), according to METHOD C. The desired product (*R*)-**42** was obtained as a white solid in 53% yield after purification by silica gel flash column chromatography (gradient from cyclohexane to (*i*Pr)_2_O). mp, TLC and ^1^H NMR data as for (*S*)-**i15**. [α]_D_^25^ = −6.09 (*c* 0.5, CHCl_3_); 99.9% e.e. (Lux 3 μ; amylose-2; hexane/*i*PrOH 8:2; F = 1 mL/min; λ = 253 nM; *R*_tR_ = 5.29 min).

##### (±)-*tert*-Butyl (*E*)-3-(4-Stilbenoxy)pyperidine-1-carboxylate ((±)-**43**)

Obtained from (*E*)-4-hydroxystilbene (200 mg, 1.02 mmol, 1 equiv), (±)-*N*-boc-3-hydroxypyperidine (1 equiv), PPh_3_ (1.2 equiv), and DEAD (1.2 equiv), according to METHOD C. The desired product (±)-**43** was obtained as a white solid in 39% yield after purification by silica gel flash column chromatography (gradient from cyclohexane to cyclohexane/EtOAc 7:3). Mp = 123.5–125.0 °C. *R*_*f*_ (cyclohexane/EtOAc 8:2) = 0.59. ^1^H NMR (300 MHz, CDCl_3_) δ 7.53–7.39 (m, 4H), 7.35 (t, *J* = 7.5 Hz, 2H), 7.26–7.20 (m, 1H), 7.06 (d, *J* = 15.8 Hz, 1H), 7.01–6.88 (m, 3H), 4.33–4.19 (m, 1H), 4.04–3.75 (m, 1H), 3.75–3.50 (m, 1H), 3.42–2.87 (m, 2H), 2.12–1.99 (m, 1H), 1.92–1.65 (m, 2H), 1.61–1.54 (m, 1H), 1.42 (s, 9H).

##### *tert*-Butyl (*E*)-4-(4-Stilbenoxy)pyperidine-1-carboxylate (**44**)

Obtained from (*E*)-4-hydroxystilbene (500 mg, 2.04 mmol, 1 equiv), *N*-boc-4-hydroxypyperidine (1 equiv), PPh_3_ (1.2 equiv), and DEAD (1.2 equiv), according to METHOD C. The desired product **44** was obtained as a white solid in 35% yield after purification by silica gel flash column chromatography (gradient from cyclohexane to cyclohexane/EtOAc 7:3). *R*_*f*_ (cyclohexane/EtOAc 8:2) = 0.57. ^1^H NMR (300 MHz, CDCl_3_) δ 7.54–7.38 (m, 4H), 7.34 (t, *J* = 7.6 Hz, 2H), 7.25–7.19 (m, 1H), 7.06 (d, *J* = 16.3 Hz, 1H), 7.01–6.80 (m, 3H), 4.55–4.43 (m, 1H), 3.70 (ddd, *J* = 12.8, 6.1, 2.5 Hz, 2H), 3.43–3.24 (m, 2H), 2.06–1.87 (m, 2H), 1.86–1.69 (m, 2H), 1.49–1.44 (m, 9H).

##### (±)-(*E*)-3-(4-Stilbenoxy)quinuclidine Hydrochloride ((±)-**49**)

Obtained from (*E*)-4-hydroxystilbene (200 mg, 1.02 mmol, 1 equiv), (±)-3-hydroxyquinuclidine (1 equiv), PPh_3_ (1.5 equiv), and DEAD (1.5 equiv), according to METHOD C. The crude was purified by silica gel flash column chromatography (gradient from DCM to DCM/MeOH 8:2 + 1.5% NH_3 (aq. 30%)_), providing a white solid that was redissolved in diethyl ether, and treated with a methanolic solution of HCl 1.25M. The suspension was filtered under vacuum, and the solid was washed with Et_2_O and dried providing the desired product (±)-**49** as a white solid in 39% yield. *R*_*f*_ (DCM/MeOH 9:1 + 1.5% NH_3 (aq. 30%)_) = 0.34. ^1^H NMR (300 MHz, CDCl_3_) δ 12.45 (s, 1H), 7.51–7.41 (m, 4H), 7.34 (tt, *J* = 7.2, 1.2 Hz, 2H), 7.26–7.20 (m, 1H), 7.04 (d, *J* = 16.4 Hz, 1H), 6.97 (d, *J* = 16.4 Hz, 1H), 6.84 (d, *J* = 8.9 Hz, 2H), 4.82–4.69 (m, 1H), 3.78 (dd, *J* = 14.3, 8.0 Hz, 1H), 3.49–3.17 (m, 5H), 2.54 (m, 1H), 2.44–2.26 (m, 1H), 2.08 (m, 2H), 1.81 (m, 1H).

##### (2*S*)-1-Methyl-2-((4-(*E*)-stilbenoxy)methyl)pyrrolidine Hydrochloride ((*S*)-**50**)

Obtained from (*E*)-4-hydroxystilbene (896 mg, 4.57 mmol, 1 equiv), (*S*)-1-methyl-2-hydroxymethylpyrrolidine (1 equiv), PPh_3_ (1.4 equiv), and DEAD (1.4 equiv), according to METHOD C, at room temperature, overnight. The crude was purified by silica gel flash column chromatography (gradient from DCM to DCM/MeOH 95/5), affording a solid that was treated with a 4.6 M solution of HCl in EtOH to provide the desired compound (*S*)-**50** as a hydrochloric salt in 60% yield. Mp = 226–230 °C; [α]_D_^27^ = −7.62 (*c* 3, CHCl_3_/MeOH 1/1); ^1^H NMR (300 MHz, DMSO-*d*_6_) δ 10.89 (s, 1H), 7.61–7.53 (m, 4H), 7.36 (t, *J* = 7.5 Hz, 2H), 7.28–7.17 (m, 2H), 7.12 (d, *J* = 16.5 Hz, 1H), 7.07–6.98 (m, 2H), 4.51–4.27 (m, 2H), 3.88–3.70 (m, 1H), 3.65–3.49 (m, 1H), 3.21–3.01 (m, 1H), 2.93 (s, 3H), 2.38–2.15 (m, 1H), 2.15–1.89 (m, 2H), 1.89–1.72 (m, 1H).

##### (2*R*)-1-Methyl-2-((4-(*E*)-stilbenoxy)methyl)pyrrolidine Hydrochloride ((*R*)-**50**)

Obtained from (*E*)-4-hydroxystilbene (400 mg, 2.04 mmol, 1 equiv), (*R*)-1-methyl-2-hydroxymethylpyrrolidine (1 equiv), PPh_3_ (1.4 equiv), and DEAD (1.4 equiv), according to METHOD C, at room temperature, overnight. The crude was purified by silica gel flash column chromatography (gradient from DCM to DCM/MeOH 95/5), affording a solid that was treated with a 4.6 M solution of HCl in EtOH to provide the desired compound (*R*)-**50** as a hydrochloric salt in 43% yield. mp and ^1^H NMR as for (*S*)-**50**. [α]_D_^27^ = +7.70 (*c* 3, CHCl_3_/MeOH 1/1).

##### (*E*)-4-(3-*N*-Methylazetidinyloxy)stilbene (**45**)

Obtained from a suspension of LiAlH_4_ (4 equiv) in THF (1.5 mL) and a solution of *tert*-butyl (*E*)-3-(4-stilbenoxy)azetidine-1-carboxylate **41** (150 mg, 0.42 mmol, 1 equiv) in THF (1.5 mL), according to METHOD D. The desired product **45** was obtained as a white solid in 92% yield. *R*_*f*_ (DCM/MeOH 9:1 + 0,5% NH_3 (aq. 30%)_) = 0.18. ^1^H NMR (300 MHz, CDCl_3_) δ 7.52–7.40 (m, 4H), 7.34 (t, *J* = 7.4 Hz, 2H), 7.26–7.20 (m, 1H), 7.06 (d, *J* = 16.4 Hz, 1H), 6.97 (d, *J* = 16.4 Hz, 1H), 6.76 (d, *J* = 8.7 Hz, 2H), 4.77 (p, *J* = 5.7 Hz, 1H), 3.91–3.79 (m, 2H), 3.19–3.08 (m, 2H), 2.42 (s, 3H).

##### (±)-(*E*)-4-(3-*N*-Methyl-pyrrolidinyloxy)stilbene ((±)-**46**)

Obtained from a suspension of LiAlH_4_ (4 equiv) in THF (1 mL) and a solution of (±)-*tert*-butyl (*E*)-3-(4-stilbenoxy)pyrrolidine-1-carboxylate (±)-**42** (100 mg, 0.27 mmol, 1 equiv) in THF (1 mL), according to METHOD D. The desired product (±)-**46** was obtained as a white solid in 88% yield. *R*_*f*_ (DCM/MeOH 9:1 + 1% NH_3 (aq. 30%)_) = 0.32. ^1^H NMR (300 MHz, CDCl_3_) δ 7.52–7.40 (m, 4H), 7.34 (t, *J* = 7.4 Hz, 2H), 7.25–7.19 (m, 1H), 7.06 (d, *J* = 16.4 Hz, 1H), 6.96 (d, *J* = 16.4 Hz, 1H), 6.84 (d, *J* = 8.9 Hz, 2H), 4.96–4.79 (m, 1H), 3.05–2.92 (m, 1H), 2.92–2.81 (m, 2H), 2.63 (m, 1H), 2.48 (s, 3H), 2.41–2.24 (m, 1H), 2.15–1.98 (m, 1H).

##### (*S*)-(*E*)-4-(3-*N*-Methyl-pyrrolidinyloxy)stilbene ((*S*)-**46**)

Obtained from a suspension of LiAlH_4_ (4 equiv) in THF (3 mL) and a solution of (*S*)-*tert*-butyl (*E*)-3-(4-stilbenoxy)pyrrolidine-1-carboxylate (*S*)-**42** (300 mg, 0.82 mmol, 1 equiv) in THF (3 mL), according to METHOD D. The desired product (*S*)-**46** was obtained as a white solid in 99% yield. Mp = 150–151 °C. TLC and NMR data as for (±)-**46**. [α]_D_^20^ = +21.06 (*c* 0.5, CHCl_3_).

##### (*R*)-(*E*)-4-(3-*N*-Methyl-pyrrolidinyloxy)stilbene ((*R*)-**46**)

Obtained from a suspension of LiAlH_4_ (4 equiv) in THF (3 mL) and a solution of (*R*)-*tert*-butyl (*E*)-3-(4-stilbenoxy)pyrrolidine-1-carboxylate (*R*)-**42** (300 mg, 0.82 mmol, 1 equiv) in THF (3 mL), according to METHOD D. The desired product (*R*)-**46** was obtained as a white solid in 94% yield. mp, TLC and ^1^H NMR data as for (*S*)-**46**. [α]_D_^20^ = −20.52 (*c* 0.5, CHCl_3_).

##### (±)-(*E*)-4-(3-*N*-Methyl-pyperidyloxy)stilbene ((±)-**47**)

Obtained from a suspension of LiAlH_4_ (4 equiv) in THF (1.5 mL) and a solution of (±)-*tert*-butyl (*E*)-3-(4-stilbenoxy)pyperidine-1-carboxylate (±)-**43** (140 mg, 0.37 mmol, 1 equiv) in THF (1.5 mL), according to METHOD D. The desired product (±)-**47** was obtained as a white solid in 45% yield. Mp = 54–55 °C. *R*_*f*_ (DCM/MeOH 9:1 + 1.5% NH_3 (aq. 30%)_) = 0.67. ^1^H NMR (300 MHz, CDCl_3_) δ 7.52–7.41 (m, 4H), 7.38–7.31 (m, 2H), 7.24–7.20 (m, 1H), 7.06 (d, *J* = 16.3 Hz, 1H), 7.01–6.90 (m, 3H), 4.50–4.36 (m, 1H), 3.07–2.88 (m, 1H), 2.74–2.55 (m, 1H), 2.34 (s, 3H), 2.26–2.14 (m, 2H), 2.09–1.93 (m, 1H), 1.93–1.77 (m, 1H), 1.72–1.40 (m, 2H).

##### (*E*)-4-(4-*N*-Methyl-pyperidyloxy)stilbene (**48**)

Obtained from a suspension of LiAlH_4_ (4 equiv) in THF (1.5 mL) and a solution of *tert*-butyl (*E*)-4-(4-stilbenoxy)pyperidine-1-carboxylate **44** (150 mg, 0.40 mmol, 1 equiv) in THF (1.5 mL), according to METHOD D. The desired product **48** was obtained as a white solid in 39% yield. ^1^H NMR (300 MHz, CDCl_3_) δ 7.53–7.42 (m, 4H), 7.34 (t, *J* = 7.6 Hz, 2H), 7.25–7.19 (m, 1H), 7.06 (d, *J* = 16.7 Hz, 1H), 7.00–6.85 (m, 3H), 4.45–4.30 (m, 1H), 2.81–2.64 (m, 2H), 2.48–2.37 (m, 1H), 2.35 (s, 3H), 2.33–2.29 (m, 1H), 2.10–1.98 (m, 2H), 1.96–1.85 (m, 2H).

##### (*E*)-4-(3-*N*,*N*-Dimethyl-azetidiniumoxy)stilbene Iodide (**18**)

Obtained from (*E*)-4-(3-(*N*-methylazetidinyloxy)stilbene **45** (92 mg, 0.35 mmol, 1 equiv) and methyl iodide (30 equiv) in DCM, at reflux temperature for 3 h, according to METHOD B. The desired compound **18** was obtained as a white solid in 27% yield. *R*_t_ (LC-MS) = 3.569 min; LC-MS (ESI): *m*/*z* calcd for C_19_H_22_NO [M]^+^ = 280.17, found 280.2; *R*_t_ (HPLC) = 12.60 min; ^1^H NMR (300 MHz, DMSO-*d*_6_) δ 7.64–7.54 (m, 4H), 7.37 (t, *J* = 7.6 Hz, 2H), 7.29–7.19 (m, 2H), 7.14 (d, *J* = 16.5 Hz, 1H), 6.91 (d, *J* = 8.6 Hz, 2H), 5.32–5.18 (m, 1H), 4.80 (dd, *J* = 12.2, 6.6 Hz, 2H), 4.48 (dd, *J* = 12.2, 4.8 Hz, 2H), 3.27 (s, 3H), 3.24 (s, 3H). ^13^C NMR (75 MHz, DMSO-*d*_6_) δ 155.4, 137.2, 131.2, 128.7, 128.1, 127.6, 127.4, 127.0, 126.3, 115.1, 71.2, 63.5, 53.9, 52.8.

##### (±)-(*E*)-4-(3-*N*,*N*-Dimethyl-pyrrolidiniumoxy)stilbene Iodide ((±)-**19**)

Obtained from (±)-(*E*)-4-(3-(*N*-methylpyrrolidinyloxy)stilbene (±)-**46** (63 mg, 0.20 mmol, 1 equiv) and methyl iodide (30 equiv) in DCM, at reflux temperature overnight, according to METHOD B. The desired compound (±)-**19** was obtained as a white solid in 72% yield. *R*_t_ (LC-MS) = 3.586 min; LC-MS (ESI): *m*/*z* calcd for C_20_H_24_NO [M]^+^ = 294.19, found 294.2; *R*_t_ (HPLC) = 12.92 min. ^1^H NMR (300 MHz, DMSO-*d*_6_) δ 7.67–7.51 (m, 4H), 7.37 (t, *J* = 7.5 Hz, 2H), 7.29–7.18 (m, 2H), 7.13 (d, *J* = 16.4 Hz, 1H), 6.99 (d, *J* = 8.4 Hz, 2H), 5.35–5.15 (m, 1H), 3.92 (dd, *J* = 13.2, 6.0 Hz, 1H), 3.86–3.74 (m, 2H), 3.70–3.55 (m, 1H), 3.26 (s, 3H), 3.21 (s, 3H), 2.91–2.69 (m, 1H), 2.38–2.20 (m, 1H). ^13^C NMR (75 MHz, DMSO-*d*_6_) δ 155.8, 137.2, 130.7, 128.7, 128.0, 127.7, 127.3, 126.8, 126.2, 115.7, 74.9, 69.3, 64.1, 52.6, 52.4, 30.0.

##### (*S*)-(*E*)-4-(3-*N*,*N*-Dimethyl-pyrrolidiniumoxy)stilbene Iodide ((*S*)-**19**)

Obtained from (*S*)-(*E*)-4-(3-(*N*-methylpyrrolidinyloxy)stilbene (*S*)-**46** (120 mg, 0.45 mmol, 1 equiv) and methyl iodide (45 equiv) in DCM, at reflux temperature overnight, according to METHOD B. The desired compound (*S*)-**19** was obtained as a white solid in 98% yield. LC-MS, HPLC, ^1^H NMR, and ^13^C NMR data as for (±)-**19**. [α]_D_^20^ = +15.95 (*c* 0.5, DMSO).

##### (*R*)-(*E*)-4-(3-*N*,*N*-Dimethyl-pyrrolidiniumoxy)stilbene Iodide ((*R*)-**19**)

Obtained from (*R*)-(*E*)-4-(3-(*N*-methylpyrrolidinyloxy)stilbene (*R*)-**46** (120 mg, 0.45 mmol, 1 equiv) and methyl iodide (45 equiv) in DCM, at reflux temperature overnight, according to METHOD B. The desired compound (*R*)-**19** was obtained as a white solid in 99% yield. LC-MS, HPLC, ^1^H NMR, and ^13^C NMR data as for (±)-**19**. [α]_D_^20^ = −16.43 (*c* 0.5, DMSO).

##### (±)-(*E*)-4-(3-*N*,*N*-Dimethyl-pyperidiniumoxy)stilbene Iodide ((±)-**22**)

Obtained from (±)-(*E*)-4-(3-(*N*-methylpyperidinyloxy)stilbene (±)-**47** (47 mg, 0.16 mmol, 1 equiv) and methyl iodide (45 equiv) in DCM, at reflux temperature overnight, according to METHOD B. The desired compound (±)-**22** was obtained as a white solid in 83% yield. Mp = 290–292 °C. *R*_t_ (LC-MS) = 3.573 min; LC-MS (ESI): *m*/*z* calcd for C_21_H_26_NO [M]^+^ = 308.20, found 308.2; *R*_t_ (HPLC) = 13.27 min; ^1^H NMR (300 MHz, DMSO) δ 7.63–7.53 (m, 4H), 7.37 (t, *J* = 7.6 Hz, 2H), 7.29–7.18 (m, 2H), 7.13 (d, *J* = 16.5 Hz, 1H), 7.07 (d, *J* = 8.7 Hz, 2H), 5.06–4.91 (m, 1H), 3.67 (dd, *J* = 13.0, 3.6 Hz, 1H), 3.53 (dd, *J* = 13.0, 6.2 Hz, 1H), 3.49–3.39 (m, 2H), 3.22 (s, 3H), 3.17 (s, 3H), 2.12–1.84 (m, 3H), 1.84–1.66 (m, 1H). ^13^C NMR (75 MHz, DMSO-*d*_6_) δ 155.7, 137.2, 130.8, 128.7, 128.0, 127.8, 127.4, 126.7, 126.2, 116.1, 68.1, 61.8, 61.1, 52.9, 52.4, 25.9, 16.7.

##### (*E*)-4-(4-*N*,*N*-Dimethyl-pyperidiniumoxy)stilbene Iodide (**23**)

Obtained from (*E*)-4-(4-(*N*-methylpyperidinyloxy)stilbene **48** (41 mg, 0.14 mmol, 1 equiv) and methyl iodide (45 equiv) in DCM, at reflux temperature overnight, according to METHOD B. The desired compound **23** was obtained as a white solid in 89% yield. *R*_t_ (LC-MS) = 3.560 min; LC-MS (ESI): *m*/*z* calcd for C_21_H_26_NO [M]^+^ = 308.20, found 308.3; *R*_t_ (HPLC) = 13.15 min; ^1^H NMR (300 MHz, DMSO-*d*_6_) δ 7.62–7.53 (m, 4H), 7.36 (t, *J* = 7.5 Hz, 2H), 7.28–7.16 (m, 2H), 7.11 (d, *J* = 16.5 Hz, 1H), 7.05 (d, *J* = 8.3 Hz, 2H), 4.72–4.59 (m, 1H), 3.61–3.39 (m, 4H), 3.19 (s, 3H), 3.14 (s, 3H), 2.32–2.16 (m, 2H), 2.13–1.93 (m, 2H). ^13^C NMR (75 MHz, DMSO-*d*_6_) δ 156.0, 137.2, 130.5, 128.6, 127.9, 127.8, 127.2, 126.5, 126.2, 116.4, 67.7, 58.1, 51.8, 49.9, 24.5.

##### (±)-(*E*)-4-(3-*N*-Methyl-quinuclidiniumoxy)stilbene Iodide ((±)-**24**)

A suspension of (*E*)-3-(4-stilbenoxy)quinuclidine hydrochloride (±)-**49** (120 mg, 0.35 mmol, 1 equiv) in 3 mL of DCM was washed with a 1 M solution of NaOH two times, and the organic layer was dried over anhydrous Na_2_SO_4_, filtered, and evaporated under reduced pressure to provide the corresponding free base. The residue was redissolved in DCM (3 mL) and treated with methyl iodide (50 equiv) at reflux temperature overnight, according to METHOD B. The desired product (±)-**24** was obtained as a white solid in 56% yield. *R*_t_ (LC-MS) = 3.667 min; LC-MS (ESI): *m*/*z* calcd for C_22_H_26_NO [M]^+^ = 320.20, found 320.2; *R*_t_ (HPLC) = 13.39 min; ^1^H NMR (300 MHz, DMSO) δ 7.63–7.53 (m, 4H), 7.37 (t, *J* = 7.5 Hz, 2H), 7.30–7.18 (m, 2H), 7.13 (d, *J* = 16.5 Hz, 1H), 7.00 (d, *J* = 8.7 Hz, 2H), 5.04–4.88 (m, 1H), 3.96 (dd, *J* = 13.5, 8.1 Hz, 1H), 3.58–3.24 (m, 5H), 3.01 (s, 3H), 2.43 (p, *J* = 2.8 Hz, 1H), 2.25–1.76 (m, 4H). ^13^C NMR (75 MHz, DMSO-*d*_6_) δ 155.7, 137.2, 130.7, 128.6, 127.9, 127.7, 127.3, 126.7, 126.2, 115.8, 69.7, 61.8, 55.8, 55.2, 51.0, 23.3, 20.7, 17.6.

##### (*S*)-(*E*)-1-Methyl-2-(4-stilbenoxymethyl)pyrrolidinium Iodide ((*S*)-**25**)

A suspension of (2*S*)-1-methyl-2-((4-(*E*)-stilbenoxy)methyl)pyrrolidine hydrochloride (*S*)-**50** (225 mg, 0.68 mmol, 1 equiv) in 3 mL of DCM was washed with a 1 M solution of NaOH two times, and the organic layer was dried over anhydrous Na_2_SO_4_, filtered, and evaporated under reduced pressure to provide the corresponding free base. The residue was redissolved in DCM (3 mL) and reacted with methyl iodide (50 equiv) at room temperature overnight, according to METHOD B. The desired product (*S*)-**25** was obtained as a white solid in 65% yield. Mp = 245–248 °C (dec). *R*_t_ (LC-MS) = 3.637 min; LC-MS (ESI): *m*/*z* calcd for C_21_H_26_NO [M]^+^ = 308.20, found 308.2; *R*_t_ (HPLC) = 13.24 min; [α]_D_^25^ = −5.37 (*c* 2, DMSO). ^1^H NMR (300 MHz, DMSO-*d*_6_) δ 7.59 (dd, *J* = 8.7, 6.8 Hz, 4H), 7.37 (t, *J* = 7.6 Hz, 2H), 7.29–7.19 (m, 2H), 7.14 (d, *J* = 16.5 Hz, 1H), 7.06 (d, *J* = 8.7 Hz, 2H), 4.52–4.32 (m, 2H), 4.18–4.04 (m, 1H), 3.77–3.54 (m, 2H), 3.29 (s, 3H), 3.02 (s, 3H), 2.40–2.24 (m, 1H), 2.19–1.94 (m, 3H). ^13^C NMR (75 MHz, DMSO-*d*_6_) δ 156.9, 137.2, 130.6, 128.6, 127.79, 127.75, 127.3, 126.6, 126.2, 115.0, 72.7, 66.9, 64.3, 52.4, 45.2, 24.1, 19.4.

##### (*R*)-(*E*)-1-Methyl-2-(4-stilbenoxymethyl)pyrrolidinium Iodide ((*R*)-**25**)

A suspension of (2*R*)-1-methyl-2-((4-(*E*)-stilbenoxy)methyl)pyrrolidine hydrochloride (*R*)-**50** (700 mg, 2.12 mmol, 1 equiv) in 10 mL of DCM was washed with a 1 M solution of NaOH two times, and the organic layer was dried over anhydrous Na_2_SO_4_, filtered, and evaporated under reduced pressure to provide the corresponding free base. The residue was redissolved in DCM (3 mL) and reacted with methyl iodide (50 equiv) at room temperature overnight, according to METHOD B. The desired product (*R*)-**25** was obtained as a white solid in 59% yield. mp, LC-MS, HPLC, ^1^H NMR, and ^13^C NMR data as for (*S*)-**25**; [α]_D_^25^ = +5.40 (*c* 2, DMSO).

##### (*R*)-(*E*)-3-(4-Stilbenoxy)pyrrolidine ((*R*)-**51**)

To a solution of (*R*)-*tert*-butyl (*E*)-3-(4-stilbenoxy)pyrrolidine-1-carboxylate (*R*)-**42** (250 mg, 0.68 mmol, 1 equiv) in MeOH (3 mL) and Et_2_O (7 mL), a methanolic solution of HCl 1.25 M was added dropwise. The mixture was stirred at room temperature overnight, and the solvents were evaporated under reduced pressure. The residue was dissolved in HCl 1 M and washed with Et_2_O twice. The water layer was basified to pH 12 with NaOH 1 M and extracted three times with EtOAc. The organic phase was dried over anhydrous Na_2_SO_4_, filtered, and the solvent was evaporated under reduced pressure. The desired compound (*R*)-**51** was obtained as a white solid in 96% yield. Mp = 143–144 °C; *R*_*f*_ (DCM/MeOH 9/1 + 1% NH_3 (aq. 30%)_)= 0.29; [α]_D_^25^ = −1.84 (*c* 0.5, MeOH). ^1^H NMR (300 MHz, DMSO-*d*_6_) δ 7.61–7.49 (m, 4H), 7.36 (t, *J* = 7.5 Hz, 2H), 7.31–7.14 (m, 2H), 7.08 (d, *J* = 16.4 Hz, 1H), 6.91 (d, *J* = 8.4 Hz, 2H), 4.96–4.80 (m, 1H), 3.07 (dd, *J* = 12.4, 5.3 Hz, 1H), 3.02–2.74 (m, 3H), 2.12–1.95 (m, 1H), 1.85–1.68 (m, 1H).

##### (*R*)-(*E*)-4-(3-*N*-Ethyl-*N*-methyl-pyrrolidiniumoxy)stilbene Iodide ((*R*)-**20**)

Obtained from (*R*)-(*E*)-4-(3-(*N*-methylpyrrolidinyloxy)stilbene (*R*)-**46** (60 mg, 0.22 mmol, 1 equiv) and ethyl iodide (3 equiv) in DCE (2 mL), at room temperature overnight, according to METHOD B. The desired compound (*R*)-**20** was obtained as a white solid in 99% yield. Mp = 235–236 °C; [α]_D_^25^ = −12.98 (*c* 0.5, MeOH); *R*_t_ (LC-MS) = 3.655 min; LC-MS (ESI): *m*/*z* calcd for C_21_H_26_NO [M]^+^ = 308.20, found 308.2; *R*_t_ (HPLC) = 13.20 min. ^1^H NMR (300 MHz, DMSO-*d*_6_) δ 7.63–7.53 (m, 4H), 7.37 (t, *J* = 7.5 Hz, 2H), 7.28–7.18 (m, 2H), 7.13 (d, *J* = 16.5 Hz, 1H), 7.04–6.96 (m, 2H), 5.34–5.23 (m, 1H), 3.97 (dd, *J* = 13.2, 6.1 Hz, 1H), 3.88–3.70 (m, 2H), 3.68–3.43 (m, 3H), 3.16 (s, 1H), 3.10 (s, 2H), 2.87–2.69 (m, 1H), 2.35–2.15 (m, 1H), 1.31 (t, *J* = 7.2 Hz, 3H). ^13^C NMR (75 MHz, DMSO-*d*_6_) δ 155.8, 137.2, 130.73, 130.71, 128.7, 128.0, 127.7, 127.3, 126.8, 126.2, 115.74, 115.70, 74.7, 74.6, 67.5, 62.4, 62.0, 60.0, 59.5, 48.7, 29.8, 29.3, 8.8.

##### (*R*)-(*E*)-4-(3-*N*,*N*-Diethyl-pyrrolidiniumoxy)stilbene Iodide ((*R*)-**21**)

In an oven-dried three-neck round-bottom flask, under inert atmosphere at −15 °C, NaH (3.6 mg, 0.36 mmol, 1.1 equiv) was suspended in dry THF (1 mL). After dropwise addition of a solution of (*R*)-(*E*)-3-(4-stilbenoxy)pyrrolidine (*R*)-**51** (80 mg, 0.30 mmol, 1 equiv) in THF (1 mL), the reaction mixture was warmed to 40 °C and stirred for 1 h. Then, the mixture was cooled to room temperature and ethyl iodide (103 mg, 0.66 mmol, 2.2 equiv) was added dropwise. Upon stirring at 40 °C overnight, the mixture was cooled to room temperature and it was diluted with Et_2_O. The suspension was filtered, and the filtrate was dried under reduced pressure. The residue was redissolved in THF (1 mL) and ethyl iodide (103 mg, 0.66 mmol, 2.2 equiv) was added dropwise. The reaction mixture was stirred at room temperature overnight, and then diluted with Et_2_O. The resulting suspension was filtered, and the solid was washed with Et_2_O and dried under vacuum. The desired compound (*R*)-**21** was obtained as a white solid in 53% yield. Mp = 217–218 °C; [α]_D_^25^ = −15.94 (*c* 0.5, MeOH); *R*_t_ (LC-MS) = 3.715 min; LC-MS (ESI): *m*/*z* calcd for C_22_H_28_NO [M]^+^ = 322.22, found 322.2; *R*_t_ (HPLC) = 13.47 min. ^1^H NMR (300 MHz, CD_3_OD) δ 7.60–7.48 (m, 4H), 7.33 (t, *J* = 7.5 Hz, 2H), 7.22 (t, *J* = 7.5 Hz, 1H), 7.14 (d, *J* = 16.4 Hz, 1H), 7.06 (d, *J* = 16.4 Hz, 1H), 6.98 (d, *J* = 8.8 Hz, 2H), 5.40–5.24 (m, 1H), 3.99–3.81 (m, 3H), 3.78–3.56 (m, 3H), 3.50 (q, *J* = 7.3 Hz, 2H), 2.85–2.65 (m, 1H), 2.51–2.33 (m, 1H), 1.45–1.32 (m, 6H). ^13^C NMR (75 MHz, CD_3_OD) δ 157.2, 138.9, 133.1, 129.7, 129.1, 128.8, 128.5, 128.4, 127.4, 116.9, 76.2, 67.9, 62.3, 57.5, 57.2, 31.1, 9.1, 9.0.

##### (*E*)-4-(Vinyloxy)stilbene (**52**)

In an oven-dried round-bottom-flask and under inert atmosphere, (*E*)-4-(2-bromoethoxy)-stilbene **28** (1.60 g, 5.28 mmol, 1 equiv) and potassium *tert*-butoxide (2.37 g, 21.12 mmol, 4 equiv) were suspended in 20 mL of anhydrous THF and refluxed for 4 h. The reaction mixture was then cooled to room temperature, filtrated, and concentrated under vacuum to remove most of the THF. The crude mixture was diluted with Et_2_O and washed with water. The organic layer was dried over anhydrous Na_2_SO_4_, filtrated, and concentrated under reduced pressure. The crude was dissolved in cyclohexane and washed with MeOH, providing the desired compound **52** in 89% yield as a white powder. Mp = 117–120 °C; *R*_*f*_ (cyclohexane) = 0.33. ^1^H NMR (300 MHz, CDCl_3_) δ 7.47 (t, *J* = 7.6 Hz, 4H), 7.35 (t, *J* = 7.6 Hz, 2H), 7.02 (m, 3H), 6.91 (d, *J* = 8.6 Hz, 2H), 6.66 (dd, *J* = 13.7, 6.1 Hz, 1H), 4.79 (dd, *J* = 13.7, 1.6 Hz, 1H), 4.46 (dd, *J* = 6.1, 1.6 Hz, 1H).

##### Ethyl (±)-(l)-2-(4-(*E*)-Stilbenoxy)cyclopropane-1-carboxylate ((±)-**53**) and Ethyl (±)-(*u*)-2-(4-(*E*)-Stilbenoxy)cyclopropane-1-carboxylate ((±)-**54**)

In an oven-dried three-neck round-bottom flask under inert atmosphere, at 0 °C, (*E*)-4-(vinyloxy)stilbene **52** (1.00 g, 4.5 mmol, 1 equiv) and Rh_2_(OAc)_4_ (2.0 mg, 0.005 mmol, 0.001 equiv) were dissolved in dry DCM (20 mL). Under vigouros stirring, a cold solution of ethyl diazoacetate (0.473 mL, 513 mg, 4.5 mmol, 1 equiv) in 4 mL of anhydrous DCM was added dropwise at a controlled rate (8 drops every 5 min) at 0 °C. After stirring at 0 °C for 2 h, the reaction mixture was filtered through silica using DCM. The solvent was evaporated under reduced pressure and the resulting residue was purified through flash silica chromatography (gradient from cyclohexane/EtOAc 98:2 to 80:20). The desired compound (±)-**53** was obtained as an off-white solid in 36% yield. *R*_*f*_ (cyclohexane/EtOAc 95:5) = 0.27; mp = 93–94 °C. ^1^H NMR (300 MHz, CDCl_3_) δ 7.53–7.43 (m, 4H), 7.36 (t, *J* = 7.4 Hz, 2H), 7.31–7.19 (m, 1H), 7.08 (d, *J* = 16.4 Hz, 1H), 7.04–6.94 (m, 3H), 4.22 (qd, *J* = 7.1, 1.4 Hz, 2H), 4.10 (ddd, *J* = 6.5, 4.1, 2.1 Hz, 1H), 1.97 (ddd, *J* = 9.8, 6.1, 2.1 Hz, 1H), 1.52 (q, *J* = 6.5, 6.1 Hz, 1H), 1.44 (ddd, *J* = 9.8, 6.1, 4.1 Hz, 1H), 1.31 (t, *J* = 7.1 Hz, 3H). The desired compound (±)-**54** was obtained as an off-white solid in 33% yield. *R*_*f*_ (cyclohexane/EtOAc 95:5) = 0.15; mp = 136–137 °C. ^1^H NMR (300 MHz, CDCl_3_) δ 7.52–7.41 (m, 4H), 7.34 (t, *J* = 7.6 Hz, 2H), 7.25–7.20 (m, 1H), 7.10–7.00 (m, 3H), 6.97 (d, *J* = 16.4 Hz, 1H), 4.08–3.94 (m, 3H), 2.03 (dt, *J* = 8.8, 6.6 Hz, 1H), 1.78 (td, *J* = 6.6, 4.6 Hz, 1H), 1.33 (dt, *J* = 8.8, 6.6 Hz, 1H), 1.06 (t, *J* = 7.1 Hz, 3H).

##### (±)-(l)-2-(4-(*E*)-Stilbenoxy)cyclopropane-1-carboxylic Acid ((±)-**55**)

Obtained by hydrolysis of ethyl (±)-(*l*)-2-(4-(*E*)-stilbenoxy)cyclopropane-1-carboxylate (±)-**53** (470 mg, 1.53 mmol, 1 equiv) according to METHOD E, as a white solid in 95% yield. Mp = 172–173 °C, *R*_*f*_ (cyclohexane/EtOAc 9:1 + 1% HCOOH) = 0.38. ^1^H NMR (300 MHz, CDCl_3_) δ 7.52–7.44 (m, 4H), 7.35 (t, *J* = 7.5 Hz, 2H), 7.25–7.21 (m, 1H), 7.08 (d, *J* = 16.3 Hz, 1H), 7.03–6.95 (m, 3H), 4.15 (ddd, *J* = 6.5, 4.3, 2.1 Hz, 1H), 1.99 (ddd, *J* = 9.7, 6.1, 2.1 Hz, 1H), 1.64–1.50 (m, 2H).

##### (±)-(*u*)-2-(4-(*E*)-Stilbenoxy)cyclopropane-1-carboxylic Acid ((±)-**56**)

Obtained by hydrolysis of ethyl (±)-(*u*)-2-(4-(*E*)-stilbenoxy)cyclopropane-1-carboxylate (±)-**54** (453 mg, 1.47 mmol, 1 equiv) according to METHOD E, as a white solid in 78% yield. Mp = 183 °C (dec); *R*_*f*_ (cyclohexane/EtOAc 9:1 + 1% HCOOH) = 0.28. ^1^H NMR (300 MHz, DMSO-*d*_6_) δ 12.09 (bs, 1H), 7.63–7.50 (m, 4H), 7.36 (t, *J* = 7.5 Hz, 2H), 7.28–7.16 (m, 2H), 7.10 (d, *J* = 16.4 Hz, 1H), 7.04 (d, *J* = 8.8 Hz, 2H), 4.14 (td, *J* = 6.7, 4.6 Hz, 1H), 1.98 (dt, *J* = 8.6, 6.7 Hz, 1H), 1.44 (ddd, *J* = 6.7, 5.8, 4.6 Hz, 1H), 1.31 (ddd, *J* = 8.5, 6.7, 5.7 Hz, 1H).

##### *tert*-Butyl ((±)-(l)-2-(4-(*E*)-Stilbenoxy)cyclopropyl)carbamate ((±)-**57**)

Obtained from (±)-(*l*)-2-(4-(*E*)-stilbenoxy)cyclopropane-1-carboxylic acid (±)-**55** (200 mg, 0.71 mmol, 1 equiv) according to METHOD F. The desired compound (±)-**57** was obtained as a white solid in 91% yield after purification by silica gel flash column chromatography (gradient from cyclohexane to cyclohexane/EtOAc 8:2). Mp = 130–131 °C; *R*_*f*_ (cyclohexane/EtOAc 8:2) = 0.53. ^1^H NMR (300 MHz, CDCl_3_) δ 7.52–7.43 (m, 4H), 7.35 (t, *J* = 7.4 Hz, 2H), 7.25–7.20 (m, 1H), 7.12–7.03 (m, 3H), 6.98 (d, *J* = 16.4 Hz, 1H), 4.74–4.63 (m, 1H), 3.74 (ddd, *J* = 7.0, 3.8, 1.3 Hz, 1H), 2.88 (ddt, *J* = 8.6, 4.9, 2.1, 1.3 Hz, 1H), 1.47 (s, 9H), 1.21 (ddd, *J* = 8.7, 7.0, 3.8 Hz, 1H), 1.08 (td, *J* = 7.0, 4.9 Hz, 1H).

##### *tert*-Butyl ((±)-(*u*)-2-(4-(*E*)-Stilbenoxy)cyclopropyl)carbamate ((±)-**58**)

Obtained from (±)-(*u*)-2-(4-(*E*)-stilbenoxy)cyclopropane-1-carboxylic acid (±)-**56** (200 mg, 0.71 mmol, 1 equiv) according to METHOD F. The desired compound (±)-**58** was obtained as a white solid in 65% yield after purification by silica gel flash column chromatography (gradient from cyclohexane to cyclohexane/EtOAc 8:2). Mp = 134–136 °C; *R*_*f*_ (cyclohexane/DCM 1:1) = 0.33. ^1^H NMR (300 MHz, CDCl_3_) δ 7.53–7.44 (m, 4H), 7.35 (t, *J* = 7.5 Hz, 2H), 7.28–7.21 (m, 1H), 7.06 (d, *J* = 8.1 Hz, 3H), 6.99 (d, *J* = 16.3 Hz, 1H), 4.85–4.74 (m, 1H), 3.77 (td, *J* = 6.3, 3.9 Hz, 1H), 3.05–2.94 (m, 1H), 1.41 (s, 9H), 1.33–1.21 (m, 1H), 0.85–0.73 (m, 1H).

##### (±)-(l)-2-(4-(*E*)-Stilbenoxy)cyclopropan-1-amine Hydrochloride ((±)-**59**)

Obtained from *tert*-butyl ((±)-(*l*)-2-(4-(*E*)-stilbenoxy)cyclopropyl)carbamate (±)-**57** (228 mg, 0.65 mmol, 1 equiv) according to METHOD G as a white solid in 58% yield. Mp = 128–129 °C; *R*_*f*_ (cyclohexane/EtOAc 8:2 + 1% DIPEA) = 0.24. ^1^H NMR (300 MHz, CD_3_OD) δ 7.61–7.48 (m, 4H), 7.34 (t, *J* = 7.8 Hz, 2H), 7.26–7.19 (m, 1H), 7.14–7.02 (m, 4H), 4.14 (ddd, *J* = 6.8, 5.2, 1.6 Hz, 1H), 2.95 (ddd, *J* = 7.7, 6.4, 1.6 Hz, 1H), 1.46–1.33 (m, 2H).

##### (±)-(*u*)-2-(4-(*E*)-Stilbenoxy)cyclopropan-1-amine Hydrochloride ((±)-**60**)

Obtained from *tert*-butyl ((±)-(*u*)-2-(4-(*E*)-stilbenoxy)cyclopropyl)carbamate (±)-**58** (405 mg, 1.15 mmol, 1 equiv) according to METHOD G as a white solid in 75% yield. *R*_*f*_ (cyclohexane/EtOAc 8:2 + 1% DIPEA) = 0.36. Mp = 132–133 °C.

^1^H NMR (300 MHz, CD_3_OD) δ 7.61–7.50 (m, 4H), 7.34 (t, *J* = 7.5 Hz, 2H), 7.28–7.17 (m, 1H), 7.17–7.08 (m, 4H), 4.08 (td, *J* = 6.2, 3.7 Hz, 1H), 3.00–2.86 (m, 1H), 1.50–1.38 (m, 1H), 1.08–0.97 (m, 1H).

##### (±)-(l)-*N*,*N*-Diethyl-2-(4-(*E*)-stilbenoxy)cyclopropan-1-amine ((±)-**61**)

Obtained from (±)-(*l*)-2-(4-((*E*)-stilbenoxy)cyclopropan-1-amine hydrochloride (±)-5**9** (96 mg, 0.38 mmol) according to METHOD H, in 53% yield. Mp = 65–70 °C. *R*_*f*_ (cyclohexane/AcOEt 8:2 + 1% DIPEA) = 0.54. ^1^H NMR (300 MHz, CD_3_OD) δ 7.54–7.46 (m, 4H), 7.32 (t, *J* = 7.5 Hz, 2H), 7.26–7.15 (m, 1H), 7.12 (d, *J* = 16.4 Hz, 1H), 7.06–6.99 (m, 3H), 3.75 (ddd, *J* = 6.6, 3.5, 1.5 Hz, 1H), 2.87–2.64 (m, 4H), 2.18 (ddd, *J* = 8.4, 5.1, 1.5 Hz, 1H), 1.16–1.09 (m, 7H), 1.01 (td, *J* = 6.6, 5.1 Hz, 1H).

##### (±)-(*u*)-*N*,*N*-Diethyl-2-(4-(*E*)-stilbenoxy)cyclopropan-1-amine ((±)-**62**)

Obtained from (±)-(*l*)-2-(4-((*E*)-stilbenoxy)cyclopropan-1-amine hydrochloride (±)-**60** (148 mg, 0.51 mmol, 1 equiv) according to METHOD H, in 32% yield. Mp = 62–63 °C; *R*_*f*_ (DCM/MeOH 8:2 + 1% DIPEA) = 0.58. ^1^H NMR (300 MHz, CDCl_3_) δ 7.51–7.42 (m, 4H), 7.34 (t, *J* = 7.6 Hz, 2H), 7.25–7.20 (m, 1H), 7.12–7.03 (m, 3H), 6.97 (d, *J* = 16.3 Hz, 1H), 3.73–3.56 (m, 1H), 2.95–2.66 (m, 4H), 2.08–1.90 (m, 1H), 1.17–1.06 (m, 6H), 0.93–0.76 (m, 2H).

##### (±)-(l)-*N*,*N*-Diethyl-*N*-methyl-2-(4-(*E*)-stilbenoxy)cyclopropan-1-ammonium Iodide ((±)-**26**)

Obtained from (±)-(*l*)-*N*,*N*-diethyl-2-(4-((*E*)-stilbenoxy)cyclopropan-1-amine (±)-**61** (63 mg, 0.21 mmol, 1 equiv) according to METHOD B, using methyl iodide as a solvent (3 mL) at reflux temperature for 4 h. The desired product (±)-**26** was obtained as a white solid in 50% yield after trituration from AcOEt. Mp = 194 °C (dec). *R*_t_ (LC-MS) = 3.621 min; LC/MS (ESI): *m*/*z* calcd for C_22_H_28_NO [M]^+^ = 322.2, found 322.2; *R*_t_ (HPLC) = 13.43 min; ^1^H NMR (300 MHz, CD_3_OD) δ 7.57–7.50 (m, 4H), 7.33 (t, *J* = 7.5 Hz, 2H), 7.26–7.19 (m, 1H), 7.14 (d, *J* = 16.4 Hz, 1H), 7.11–7.02 (m, 3H), 4.67 (ddd, *J* = 8.0, 4.4, 2.3 Hz, 1H), 3.64–3.41 (m, 5H), 2.88 (s, 3H), 1.93 (ddd, *J* = 8.7, 8.0, 6.3 Hz, 1H), 1.53–1.39 (m, 7H). ^13^C NMR (75 MHz, CD_3_OD) δ 158.1, 138.9, 133.2, 129.7, 128.90, 128.86, 128.5, 128.4, 127.4, 116.8, 61.1, 60.8, 52.6, 50.9, 45.1, 13.0, 8.6, 8.5.

##### (±)-(*u*)-*N*,*N*-Diethyl-*N*-methyl-2-(4-(*E*)-stilbenoxy)cyclopropan-1-ammonium Iodide ((±)-**27**)

Obtained from (±)-(*u*)-*N*,*N*-diethyl-2-(4-((*E*)-stilbenoxy)cyclopropan-1-amine (±)-**62** (11 mg 0.035 mmol, 1 equiv), according to METHOD B, using methyl iodide as a solvent (2 mL) at reflux temperature overnight. The desired product (±)-**27** was obtained as a white solid in 41% yield after trituration with EtOAc. *R*_t_ (LC-MS) = 3.747 min; LC/MS (ESI): *m*/*z* calcd for C_22_H_28_NO [M]^+^ = 322.2, found 322.2; *R*_t_ (HPLC) = 13.57 min; ^1^H NMR (300 MHz, CD_3_OD) δ 7.62–7.50 (m, 4H), 7.34 (t, *J* = 7.5 Hz, 2H), 7.27–7.20 (m, 1H), 7.20–7.12 (m, 3H), 7.08 (d, *J* = 16.4 Hz, 1H), 4.18 (td, *J* = 6.5, 4.4 Hz, 1H), 3.85–3.50 (m, 4H), 3.20 (dt, *J* = 9.3, 6.5 Hz, 1H), 3.10 (s, 3H), 1.76–1.59 (m, 2H), 1.55 (t, *J* = 7.3 Hz, 3H), 1.47 (t, *J* = 7.2 Hz, 3H). ^13^C NMR (75 MHz, CD_3_OD) δ 157.9, 138.9, 133.7, 129.7, 129.0, 128.8, 128.7, 128.5, 127.4, 116.4, 62.5, 53.9, 46.7, 11.4, 8.79, 8.76.

### Biological Assays

#### Affinity to α7, α3β4, and α4β2 Nicotinic Receptors

For (±)-[^3^H]epibatidine (specific activity of 56–60 Ci/mmol; PerkinElmer, Boston MA), saturation binding studies were carried out on membrane homogenates. These were prepared from either SH-EP1 cells stably transfected with α3- and β4-nAChR subunit cDNAs,^16^ or HEK 293 cells stably transfected with the α4 and β2 cDNAs (generous gift of Dr. Jon Lindstrom).^17^

For saturation experiments, the membrane homogenate aliquots were incubated overnight at 4 °C with 0.01–5 nM concentrations of (±)-[^3^H]epibatidine. Nonspecific binding was determined in parallel by adding to the incubation solutions 100 nM unlabeled epibatidine (Sigma-Aldrich) as described previously.^31^ At the end of the incubation, the samples were filtered on a GFC filter soaked in 0.5% polyethylenimine and washed with 10 mL of ice-cold phosphate-buffered saline (PBS), and the filters were counted in a β counter.

For [^125^I]-αBungarotoxin ([^125^I]αBgtx) (specific activity 200–213 Ci/mmol, PerkinElmer, Boston MA), saturation binding studies were carried out on membrane homogenate prepared from SH-SY5Y cells transfected with human α7 cDNA, as described previously.^12^ Aliquots of the membrane homogenates were incubated overnight with 0.1–10.0 nM concentrations of [^125^I]Bgtx at r.t. Nonspecific binding was determined in parallel by including in the assay mixture 1 μM unlabeled αBgtx (Sigma-Aldrich). After incubation, the samples were filtered as described for (±)-[^3^H]epibatidine binding.

For competition studies, the inhibition of [^3^H]epibatidine and [^125^I] αBgtx binding was measured by incubating the membranes transfected with the appropriate subtype with increasing concentrations of the compounds (1 nM–1 mM) 5 min followed by overnight incubation at 4 °C, with [^3^H]epibatidine 0.1 nM for the α4β2 subtype or [^3^H]epibatidine 0.25 nM for the α3β4 subtype or at r.t. with [^125^I]αBgtx 2–3 nM in the case of the α7 subtype. At the end of the incubation time, the samples were processed as described for the saturation studies.

[^3^H]epibatidine binding was determined by liquid scintillation counting in a β counter, and [^125^I] αBgtx binding by direct counting in a γ counter. Saturation binding data were evaluated by one-site competitive binding curve-fitting procedures using GraphPad Prism version 6 (GraphPad Software, CA). In the saturation binding assay, the maximum specific binding (*B*_max_) and the equilibrium binding constant (*K*_d_) values were calculated using one site-specific binding with Hill slope–model. *K*_i_ values were obtained by fitting three independent competition binding experiments, each performed in duplicate for each compound on each subtype. Inhibition constants (*K*_i_) were estimated by reference to the *K*_d_ of the radioligand, according to the Cheng–Prusoff equation and are expressed as nM values.

#### Two-Electrode Voltage Clamp (TEVC) Recording of α7- and α9α10-nAChR Function

For functional pharmacology studies, two-electrode voltage clamp recordings were performed, using human nAChR subunits heterologously expressed in *X. laevis* oocytes. Approaches were closely related to those previously detailed.^19^ Briefly, *X. laevis* oocytes were purchased from Ecocyte Bioscience US (Austin, TX), and the incubation temperature was 13 °C. Harvesting of oocytes from *X. laevis* by EcoCyte follows the guidelines of the National Institute of Health’s Office of Laboratory Animal Welfare and was authorized under IACUC number #1019-1 (valid through December 2022). Injections of nAChR subunit mRNA were made using glass micropipettes (outer diameter ≈40 μm, resistance 2–6 MΩ), and mRNA was injected in a total volume of 40 nL. For α7-nAChR, 1.25 ng of α7-nAChR subunit mRNA was injected per oocyte, along with 0.125 ng of NACHO mRNA to improve functional expression.^32^ For α9α10-nAChR, a total of 10 ng of nAChR subunit mRNA was injected using α9 to α10 cRNAs in a 9:1 ratio by mass.

TEVC recordings were made in oocyte saline solution (82.5 mM NaCl, 2.5 mM KCl, 5 mM HEPES, 1.8 mM CaCl_2_·_2_H_2_O, and 1 mM MgCl_2_·_6_H_2_O, pH 7.4), and were performed at room temperature (20 °C). One week after injection, oocytes were voltage-clamped (−70 mV; Axoclamp 900A amplifier, Molecular Devices, Sunnyvale, CA). Recordings were sampled at 10 kHz (low-pass Bessel filter, 40 Hz; high-pass filter, DC), and saved to disk (Clampex v10.2; Molecular Devices). To ensure quality of recordings, oocytes with leak currents (*I*_leak_) > 50 nA were discarded without being recorded from. In all cases, initial control stimulations (ACh, 1 mM, applied for 1 s) were performed, with 60 s washout (no drug) between control stimulations (total of 5 stimulations). This allowed us to define a 100% response control, and to ascertain that run-down or desensitization was not occurring due to repeated ACh stimulation.

For antagonist concentration–response curves, test compounds were applied simultaneously with 1 mM ACh, starting with the lowest concentration of test compound and increasing in half-log steps to a maximum concentration of 100 μM. The standard 1 min spacing between stimulation was maintained. Data for each oocyte were normalized by expressing peak function in the presence of test compounds as % of control function (the mean peak function measured across the initial control stimulations was defined as 100% for each oocyte). IC_50_ values were calculated from these normalized nAChR-mediated currents through nonlinear least-squares curve fitting (GraphPad Prism 5.0; GraphPad Software, Inc., La Jolla, CA).

Intrinsic agonist efficacy of test compounds was measured by applying them (alone at 100 μM, 1 s application time, no ACh co-application) 1 min following the last initial control stimulation. Peak function following addition of the test compound was normalized for each oocyte in the same way just described for antagonist concentration curves. The same normalization was applied to the peak of any rebound current observed during the 60 s washout period following application of the test compound, and to the peak function induced by a final control application of ACh (1 mM, 1 s application time).

## Supporting Information Available

Molecular formula strings (CSV)
^1^H NMR and ^13^C NMR spectra of the final compounds and HPLC traces of **6**, **7**, (±)-**22**, **23**, and (±)-**24** (PDF)
