# Supplementary material for: From 2-Triethylammonium Ethyl Ether of 4-Stilbenol (MG624) to Selective Small-Molecule Antagonists of Human α9α10 Nicotinic Receptor by Modifications at the Ammonium Ethyl Residue
Source: J Med Chem. 2022 Jul 14;65(14):10079–97. doi: 10.1021/acs.jmedchem.2c00746 (PMC9339509; doi:10.1021/acs.jmedchem.2c00746)
Supplement: Supplementary file 2 — jm2c00746_si_002.pdf [file jm2c00746_si_002.pdf]

# From 2-triethylammonium ethyl ether of 4-stilbenol (MG624) to selective small molecule antagonists of human α9α10 nicotinic receptor by modifications at the ammonium ethyl residue

*Francesco Bavo,* [†,#] *Marco Pallavicini,* [†]*Susanna Pucci,* [‡,°] *Rebecca Appiani,* [†] *Alessandro Giraudo,* [†] *Brek Eaton,* [ǂ]*Linda Lucero,* [ǂ] *Cecilia Gotti,*[‡] *Milena Moretti,* [‡,§] *Paul Whiteaker,* [•] *and Cristiano Bolchi**[,†]

[†] Dipartimento di Scienze Farmaceutiche, Università degli Studi di Milano, via Mangiagalli 25, I-20133 Milano, Italy

[#] Department of Drug Design and Pharmacology, University of Copenhagen, DK-2100 Copenhagen, Denmark

[‡] Institute of Neuroscience, CNR, via Vanvitelli 32, I-20129 Milano, Italy

° NeuroMi Milan Center for Neuroscience, University of Milano Bicocca, Italy

[ǂ] Division of Neurobiology, Barrow Neurological Institute, Phoenix, AZ 85013, USA

[§] Department of Medical Biotechnology and Translational Medicine, Università degli Studi di Milano, via Vanvitelli 32, I-20129 Milano, Italy

[•] Department of Pharmacology and Toxicology, Medical College of Virginia Campus, Virginia Commonwealth University, Richmond, VA 23298, USA

Corresponding author's email address: cristiano.bolchi@unimi.it

## Supplementary Information

*$^{1}$H NMR and $^{13}$C-NMR of final compounds* ........ 3

*(E)-4-(2-(N,N-diethyl-N-methylammonium)ethyloxy)stilbene iodide (4).* ........ 3

*(E)-4-(2-(N,N-dimehtyl-N-ethylammonium)ethyloxy)stilbene iodide (5)* ........ 3

*(E)-4-(2-(trimehtylammonium)ethyloxy)stilbene iodide (6).* ........ 4

*(E)-4-(2-(N-cyclohexy-N,N-dimethyl)ammoniumethyloxy)stilbene iodide (7).* ........ 5

*(E)-4-(2-(N,N-dicyclohexyl-N-methyl)ammoniumethyloxy)stilbene iodide (8).* ........ 5

*(E)-4-(2-(N,N-diethyl-N-benzylammonium)ethyloxy)stilbene bromide (9).* ........ 6

*(E)-4-(2-(N,N-dibenzyl, N-methylammonium)ethyloxy)stilbene iodide (10).* ........ 7

*(E)-4-(2-(N,N-dimethyl-N-adamantanyl)amminiumethyloxy)stilbene iodide (11).* ........ 7

*(E)-4-(2-quinuclidiniumehyloxy)stilbene iodide (12).* ........ 8

*(E)-4-(2-(N-methyl)azetidiniumethyloxy)stilbene iodide (13).* ........ 9

*(E)-4-(2-(N-methyl)pyrrolidiniumethyloxy)stilbene iodide (14).* ........ 9

*(E)-4-(2-(N-methyl)piperidiniumethyloxy)stilbene iodide (15).* ........ 10

*(E)-4-(2-(N-Methyl)morpholiniumethyloxy)stilbene iodide (16).* ........ 11

*(E)-4-(2-pyridiniumethyloxy)stilbene iodide (17).* ........ 11

*(E)-4-(3-(N,N-dimethyl-azetidiniumoxy)stilbene iodide (18).* ........ 12

*(±)-(E)-4-(3-(N,N-dimethyl-pyrrolidiniumoxy)stilbene iodide ((±)-19).* ........ 13

*(S)-(E)-4-(3-(N,N-dimethyl-pyrrolidiniumoxy)stilbene iodide ((S)-19).* ........ 13

*(R)-(E)-4-(3-(N,N-dimethyl-pyrrolidiniumoxy)stilbene iodide ((R)-19).* ........ 14

*(R)-(E)-4-(3-(N-ethyl,N-methyl-pyrrolidiniumoxy)stilbene iodide ((R)-20).* ........ 15

*(R)-(E)-4-(3-(N,N-diethyl-pyrrolidiniumoxy)stilbene iodide ((R)-21).* ........ 15

*(±)-(E)-4-(3-(N,N-dimethyl-pyperidiniumoxy)stilbene iodide ((±)-22).* ........ 16

*(E)-4-(4-(N,N-dimethyl-pyperidiniumoxy)stilbene iodide (23).* ........ 17

*(±)-(E)-4-(3-(N-methyl-quinuclidiniumoxy)stilbene iodide ((±)-24).* ........ 17

*(S)-(E)-1-methyl-2-(4-stilbenoxymethyl)pyrrolidinium iodide ((S)-25).* ........ 18

*(R)-(E)-1-methyl-2-(4-stilbenoxymethyl)pyrrolidinium iodide ((R)-25).* ........ 18

*(±)-(l)-N,N-diethyl-N-methyl-2-(4-((E)-stilbenoxy)cyclopropan-1-ammonium iodide ((±)-26).* ........ 19

*(±)-(u)-N,N-diethyl-N-methyl-2-(4-((E)-stilbenoxy)cyclopropan-1-ammonium iodide ((±)-27).* ........ 19

*HPLC traces of lead compounds* ........ 21

## $^{1}$H NMR and $^{13}$C-NMR of final compounds

### *(E)-4-(2-(N,N-diethyl-N-methylammonium)ethyloxy)stilbene iodide (4).*

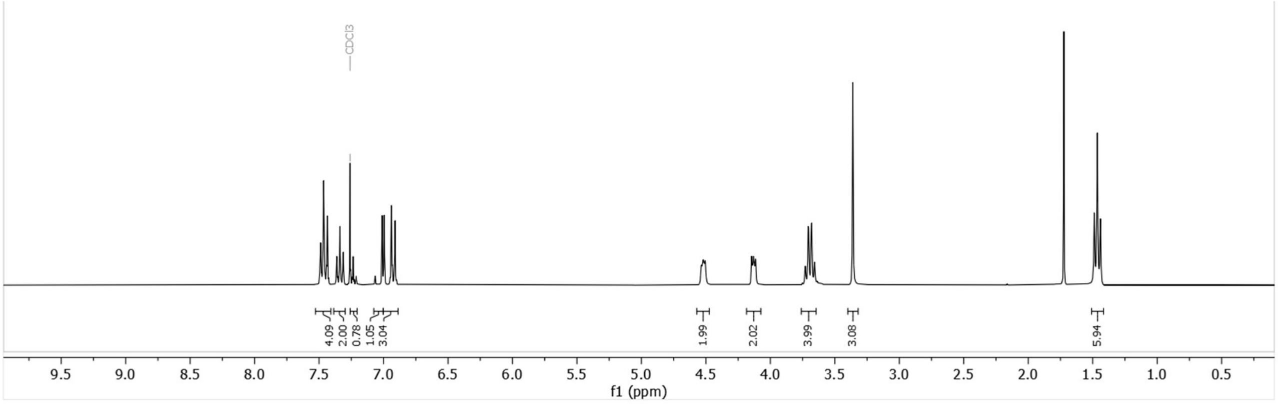


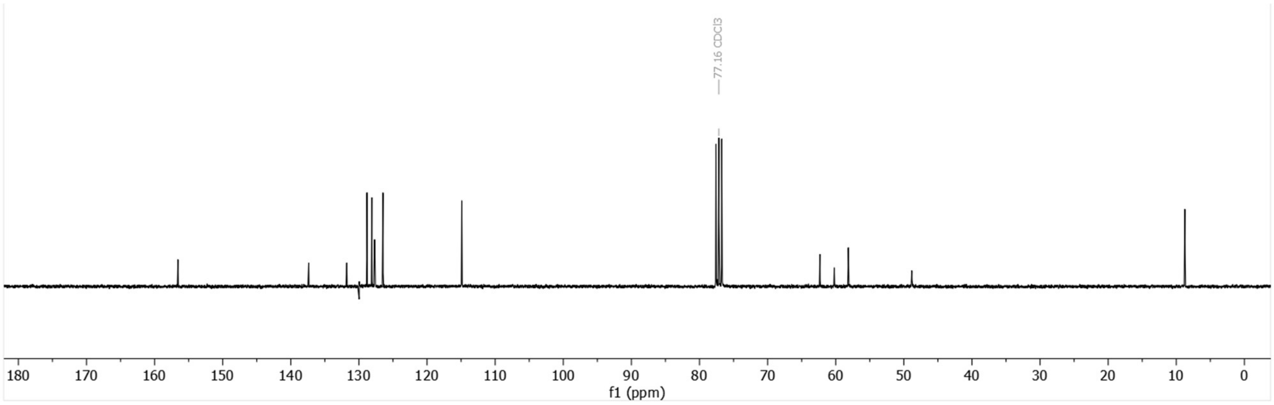


### *(E)-4-(2-(N,N-dimehtyl-N-ethylammonium)ethyloxy)stilbene iodide (5)*

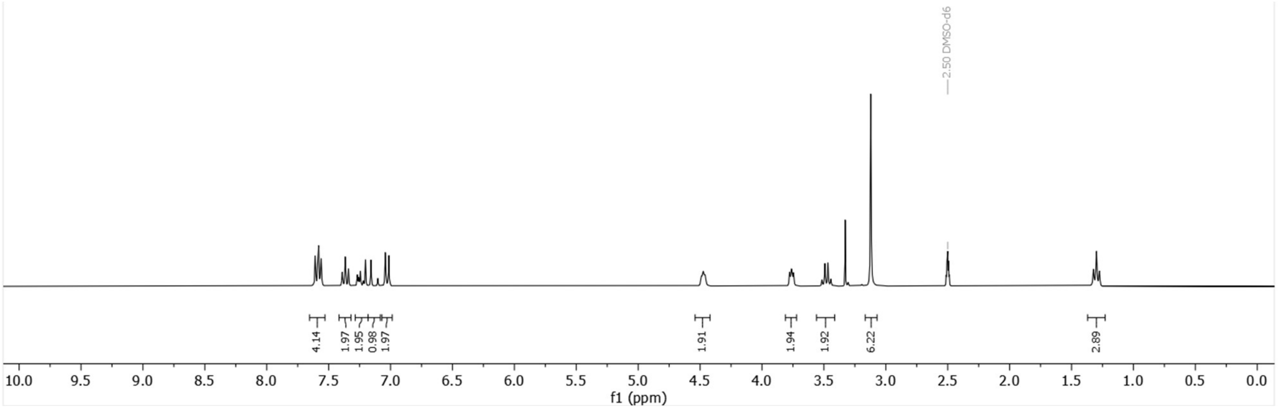

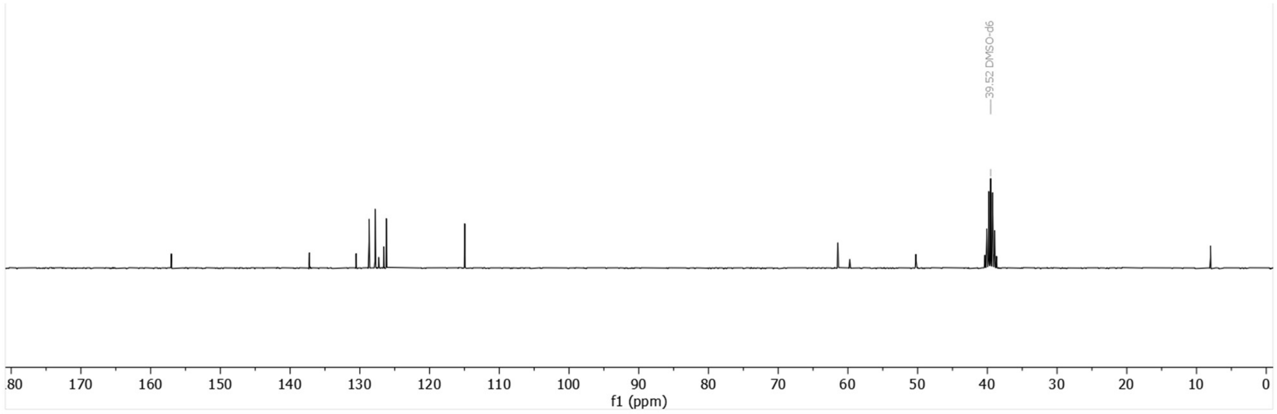


***(E)-4-(2-(trimehtylammonium)ethyloxy)stilbene iodide (6).***

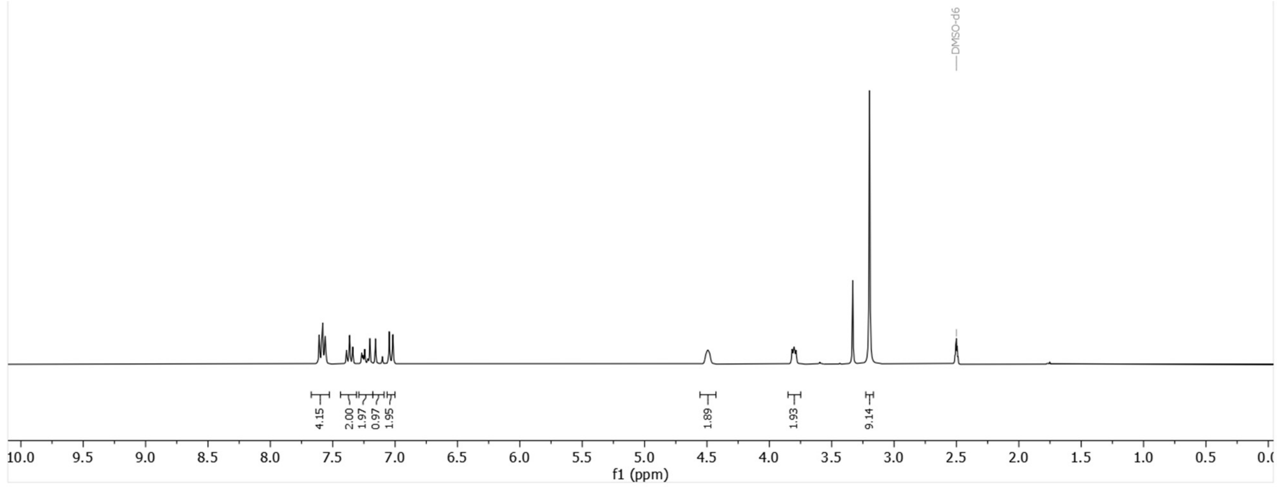


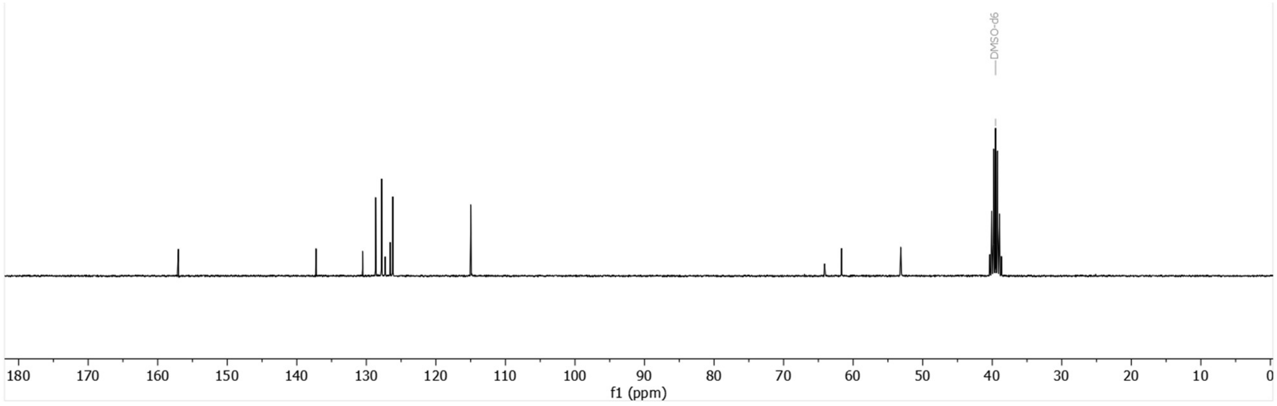

***(E)-4-(2-(N-cyclohexy-N,N-dimethyl)ammoniumethyloxy)stilbene iodide (7).***

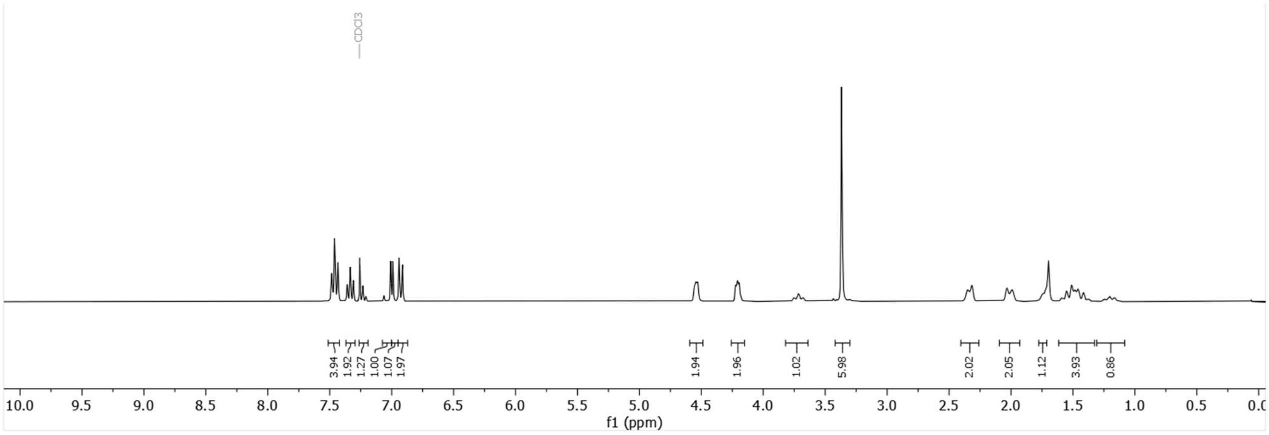


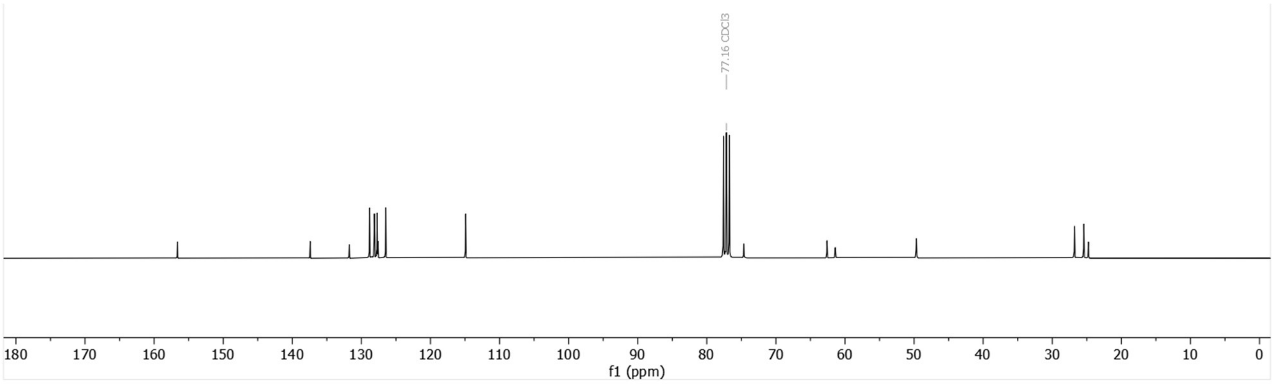


***(E)-4-(2-(N,N-dicyclohexyl-N-methyl)ammoniumethyloxy)stilbene iodide (8).***

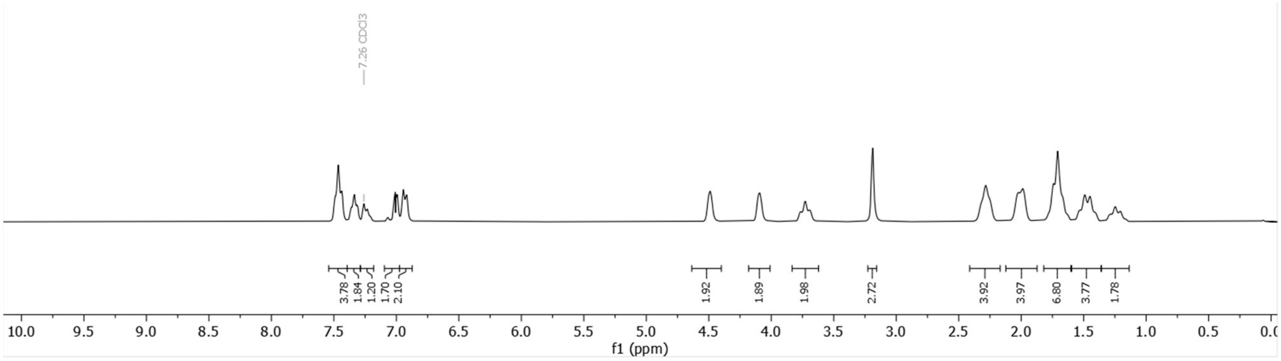

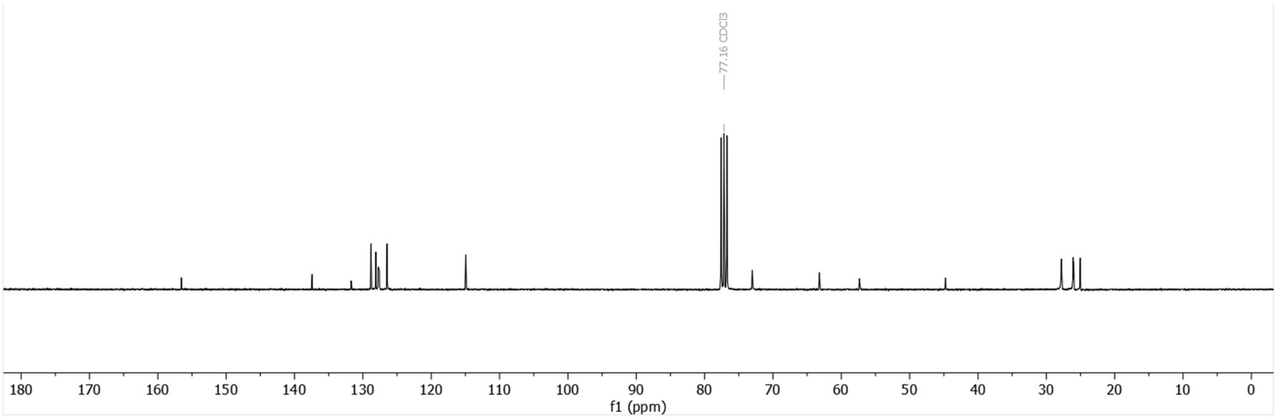


***(E)-4-(2-(N,N-diethyl-N-benzylammonium)ethyloxy)stilbene bromide (9).***

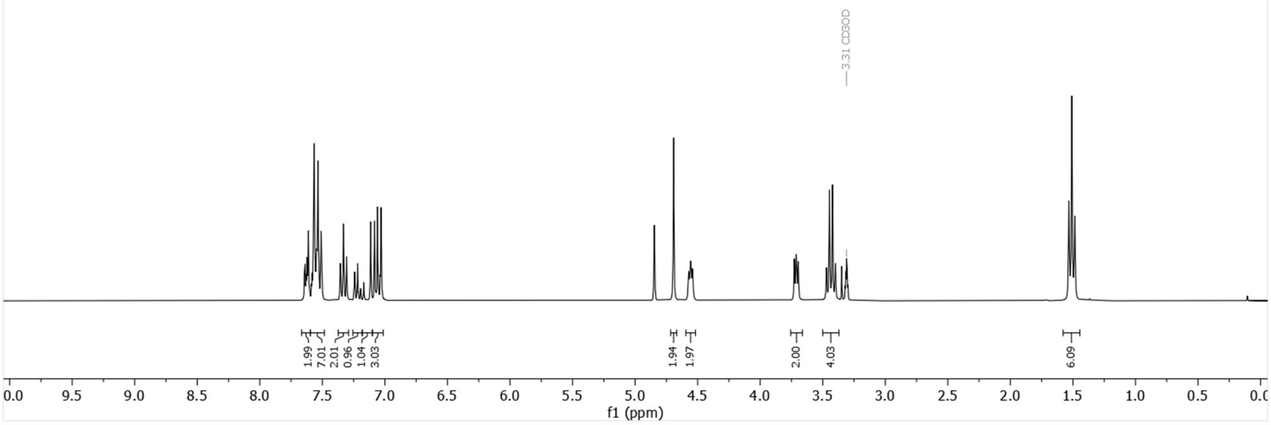


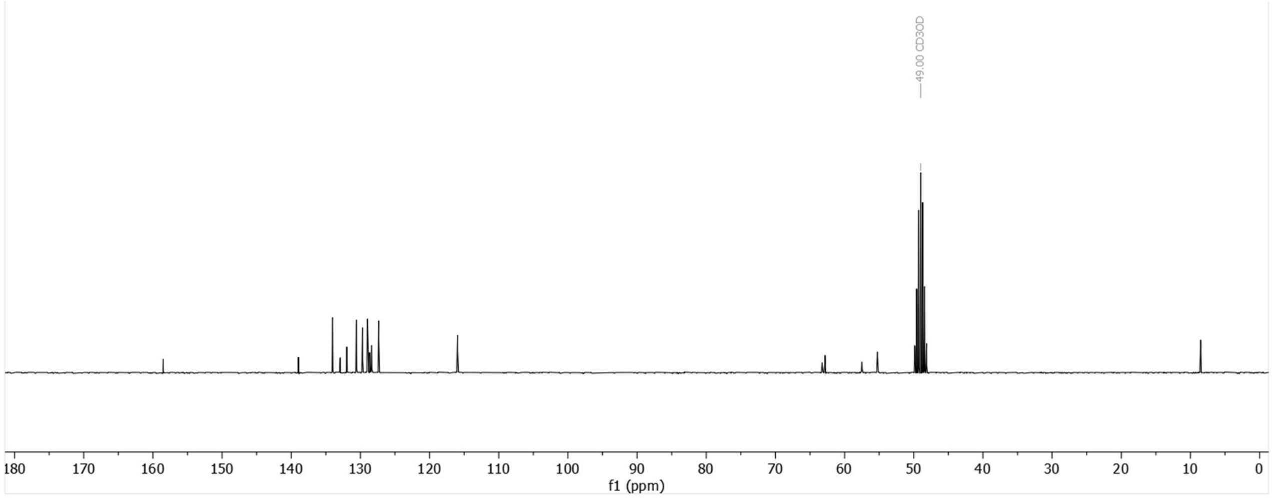

***(E)-4-(2-(N,N-dibenzyl, N-methylammonium)ethyloxy)stilbene iodide (10).***

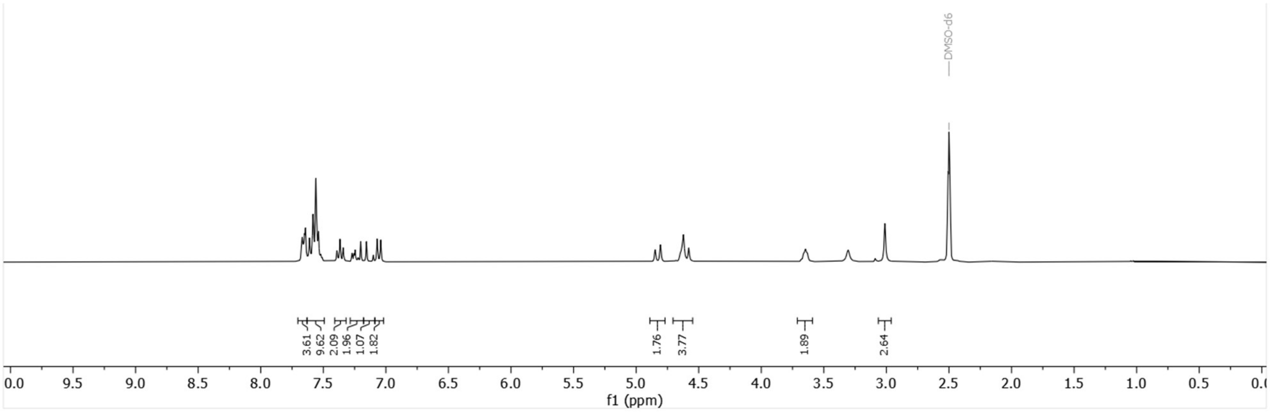


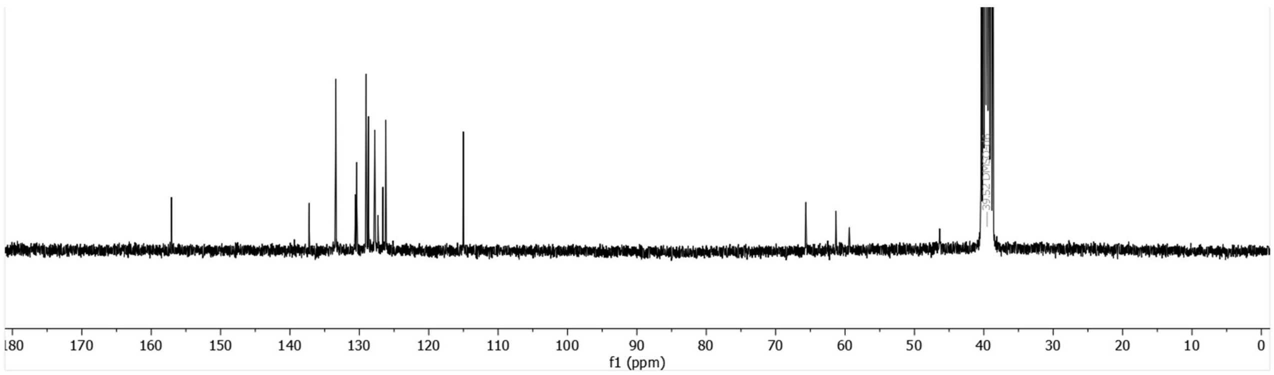


***(E)-4-(2-(N,N-dimethyl-N-adamantanyl)amminiumethyloxy)stilbene iodide (11).***

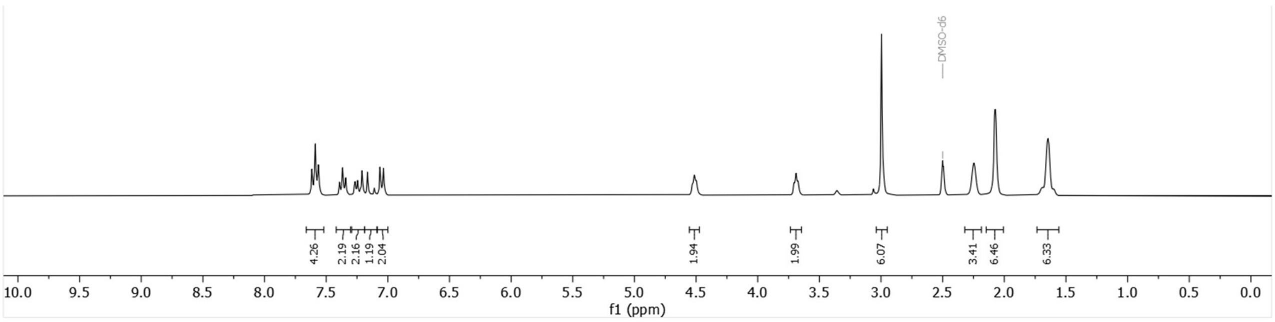

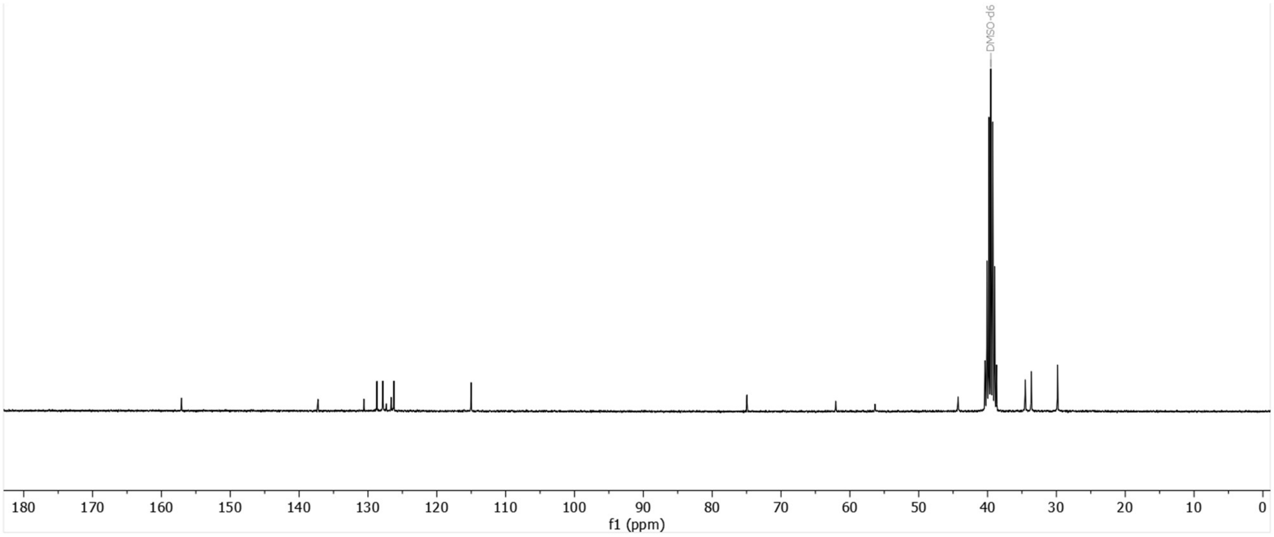


***(E)-4-(2-quinuclidiniumehyloxy)stilbene iodide (12).***

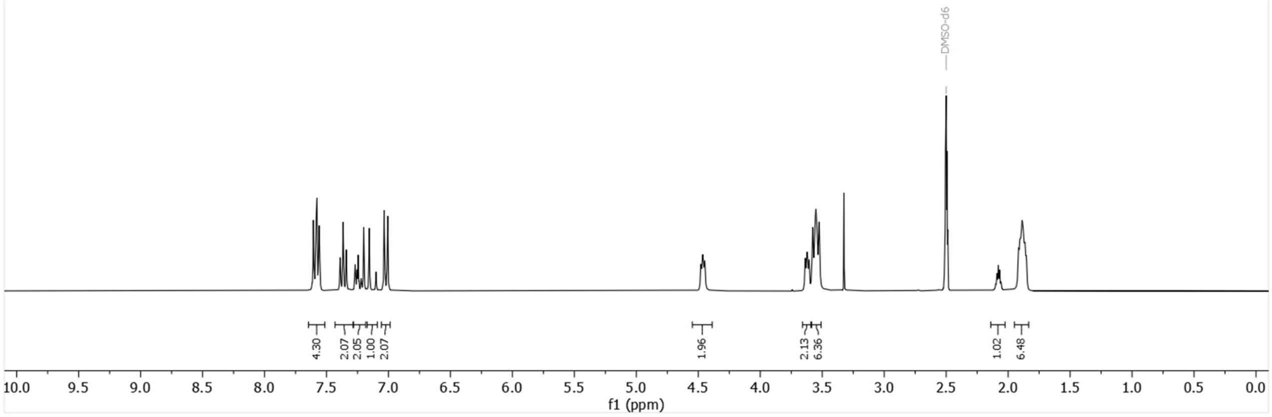


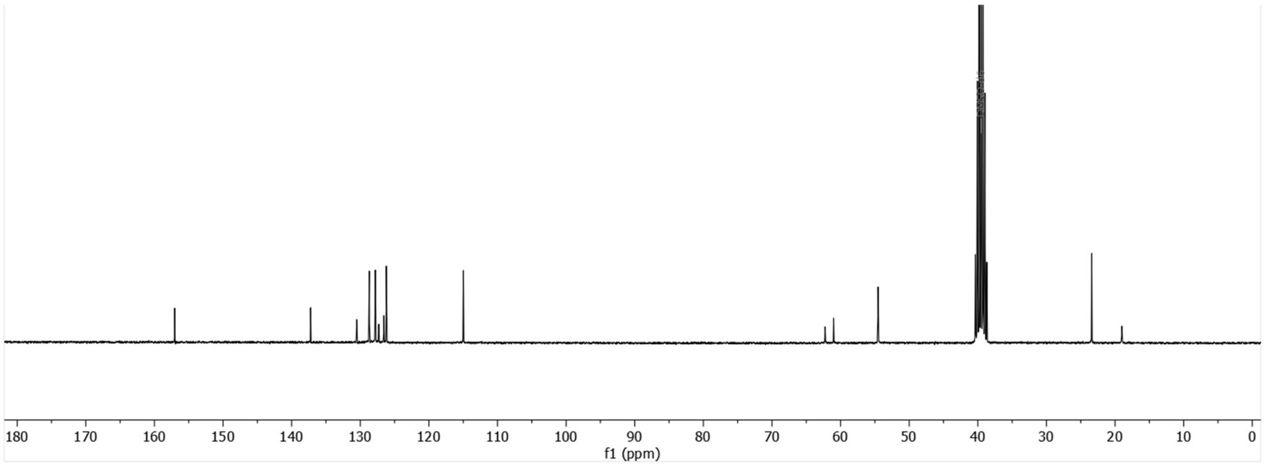

*(E)-4-(2-(N-methyl)azetidiniumethyloxy)stilbene iodide (13).*

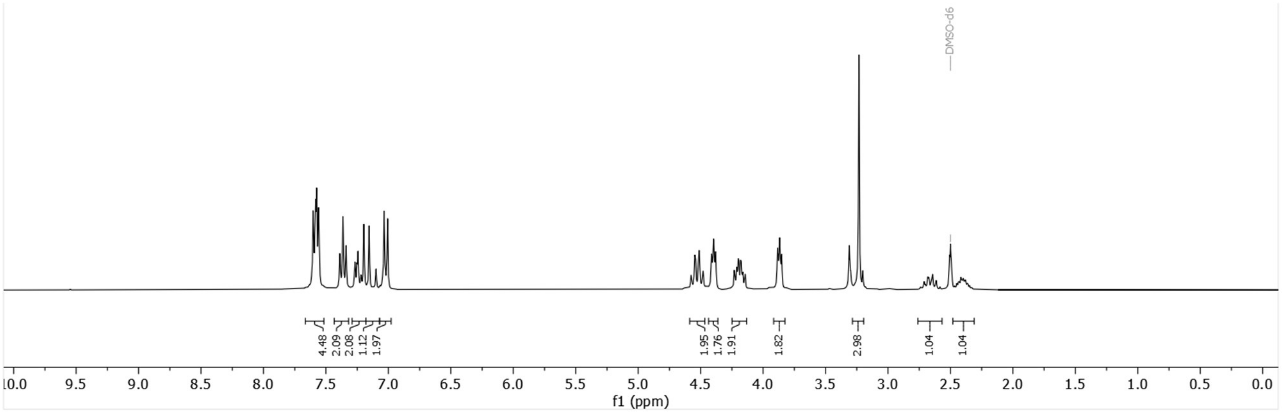


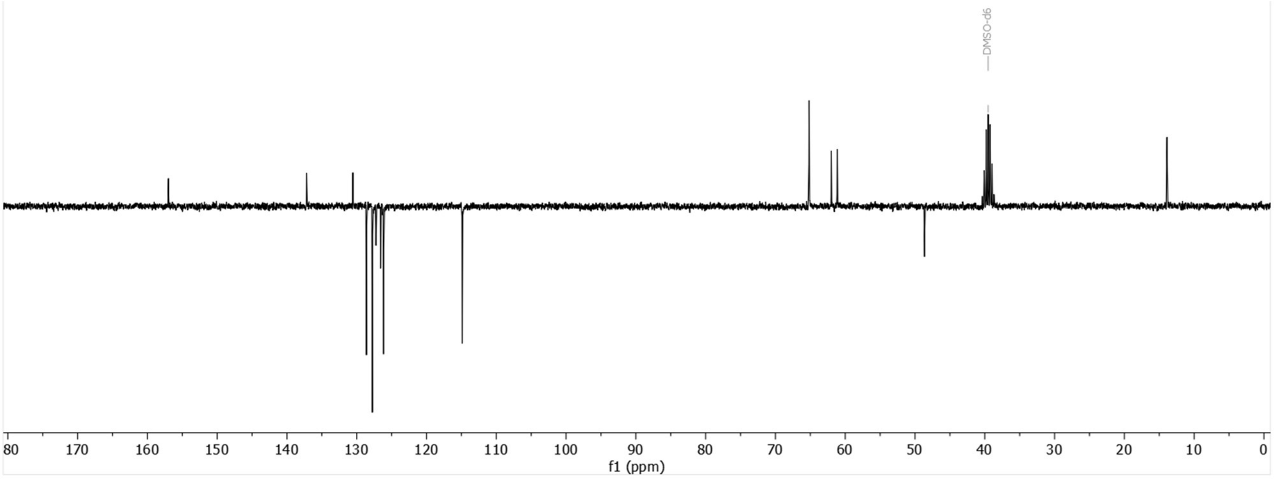


*(E)-4-(2-(N-methyl)pyrrolidiniumethyloxy)stilbene iodide (14).*

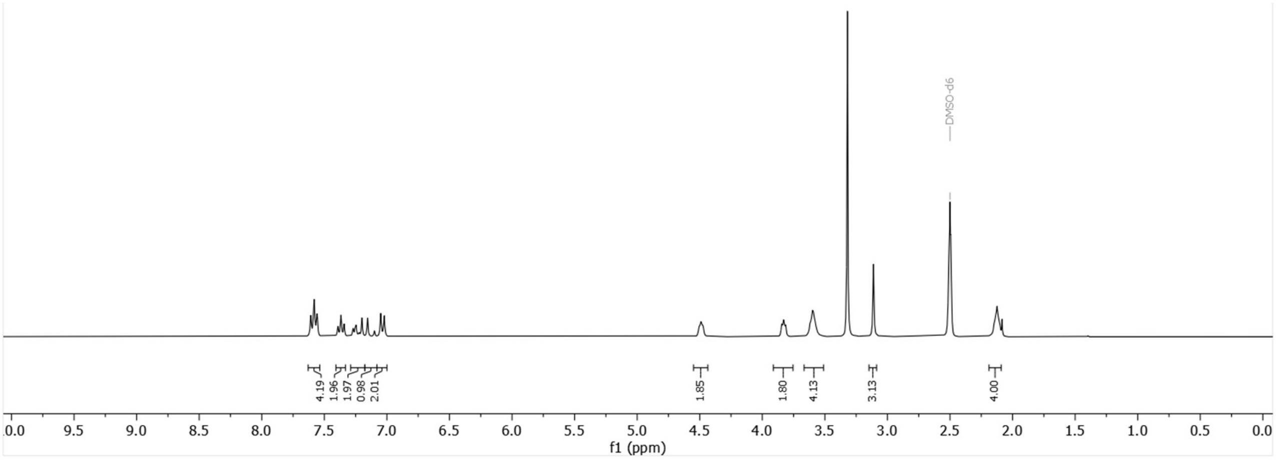

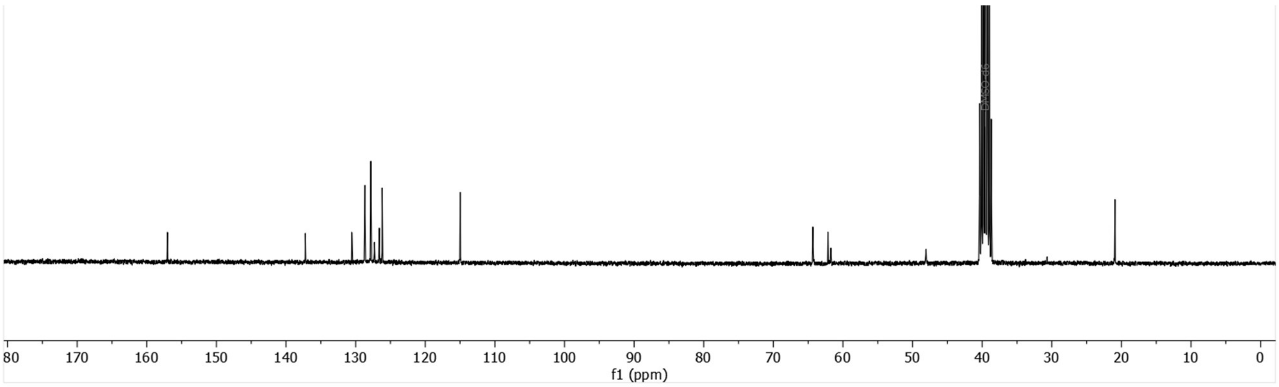


***(E)-4-(2-(N-methyl)piperidiniumethyloxy)stilbene iodide (15).***

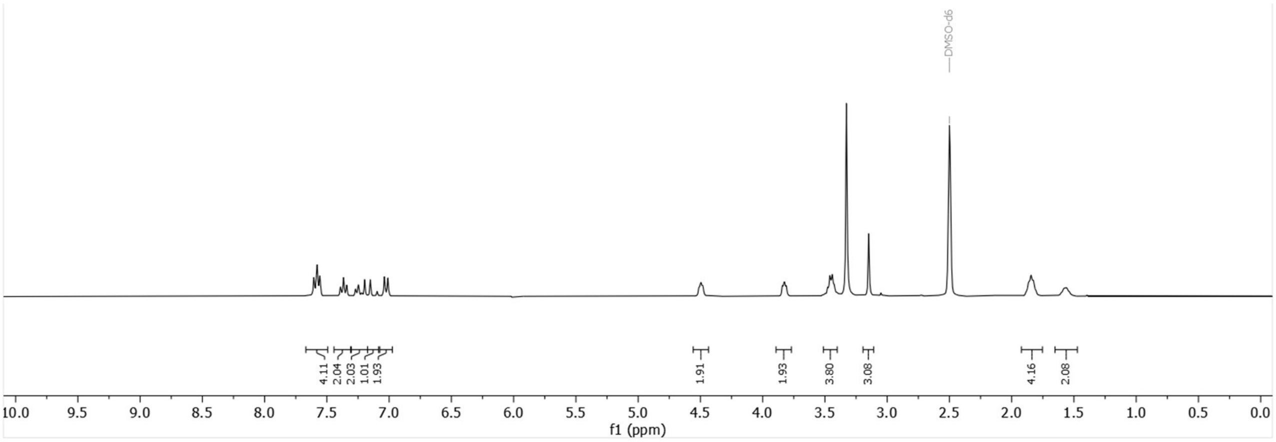


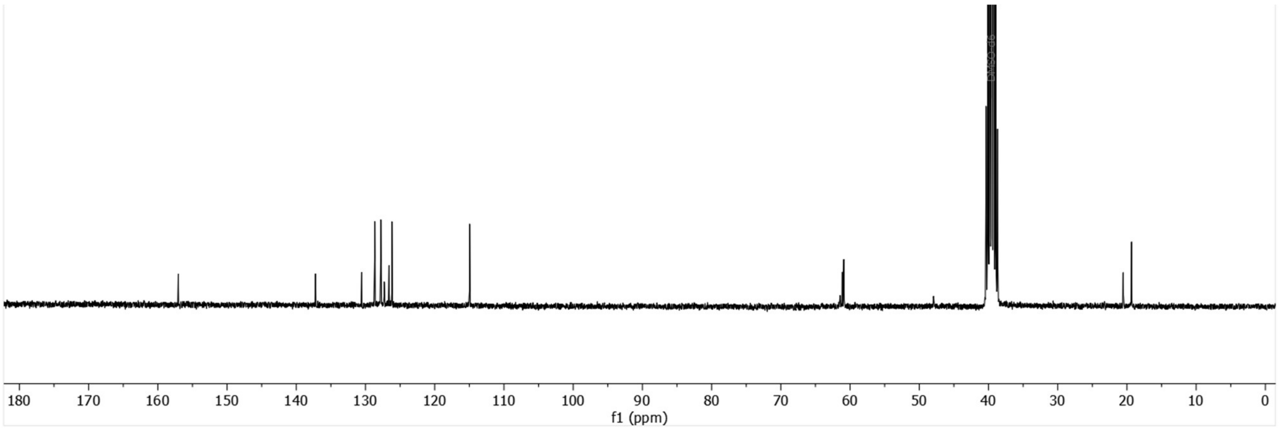

***(E)-4-(2-(N-Methyl)morpholiniumethyloxy)stilbene iodide (16).***

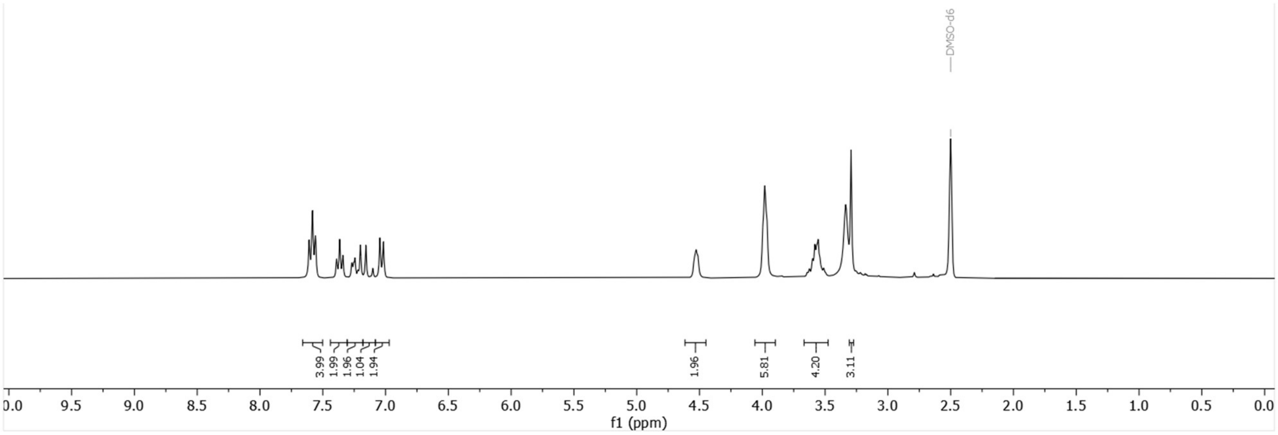


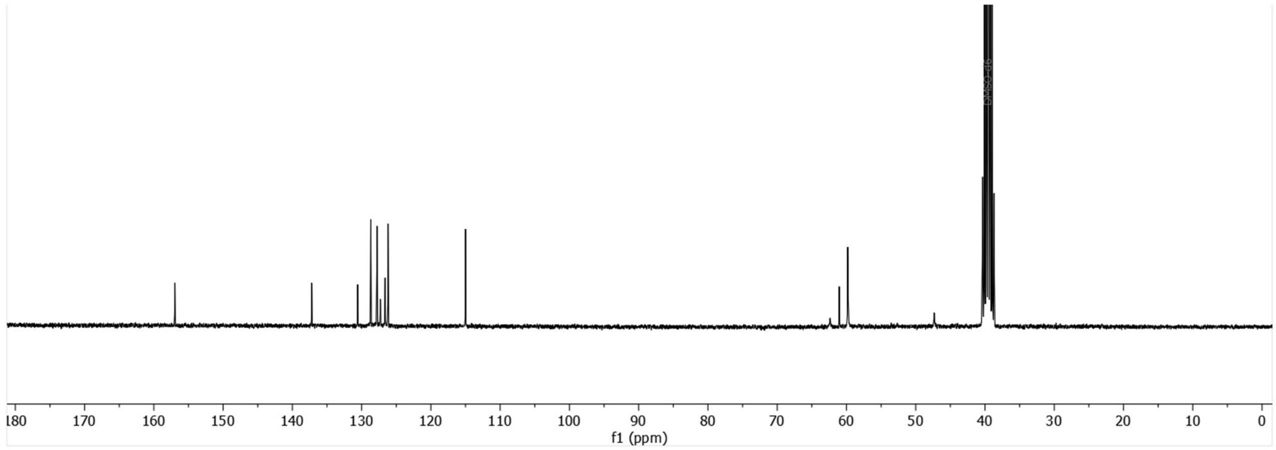


***(E)-4-(2-pyridiniumethyloxy)stilbene iodide (17).***

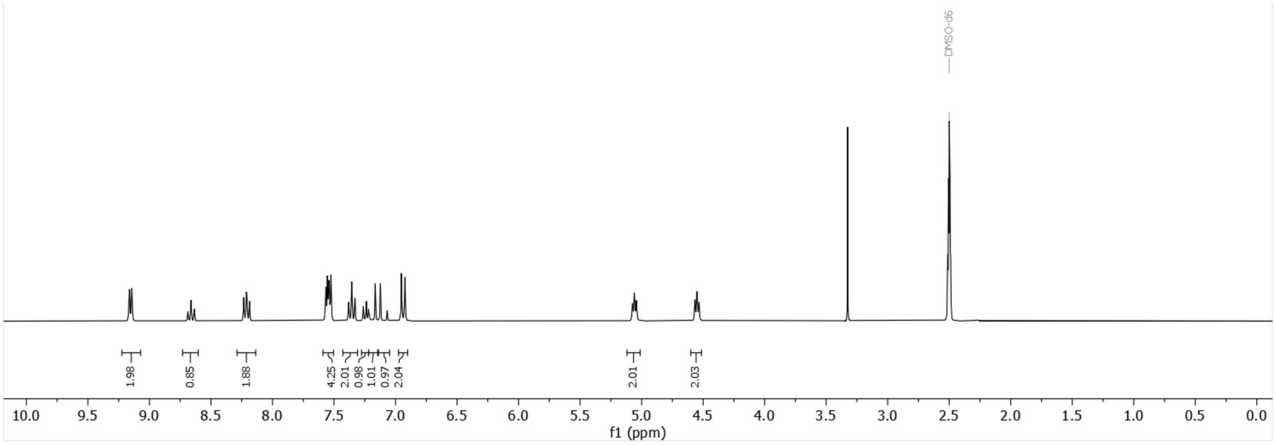

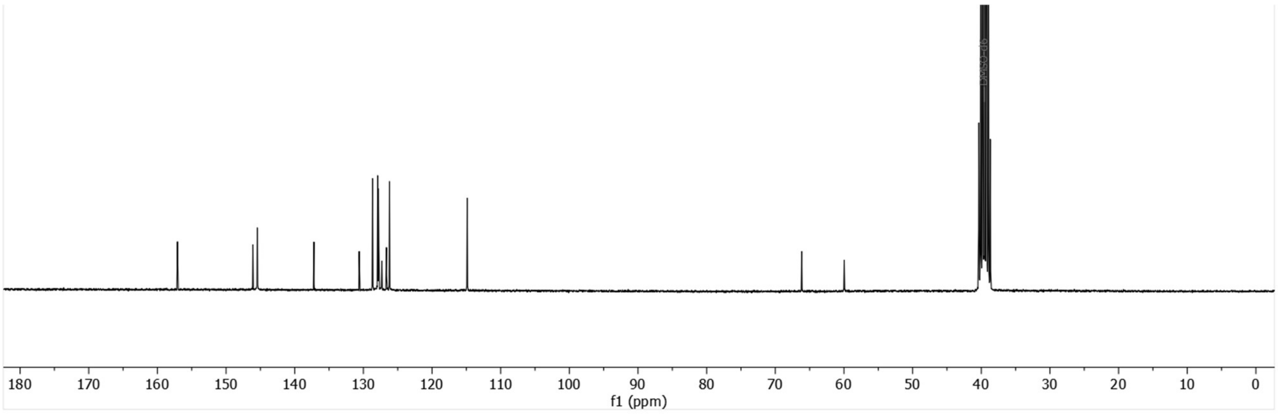


***(E)-4-(3-(N,N-dimethyl-azetidiniumoxy)stilbene iodide (18).***

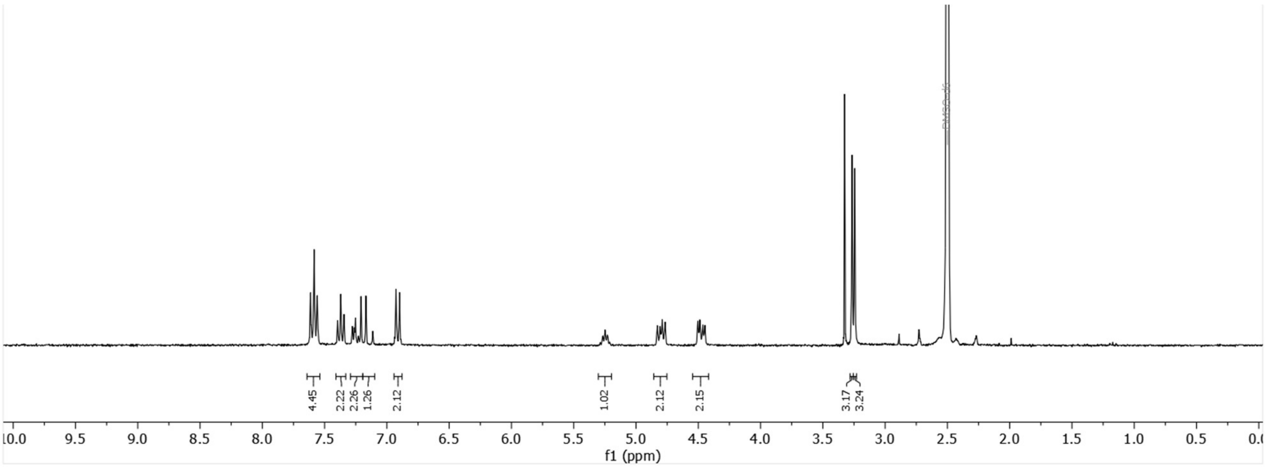


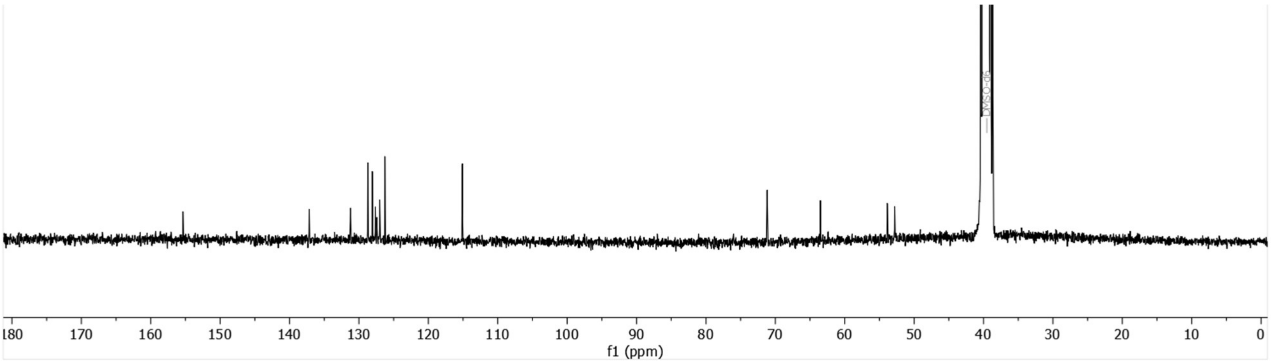

***(±)-(E)-4-(3-(N,N-dimethyl-pyrrolidiniumoxy)stilbene iodide ((±)-19).***

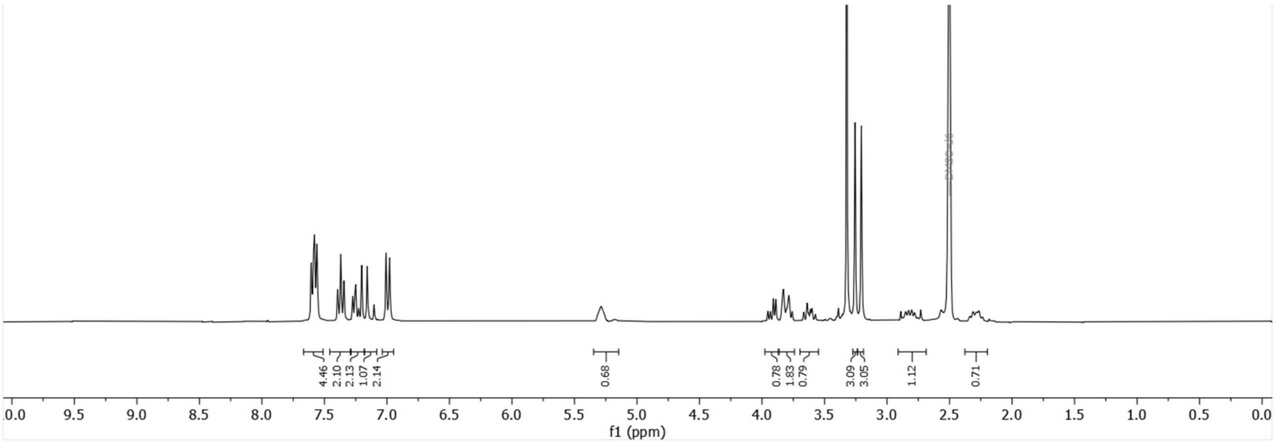


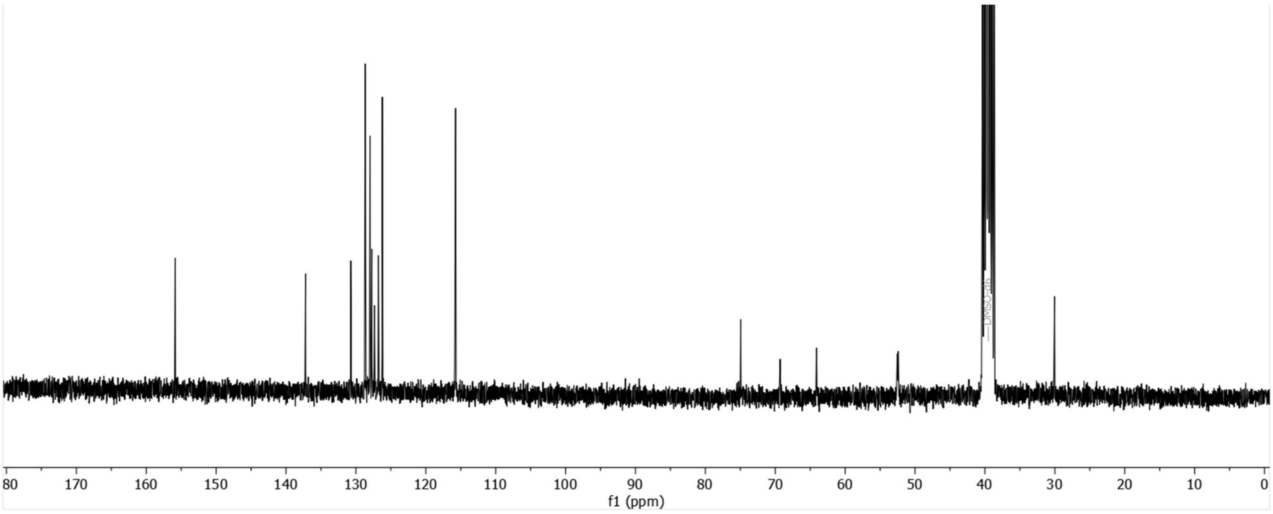


***(S)-(E)-4-(3-(N,N-dimethyl-pyrrolidiniumoxy)stilbene iodide ((S)-19).***

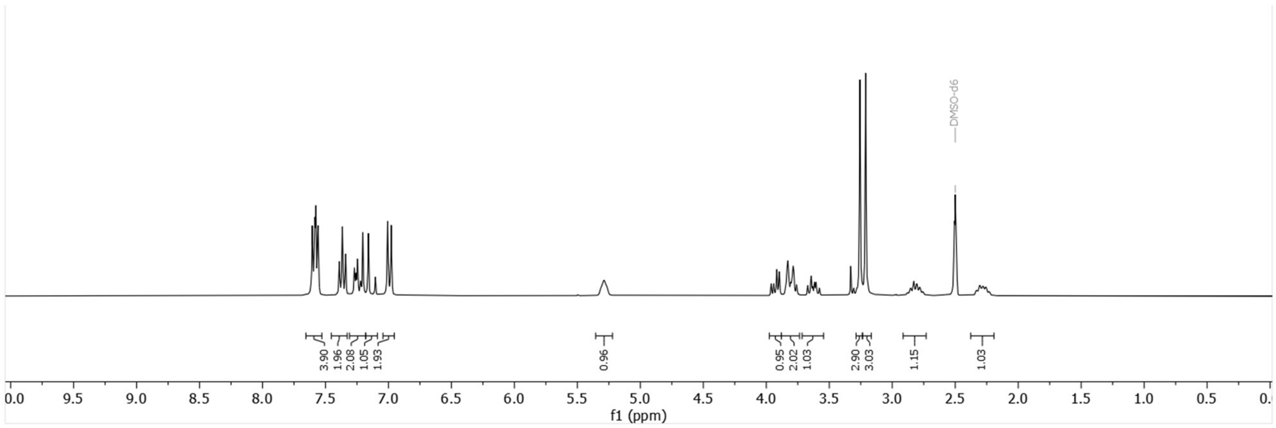

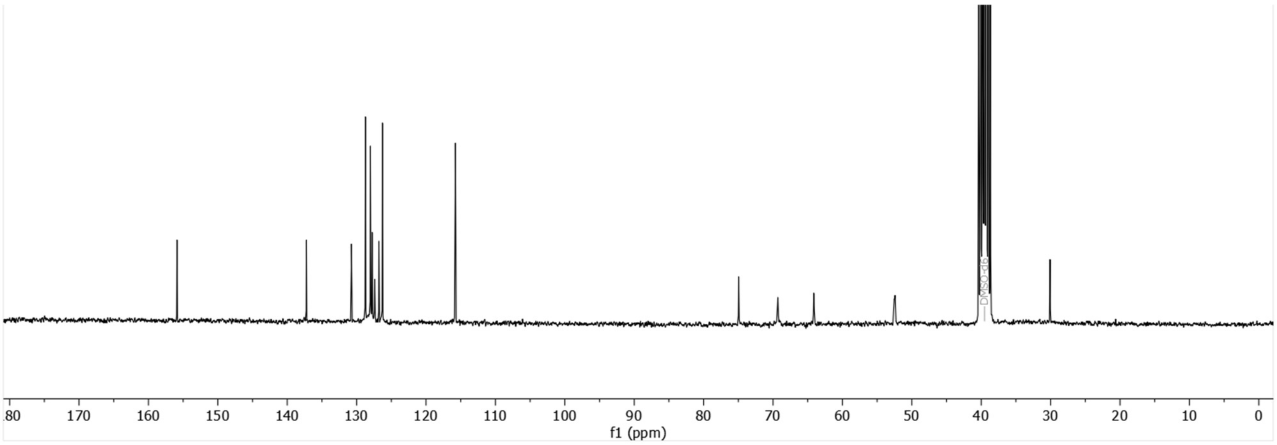


***(R)-(E)-4-(3-(N,N-dimethyl-pyrrolidiniumoxy)stilbene iodide ((R)-19).***

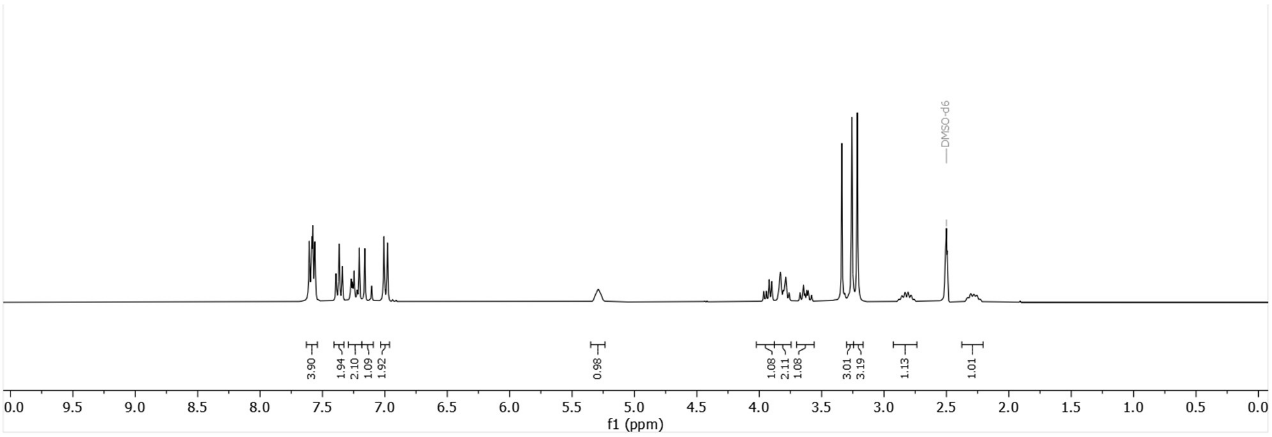


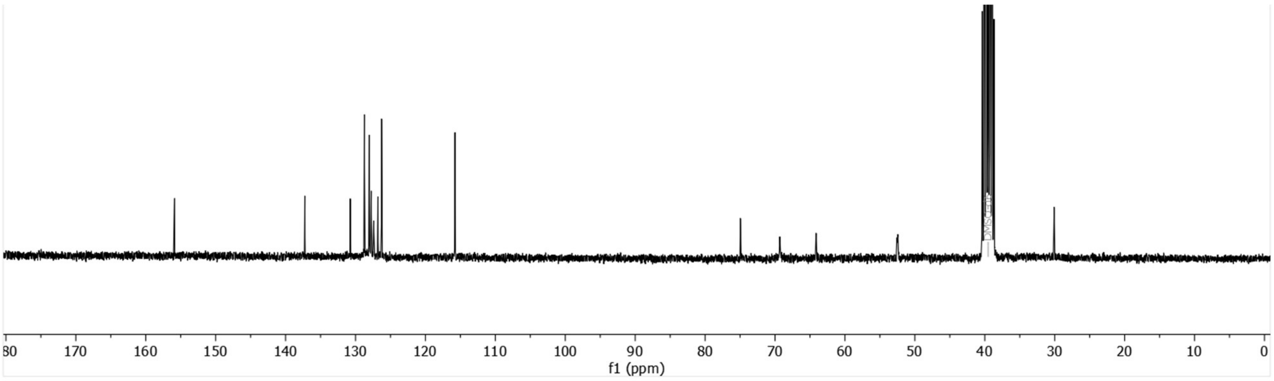

***(R)-(E)-4-(3-(N-ethyl,N-methyl-pyrrolidiniumoxy)stilbene iodide ((R)-20).***

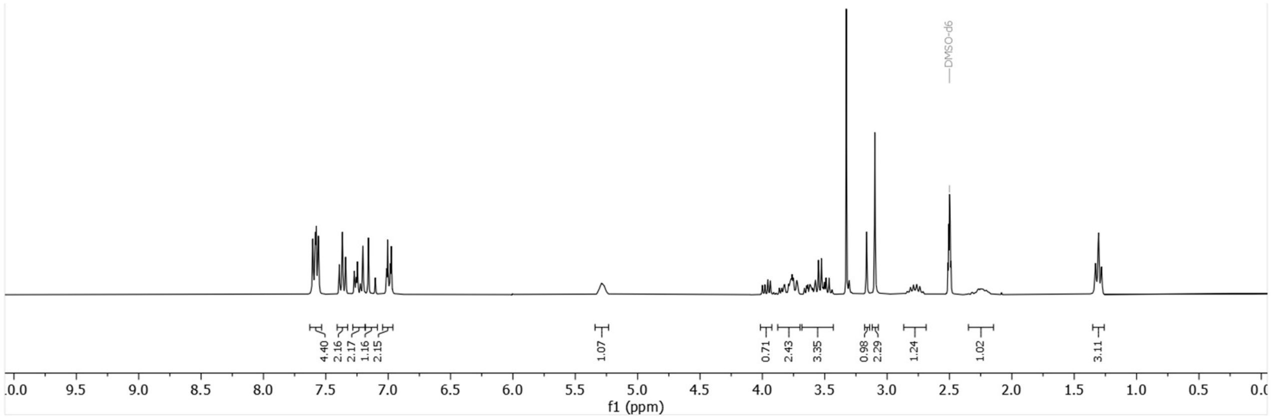


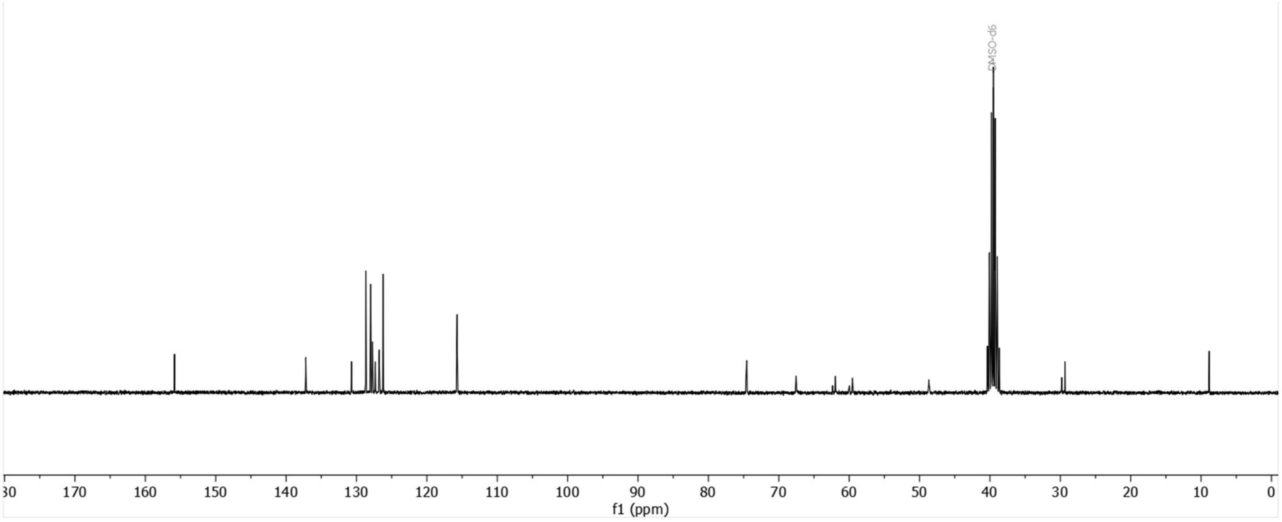


***(R)-(E)-4-(3-(N,N-diethyl-pyrrolidiniumoxy)stilbene iodide ((R)-21).***

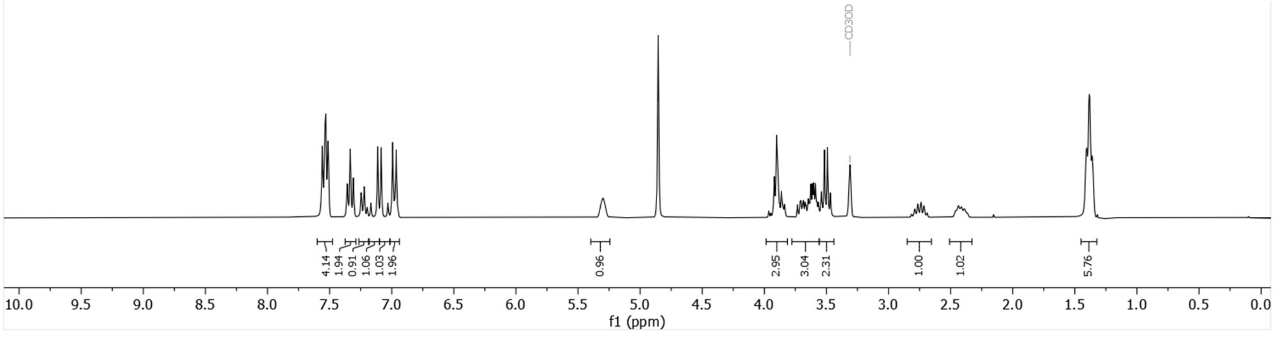

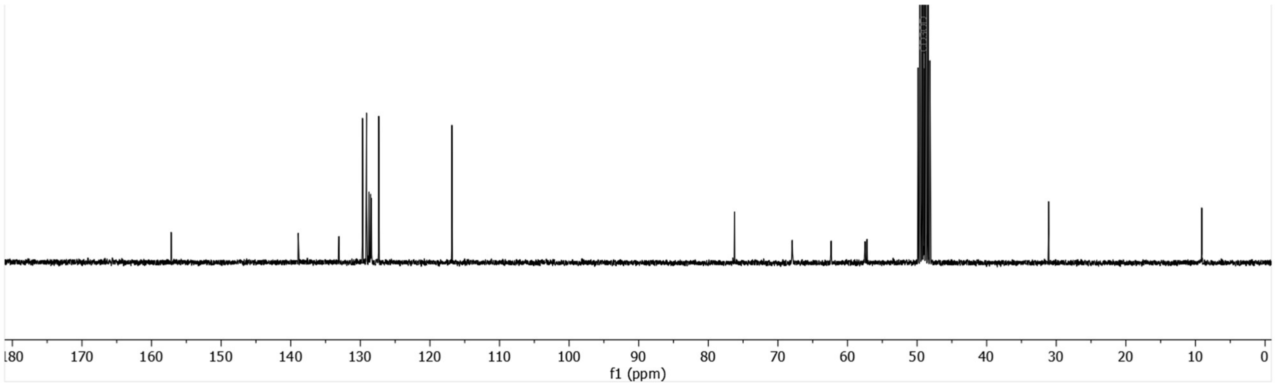


***(±)-(E)-4-(3-(N,N-dimethyl-pyperidiniumoxy)stilbene iodide ((±)-22).***

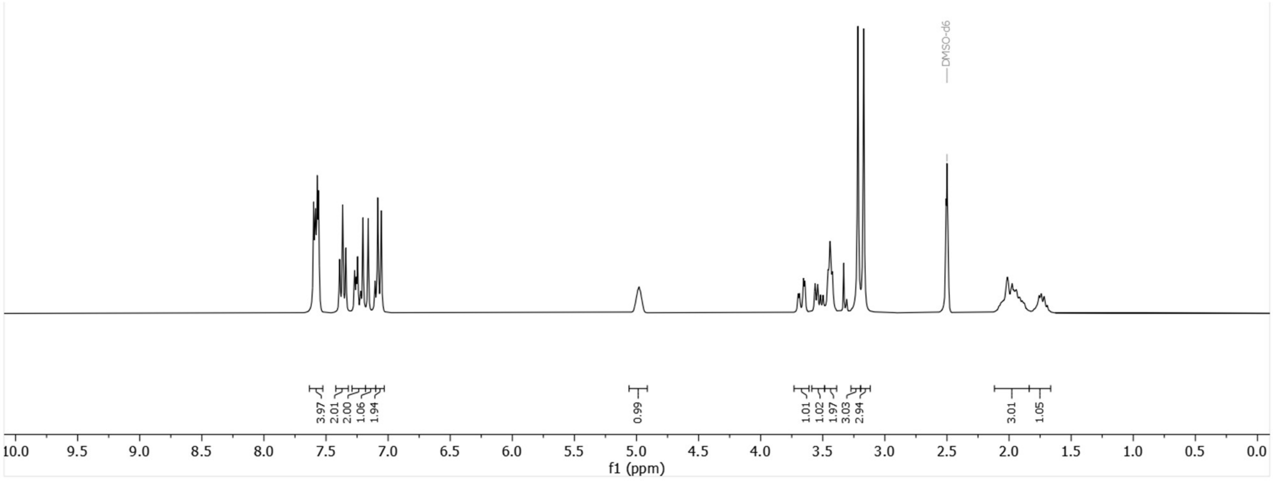


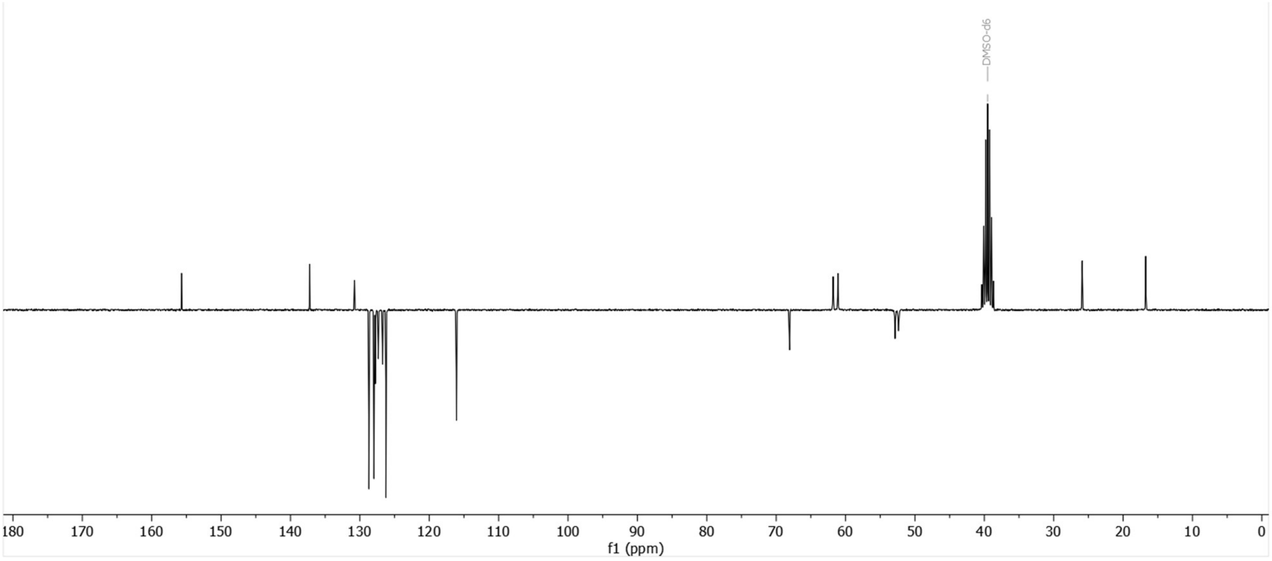

***(E)-4-(4-(N,N-dimethyl-pyperidiniumoxy)stilbene iodide (23).***

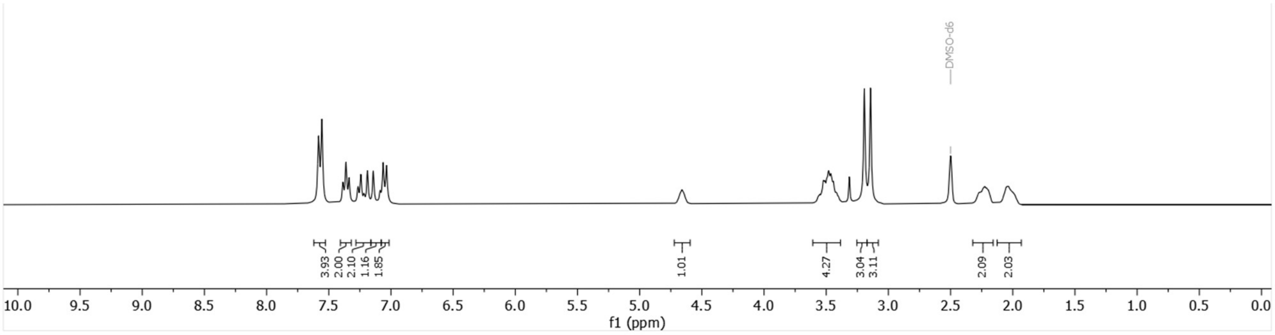


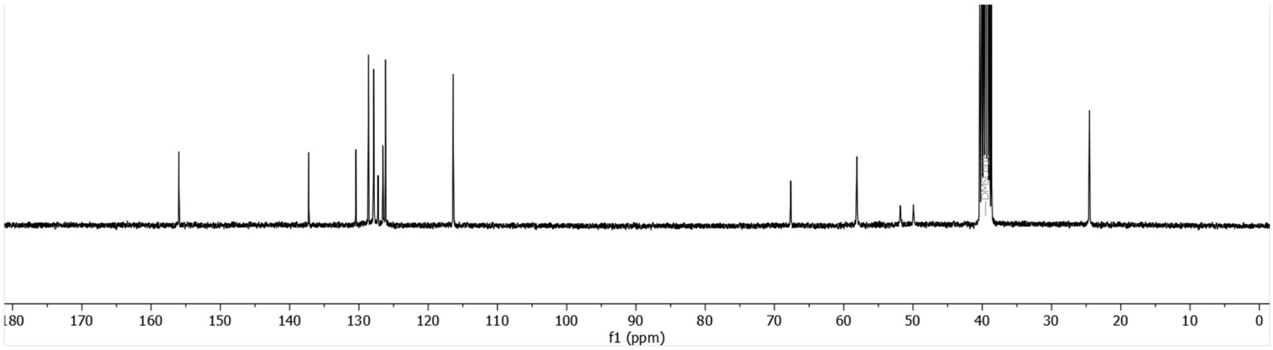


***(±)-(E)-4-(3-(N-methyl-quinuclidiniumoxy)stilbene iodide ((±)-24).***

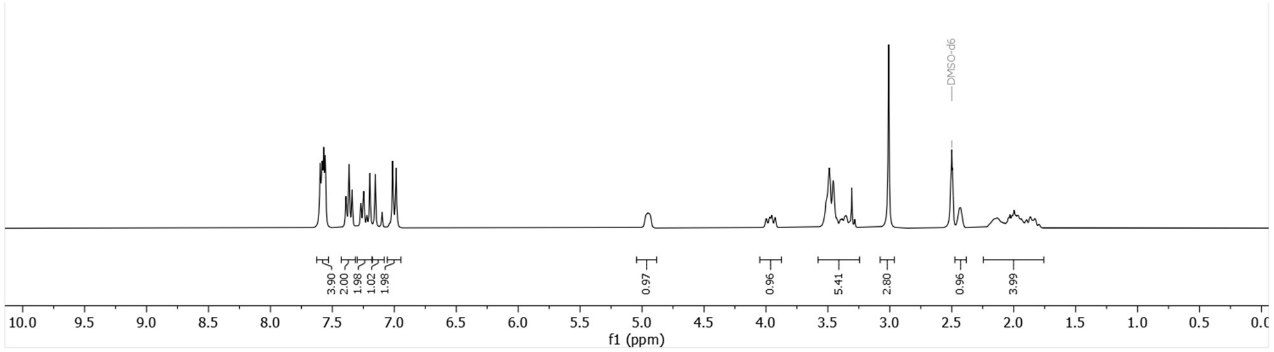


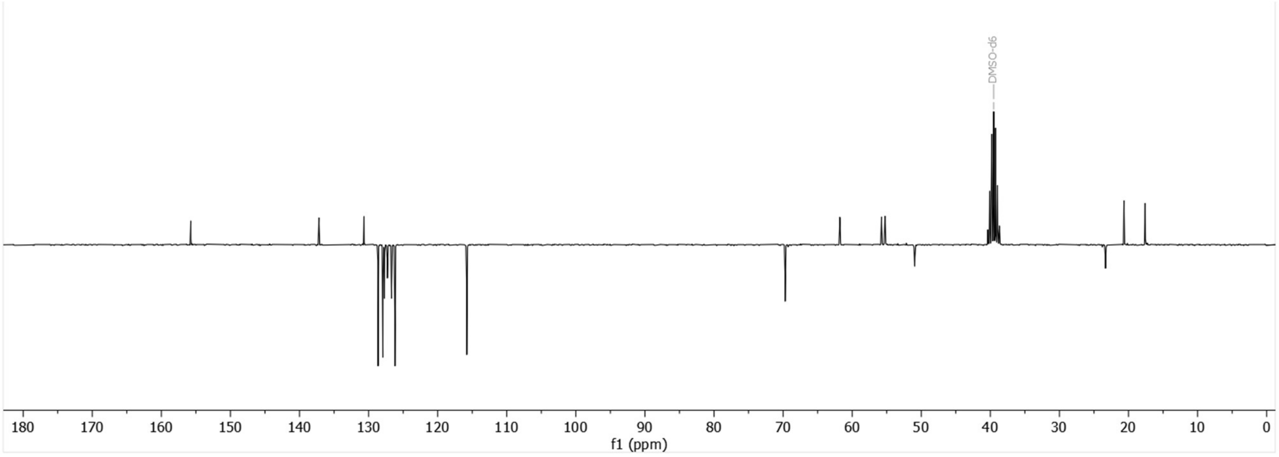

***(S)-(E)-1-methyl-2-(4-stilbenoxymethyl)pyrrolidinium iodide ((S)-25).***

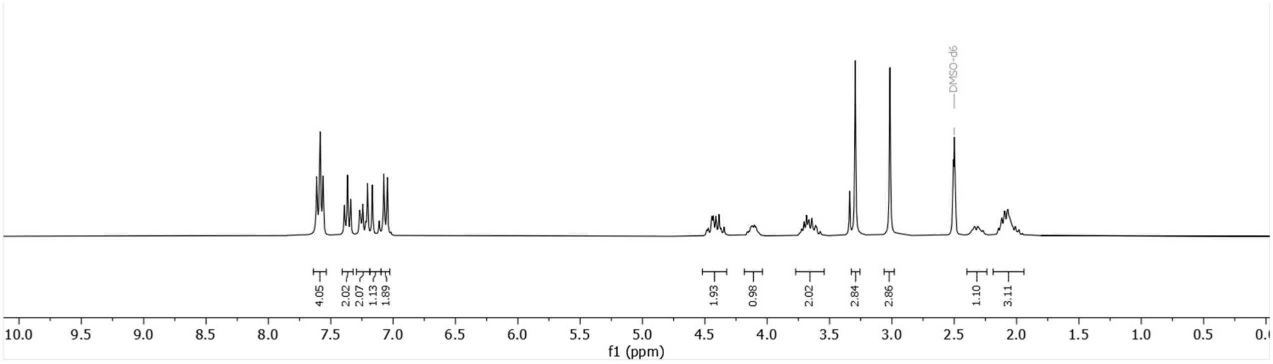


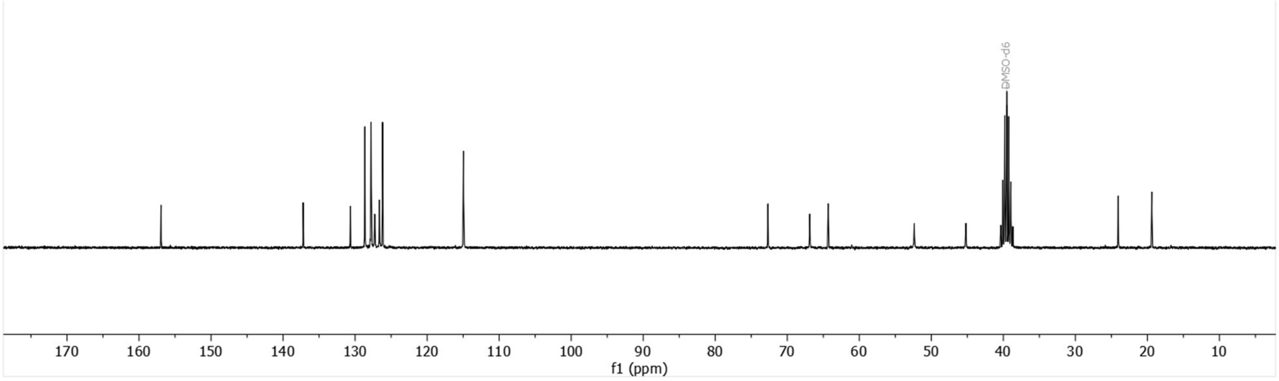


***(R)-(E)-1-methyl-2-(4-stilbenoxymethyl)pyrrolidinium iodide ((R)-25).***

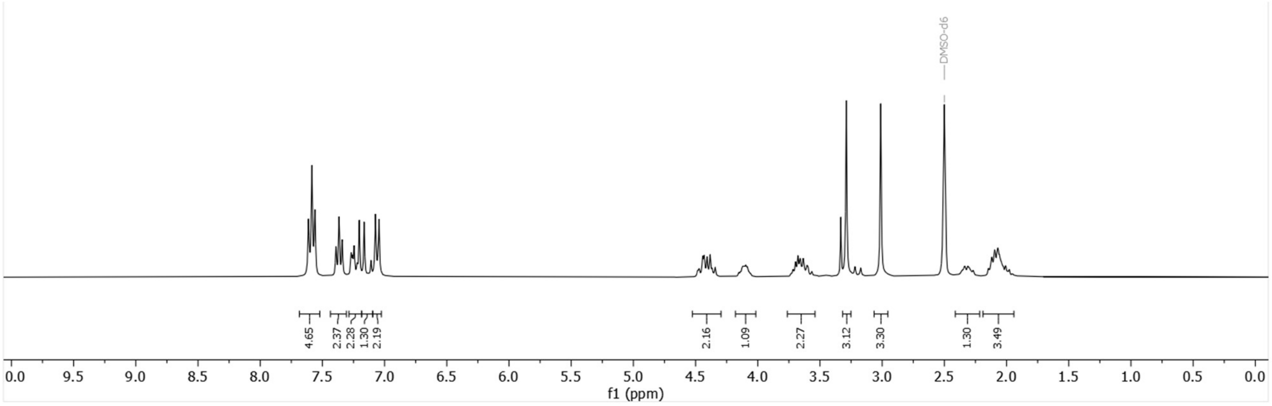


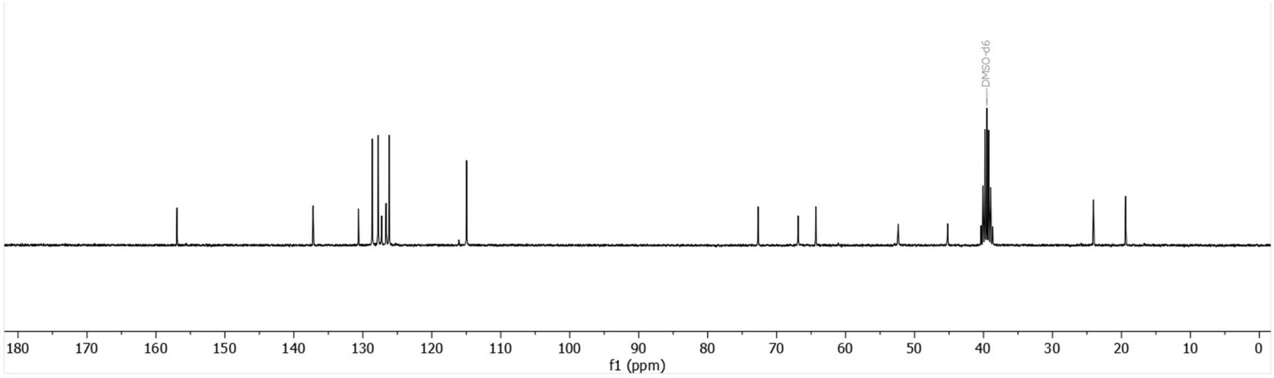

***(±)-(l)-N,N-diethyl-N-methyl-2-(4-((E)-stilbenoxy)cyclopropan-1-ammonium iodide ((±)-26).***

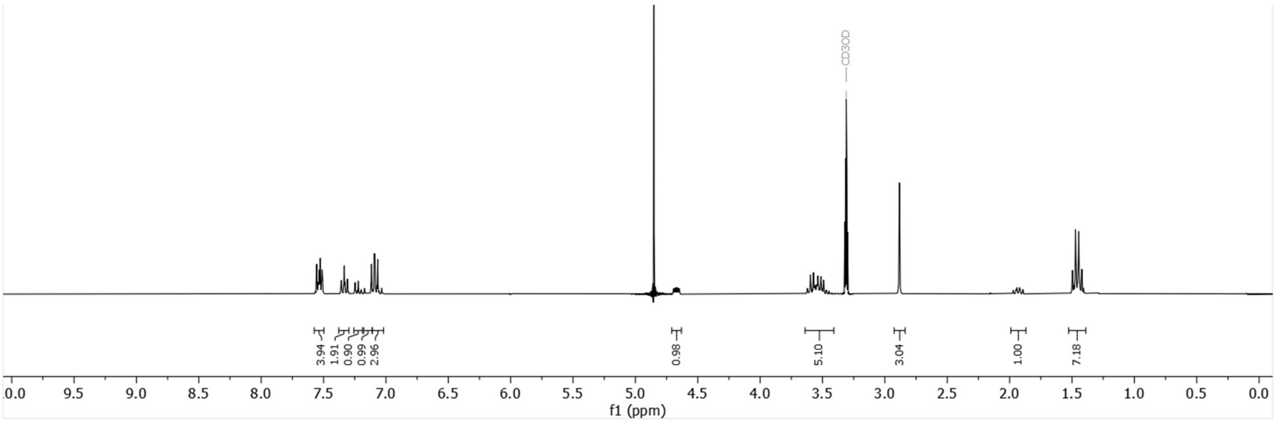


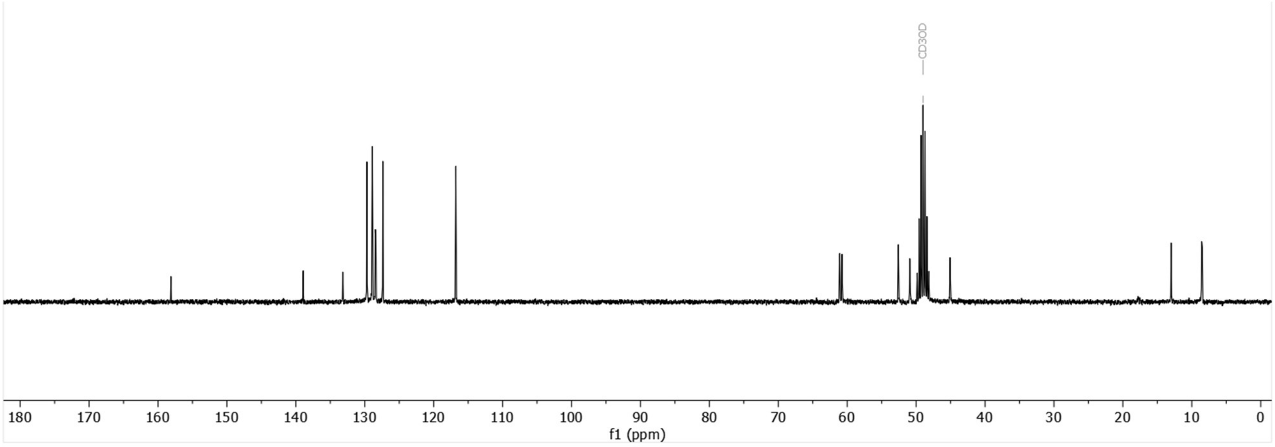


***(±)-(u)-N,N-diethyl-N-methyl-2-(4-((E)-stilbenoxy)cyclopropan-1-ammonium iodide ((±)-27).***

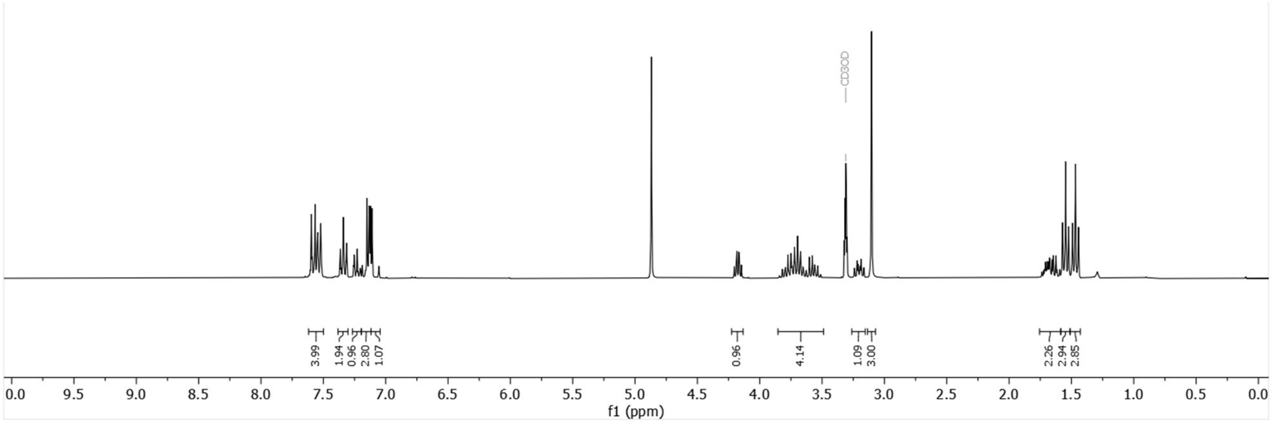

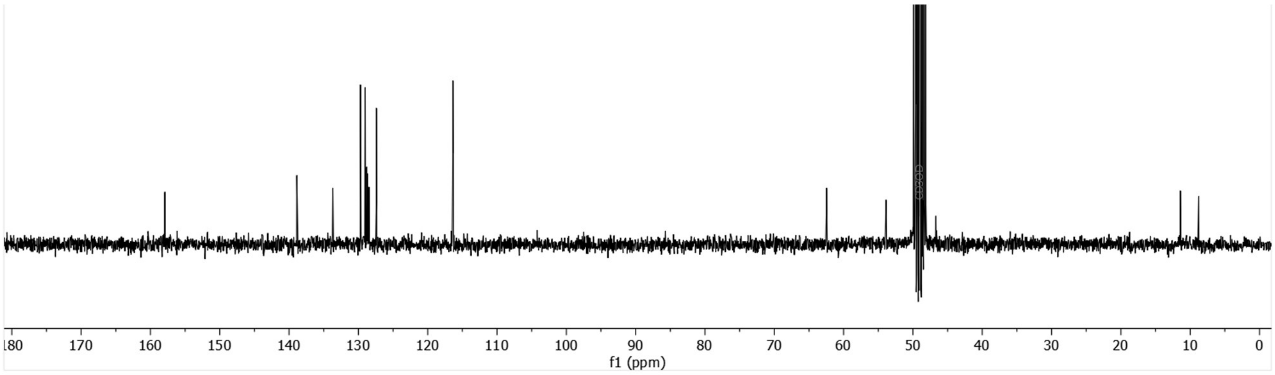
180
170
160
150
140
130
120
110
100
90
80
70
60
50
40
30
20
10
0
f1 (ppm)

HPLC traces

*(E)-4-(2-(trimehtylammonium)ethyloxy)stilbene iodide (**6**).*

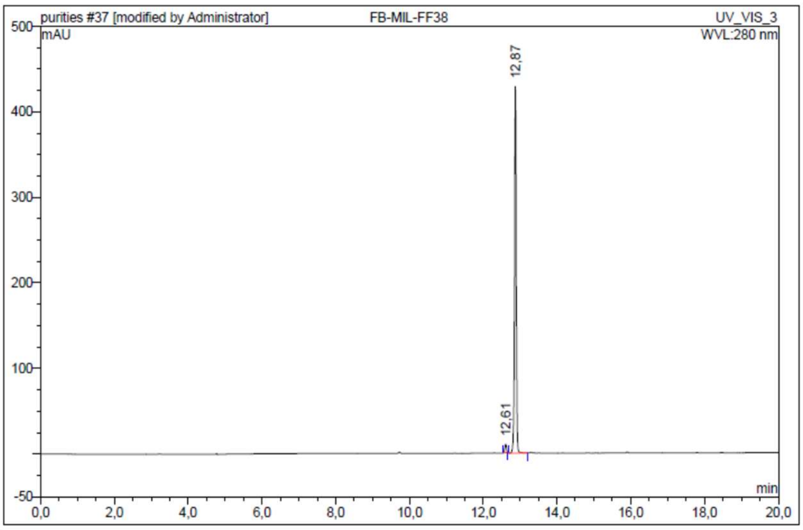


| No. | Ret.Time min | Peak Name | Height mAU | Area mAU*min | Rel.Area % | Amount | Resolution(EP) |
|---|---|---|---|---|---|---|---|
| 1 | 12,61 | n.a. | 10,038 | 0,491 | 2,09 | n.a. | 3,27 |
| 2 | 12,87 | n.a. | 428,432 | 23,064 | 97,91 | n.a. | n.a. |
| Total: | | | 438,470 | 23,555 | 100,00 | 0,000 | |

*(E)-4-(2-(N-cyclohexyl-N,N-dimethyl)ammoniumethyloxy)stilbene iodide (7).*

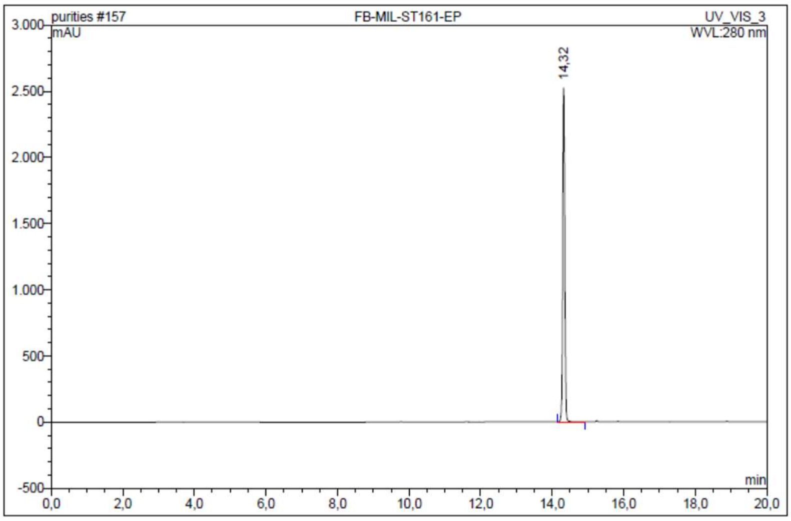


| No. | Ret.Time min | Peak Name | Height mAU | Area mAU*min | Rel.Area % | Amount | Resolution(EP) |
|---|---|---|---|---|---|---|---|
| 1 | 14,32 | n.a. | 2526,670 | 175,243 | 100,00 | n.a. | n.a. |
| Total: | | | 2526,670 | 175,243 | 100,00 | 0,000 | |

*(±)-(E)-4-(3-(N,N-dimethyl-pyperidiniumoxy)stilbene iodide ((±)-**22**).*

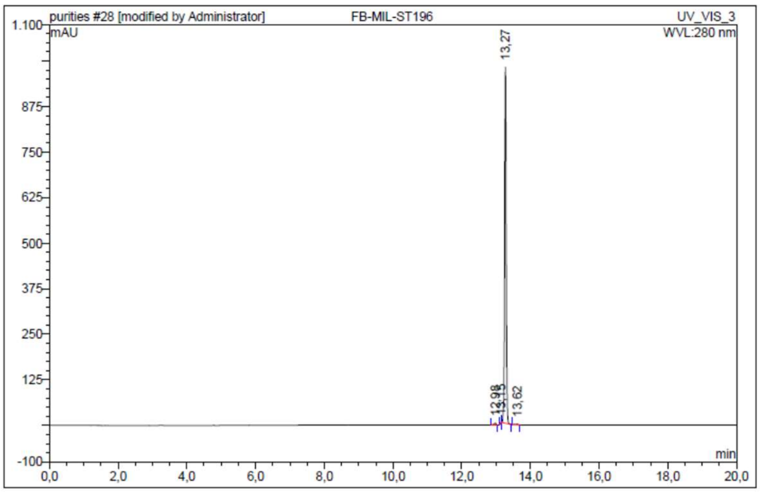


| No. | Ret.Time min | Peak Name | Height mAU | Area mAU*min | Rel.Area % | Amount | Resolution(EP) |
|---|---|---|---|---|---|---|---|
| 1 | 12,98 | n.a. | 4,735 | 0,314 | 0,58 | n.a. | 2,62 |
| 2 | 13,15 | n.a. | 1,407 | 0,046 | 0,09 | n.a. | 1,77 |
| 3 | 13,27 | n.a. | 977,628 | 53,404 | 98,97 | n.a. | 2,24 |
| 4 | 13,62 | n.a. | 1,980 | 0,198 | 0,37 | n.a. | n.a. |
| Total: | | | 985,750 | 53,962 | 100,00 | 0,000 | |

*(E)-4-(4-(N,N-dimethyl-pyperidiniumoxy)stilbene iodide (**23**).*

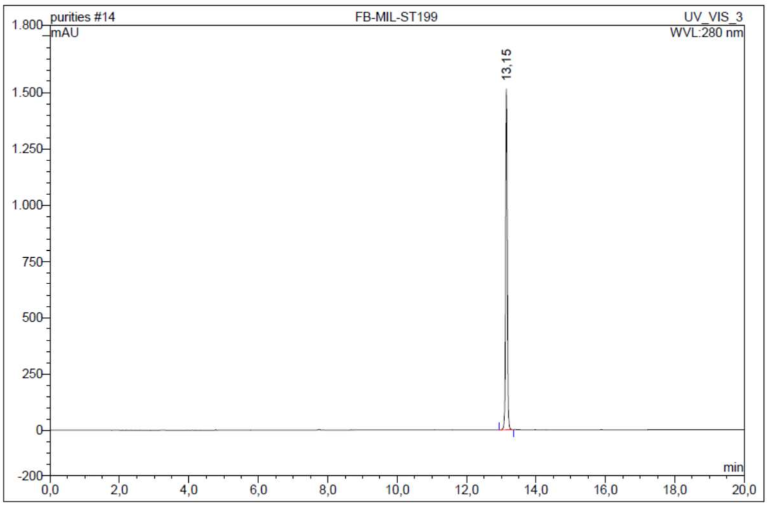


| No. | Ret.Time min | Peak Name | Height mAU | Area mAU*min | Rel.Area % | Amount | Resolution(EP) |
|---|---|---|---|---|---|---|---|
| 1 | 13,15 | n.a. | 1514,311 | 87,240 | 100,00 | n.a. | n.a. |
| Total: | | | 1514,311 | 87,240 | 100,00 | 0,000 | |

*(±)-(E)-4-(3-(N-methyl-quinuclidiniumoxy)stilbene iodide ((±)-**24**).*

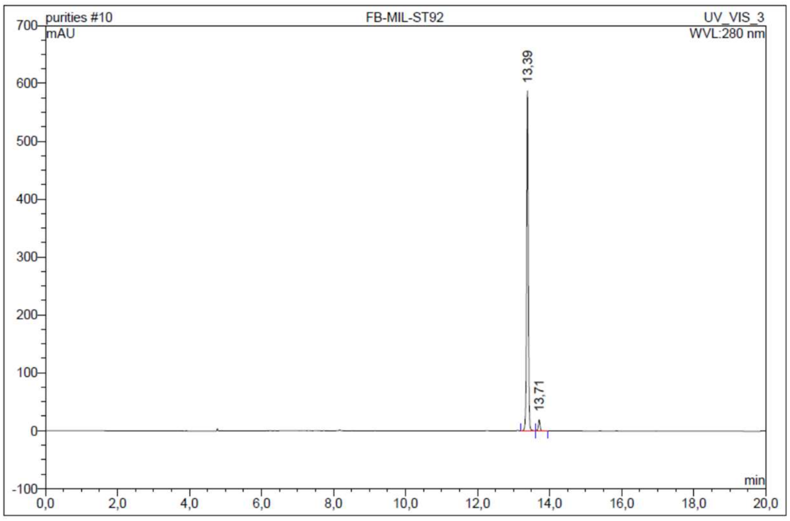


| No. | Ret.Time min | Peak Name | Height mAU | Area mAU*min | Rel.Area % | Amount | Resolution(EP) |
|---|---|---|---|---|---|---|---|
| 1 | 13,39 | n.a. | 587,700 | 32,412 | 96,72 | n.a. | 3,86 |
| 2 | 13,71 | n.a. | 19,198 | 1,100 | 3,28 | n.a. | n.a. |
| Total: | | | 606,899 | 33,512 | 100,00 | 0,000 | |
